# Ovarian Cancers: Genetic Abnormalities, Tumor Heterogeneity and Progression, Clonal Evolution and Cancer Stem Cells

**DOI:** 10.3390/medicines5010016

**Published:** 2018-02-01

**Authors:** Ugo Testa, Eleonora Petrucci, Luca Pasquini, Germana Castelli, Elvira Pelosi

**Affiliations:** Department of Oncology and Molecular Medicine, Core Facilities, Istituto Superiore di Sanità, 00161 Rome, Italy; eleonora.petrucci@iss.it (E.P.); luca.pasquini@iss.it (L.P.); germana.castelli@iss.it (G.C.); elvira.pelosi@iss.it (E.P.)

**Keywords:** ovarian cancer, genetic alterations, clonal evolution, metastases, chemoresistance, cancer stem cells

## Abstract

Four main histological subtypes of ovarian cancer exist: serous (the most frequent), endometrioid, mucinous and clear cell; in each subtype, low and high grade. The large majority of ovarian cancers are diagnosed as high-grade serous ovarian cancers (HGS-OvCas). *TP53* is the most frequently mutated gene in HGS-OvCas; about 50% of these tumors displayed defective homologous recombination due to germline and somatic *BRCA* mutations, epigenetic inactivation of BRCA and abnormalities of DNA repair genes; somatic copy number alterations are frequent in these tumors and some of them are associated with prognosis; defective NOTCH, RAS/MEK, PI3K and FOXM1 pathway signaling is frequent. Other histological subtypes were characterized by a different mutational spectrum: LGS-OvCas have increased frequency of *BRAF* and *RAS* mutations; mucinous cancers have mutation in *ARID1A*, *PIK3CA*, *PTEN*, *CTNNB1* and *RAS*. Intensive research was focused to characterize ovarian cancer stem cells, based on positivity for some markers, including CD133, CD44, CD117, CD24, EpCAM, LY6A, ALDH1. Ovarian cancer cells have an intrinsic plasticity, thus explaining that in a single tumor more than one cell subpopulation, may exhibit tumor-initiating capacity. The improvements in our understanding of the molecular and cellular basis of ovarian cancers should lead to more efficacious treatments.

## 1. Introduction

Ovarian cancer is the fifth-leading cause of cancer death among women in the United States. In 2010 there was an estimated 21,880 new cases and 13,850 deaths from ovarian cancer in the United States [1]. In 2017 there will be an estimated 22,140 new cases and 14,080 deaths from ovarian cancer in the United States (National Cancer Institute, Cancer Stat Facts: Ovarian Cancer). In the last years, the number of new case of ovarian cancer was 11.7 per 100,000 women per year and the number of deaths was 7.4 per 100,000 women per year. It was estimated that about 1.3% of the women will be diagnosed with ovarian cancer at some point during their lifetime. Ovarian cancer rates are highest in women aged 55–64 years. The median age of death from ovarian cancer is 70 years. Although the 5-year survival rate is >90% for women with early-stage ovarian cancer, about 80% of women present with late-stage disease and the survival at 5 years for patients with regional and distant disease diffusion was 73% and 29%, respectively; thus, the survival at 5 years for the whole population of ovarian cancer patients was 46.5%. Most deaths are of patients presenting with advanced-stage, high-grade serous ovarian cancer. The standard treatment is aggressive surgery followed by platinum-taxane chemotherapy. After therapy, platinum resistant disease recurs in many patients and the overall five-year survival probability is only about 30%. In spite, all the limitations of the current therapeutic approach, the development of the standard treatment has determined an increase of the 5-year survival from 33.7% in 1975 to 46.6% in 2009. Finally, it is important to point out that ovarian cancer is a malignancy whose incidence is decreasing in the last decades, moving from 15 new case per 100,000 women to 11.7 per 100,000 women in 2014.

Ovarian cancer is a very heterogeneous disease and is mainly represented by epithelial tumors that develop from ovarian epithelial surface cells, giving rise to four main distinct histological subtypes: serous, the most frequent; endometrioid; mucinous and clear cells. In some cases, it is present a mixture of these histological types. Each of these histological subtypes may exhibit a different degree of differentiation: well differentiated, grade 1; moderately differentiated, grade 2 and poorly differentiated, grade 3.

Ovarian cancers are basically subdivided into two types according to their invasiveness and aggressiveness: low-grade type 1 and high-grade type 2 (Table 1). Type 1 cancers account for only 10% of the death from ovarian cancer, are slowly growing and include low-grade serous, endometroid and mucinous tumors; type 2 cancers are rapidly progressing and include high-grade serous carcinomas [2]. The histological serous subtype accounts for 60–80% of all cases of epithelial ovarian cancer, but only 25% of these cases are detected at an early stage (either stage I or II). Expression profiling studies have validated this subdivision of ovarian cancers and have further supported the identification of a low-grade and high-grade clusters of tumors. Furthermore, genomic grade clusters have shown different types of recurrent mutations in these two tumor clusters: type I tumors are rarely associated with *TP53* mutations, while type II tumors are very frequently associated with *TP53* mutations (Table 1). This initial dualistic model of ovarian cancerogenesis was recently revised and expanded. Particularly, in this new revised dualistic model, type I cancers are divided into three groups (Table 1): (1) endometriosis-related tumors form a group including endometrioid, clear cell and seromucinous carcinomas; (2) low-grade serous carcinomas; (3) mucinous carcinomas and Brenner tumors [3]. Another very important difference between type I and type II tumors is related to their different tissutal origin, in that type I tumors develop from benign extraovarian lesions that implant on the ovary and which can switch subsequently to a malignant genotype/phenotype, while type II tumors develop from intraepithelial carcinomas originated from Fallopian tube secretory cells or progenitor cells [3]. Finally, genetic features separate type I from type 2 tumors: type 1 tumors exhibit a relative genetic stability, while type II tumors display chromosomal instability; as mentioned above, TP53 mutations are relatively rare in type I tumors, while they are frequent in type II tumors; some mutations involving the *PIK3CA, PTEN, ARID1A, KRAS* and *BRAF* are frequent in type I tumors, while other mutations involving RB1, FOXM1, NOTCH 3 pathway and in homologous recombinant repair are frequent in type II tumors [3].

HGS-OvCa is largely the most frequent epithelial ovarian cancer, responsible for most of the deaths caused by this cancer. The highest incidence rates of HGS-OvCa are observed in the most developed parts of the world, including North America and Eastern Europe, where the incidence of this tumor exceeds 8 cases per 100,000. Intermediate/low rates are observed in Asia and Africa, respectively. Hormonal and reproductive factors are involved in the pathogenesis of HGS-OvCa. Basically two hypotheses have been proposed: (a) the so-called “incessant ovulation” hypothesis proposes that the lifetime number of ovulatory cycles increases the risk of developing ovarian cancer because increases the rate of cellular division associated with the repair of the surface epithelium after each ovulation and, thus, increases the risk of spontaneous somatic mutations; (b) the so-called “gonadotropin hypothesis” attributes to gonatropins, such as follicle-stimulating hormone and luteinizing hormone a stimulatory role on ovarian cancer formation (reviewed in [4]). According to these hypotheses, early age menarche and late age of menopause enhance the risk of developing ovarian cancer. Numerous studies have investigated the possible link between pregnancy and ovarian cancer risk, showing that parous women have a 30–60% lower risk than nulliparous women of developing ovarian cancer, in line with the capacity of pregnancy to cause anovulation and to suppress secretion of pituitary gonadotropins; similarly, lactation, that suppresses secretion of pituitary gonadotropins and leads to anovulation, reduces the risk of developing ovarian cancer [4].

The present review is focused to provide a comprehensive view of the tremendous progresses made in the last three decades in defining the main genetic and molecular abnormalities of various types of ovarian cancers, showing the existence of a set of gene mutations/alterations characterizing each ovarian cancer subtype. These studies have provided also a fundamental support to understand the cellular origin of these tumors and to identify in many cases the corresponding precursor lesions, a step essential to improve the procedures of cancer prevention/early detection. Unfortunately, these consistent progresses in basic science of ovarian cancers, do not have been translated into the expected progresses at therapeutic level, particularly for HGS-OvCa. This review tries to explain the difficulties existing and hampering the translation into more efficacious therapies. In spite these limitations, some topics under investigation at clinical level are promising and will be analyzed in detail.

Surgical debulking plays an essential role in the treatment of HGS-OvCa. For all the stages of disease (and particularly for stages from I to III) it is of fundamental importance to remove all visible tumor masses and to perform accurate sampling of lymph nodes and tissues from fixed locations throughout the abdominal cavity. This procedure is of fundamental importance to allow a careful staging of the tumor according to the TNM (Tumor-node-metastasis) or the FIGO (International Federation of Gynecology and Obstetrics) classification (Table 2).

## 2. Genetic Abnormalities of Serous Ovarian Cancers

### 2.1. High-Grade Serous Ovarian Cancer

**Figure 1:**
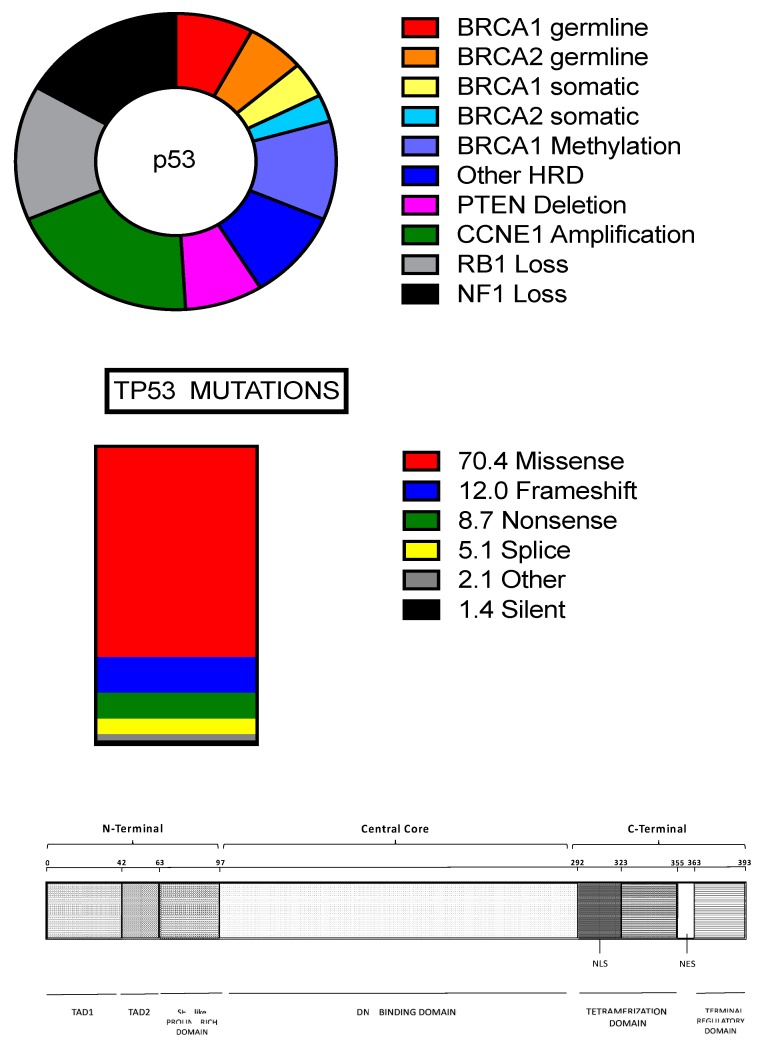
(**Top Panel**): Mutational spectrum of high-grade serous ovarian cancer (HGS-OvCa). In the figure are reported some of the recurrent genetic abnormalities observed in HGSOC. In the middle of the circle of the figure, *TP53* mutations are indicated, occurring in virtually 100% of patients; (**Middle Panel**): Different types of *TP53* mutations and their percentages in HGS-OvCa; (**Bottom Panel**): Structure of TP53 protein: the different structural and functional domains of the protein are reported. TAD1 and TAD2: Tans Activation Domains 1 and 2; NLS: Nuclear Localization Signal; NES: Nuclear Esportation Signal.

As mentioned above, type I tumors rarely exhibit *p53* mutations, but typically show mutations in some genes, involving *BRAF, KRAS*, *PTEN*, *CTNNB1* and *PIK3CA*. Many of these genes encode for upstream regulators of the MAPK pathway and therefore is not surprising that their mutation results in the constitutive activation of this signaling pathway (observed in about 70% of these tumors). In about 10% of these tumors Her2/neu mutations have been observed: these mutations are mutually exclusive with *BRAF* and *KRAS* mutations. It is important to note that point mutations are not frequent in low-grade serous carcinomas; in these tumors, the genes showing the most frequent mutations were *BRAF* and *KRAS*, occurring in 38% and 19% of cases, respectively [5]. Type II high-grade serous tumors are characterized by genetic instability and high frequency of DNA copy number gain or losses. These tumors rarely contain *KRAS* and *BRAF* mutations. High-grade serous carcinomas present a high degree of invasiveness at diagnosis involving bilaterally the ovarian surface and the peritoneal membranes with rapid onset of carcinomatosis: this condition greatly restricts the possibility of surgery resection that remains limited to a surgical debulking. Some germ-line mutations, particularly those involving the genes *BRCA1* and *BRCA2*, harbor an increased risk of developing a high-grade serous ovarian cancer (HGS-OvCa) (approximately 13% of these tumors is attributable to germline mutations in BCRA1/2). A recent study provided detailed data on messenger RNA expression, microRNA expression, promoter methylation, DNA copy number and the DNA sequences of exons from coding genes in 316 high-grade serous ovarian carcinomas [6]. This important study provided evidence that this type of cancer is characterized by *TP53* mutations in almost all tumors (96%); few additional genes are recurrently mutated in HGS-OvCas, but at a much lower frequency than TP53: *BRCA1* about 12.5% (9% of germline mutation and 3.5% somatic mutations), *BRCA2* about 11.5% (8% germline mutations and 3.3% somatic mutations), *CSMD3* 6%, *NF1* 4%, *CDK12* 3%, *GABRA6* 2% and *RB1* 2% (Figure 1) [6]. In contrast, significant focal copy number aberrations are much more frequent (113 copy number alterations were identified) [6]. The most common focal amplifications encoded *CCNE1* (Cyclin E1), *MYC* and *MECOM*, each being highly amplified in more than 20% of cases; interestingly, 22 genes that are therapeutic targets, including *MECOM, MAPK1, CCNE1* and *KRAS* are amplified in at least 10% of the cases (Figure 1) [6]. Importantly, the integrated analysis combining mutational data, copy number changes or changes in gene expression provided evidence about the main pathways altered in HGSC: RB1 and PI3K/RAS pathways were deregulated in 67% and 45% of cases, respectively; the NOTCH signaling pathway was altered in 22% of cases [6]. A very interesting observation was that the homologous recombination pathway was altered in 51% of cases: 20% of cases had germline or somatic mutations in *BRCA* 1–2, 11% lost *BRCA1* expression through DNA hypermethylation (this methylation abnormality is mutually exclusive of *BRCA1* mutations), 8% had amplification of *EMSY*, focal deletion or mutation of *PTEN*, 3% hypermethylation of *RAD51C*, 2% mutation of *ATM* or *ATR*, and 5% mutation of Fanconi anemia genes [6]. This observation was particularly important because represented a rationale for clinical trials based on the use of PARP inhibitors targeting HSG-OvCas with the homologous-recombination-related aberrations [6]. Finally, another interesting observation was that *CCNE1* amplification was much more frequent among BRCA wt samples (26%) than among BRCA-altered cases (8%) [6]. Gene array profiling analysis provided evidence about four HGS-OvCa subtypes: “immunoreactive”, “differentiated”, “proliferative” and “mesenchymal” [6].

Copy number changes or changes in gene expression showed that the RB1 and PI3K are frequently deregulated in HGS-OvCa. Finally, a detailed analysis of the gene expression profile showed that the FOXM1 transcription factor pathway is frequently activated in HGS-OvCa [6]. Copy number analysis studies identified the PIK3/AKT as the most frequently altered cancer-related pathway in epithelial ovarian cancers [6]. Survival analysis provided evidence that copy number gains of *PIK3CA*, *PIK3CB* and *PIK3K4* in these tumors were associated with decreased survival [6]. At the protein level, overexpression of PIK3CA product p100a and p-AKT was associated with decreased survival [6]. In another study, patients with advanced ascitic ovarian cancer have been explored, providing further evidence in favor of very frequent abnormalities of the PI3K signaling pathway: *PI3KCA* mutations were present in 5% of samples; amplification of *PI3KCA* and *AKT2* and deletion of *PTEN* were seen in 12%, 10% and 27% of samples, respectively [7]. Furthermore, p-p70S6K levels were markedly elevated in a high percentage of patients who did not respond to subsequent chemotherapy [8]. These observations suggest an important role for PIK3 pathway in ovarian cancer pathogenesis, indicating its role as a potential target for therapy.

It is important to note that the cancer Genome Atlas study on genetic and epigenetic alterations in 489 cases of HGS-OvCa identified PTEN alterations in only 7% of cases [6]. However, immunochemistry-based studies carried out in smaller cohorts of patients have reported much higher frequencies of *PTEN* alterations, with loss of *PTEN* expression observed in 15% and partial loss in 50% of cases (reviewed in [9]). A recent study provided an explanation for this discrepancy, showing that the various presence of a stromal component strongly biases estimates of *PTEN* expression. Thus, an unbiased analysis carried out using tissue microarray from a large cohort of patients showed the PTEN loss or downregulation occurred in 50–75% of cases [9]. Importantly, PTEN loss was associated in these tumors with expression of the androgen receptor [9].

As mentioned above, HGSCs are characterized by frequent and wide-spread gain and loss of copy number involving large numbers of genes. Some of these copy number alterations (CNAs) seem to be functionally relevant. Some CNAs involve well known driver genes such as *MYC* and *CCNE1*, present in a subset of HGS-OvCas, but the identity of the driver genes present in the 63 focal and recurrent regions of amplification remains at large extent undefined. Some recent studies have identified some amplifications and in few cases, some amplified genes are relevant for ovarian cancer development. In this context, a recent study identified GRB2-associated binding protein 2 (GAB2) as a recurrently amplified (44% of cases) gene that is able to transform immortalized ovarian and Fallopian tube secretory epithelial cells [10]. GAB2 is a signaling intermediate in SHP2-dependent activation of MAPK signaling and activation of PI3K. PI3K activation is required for GAB2-mediated transformation and GAB2-overexpressing cells are sensitive to PI3K inhibition [10]. Another study showed that amplifications of chromosomal region 14q32.33 are associated with a reduced overall survival and progression-free survival of ovarian cancer patients treated with standard platinum-based therapy [11]. This chromosome region contains the putative driver gene AKT1 [11]. Recently, a subgroup of ovarian cancer patients bearing various focal amplifications of chromosome 19 and corresponding to about 26% of total patients was shown to be associated with poor prognosis [12]. Interestingly, two of these focal amplifications, 19p13 and 19q12 were found to be less frequent in *BRCA1/BRCA2*-mutated tumors compared to those wild-type for these two mutations [13].

After the initial TCGA study in 2011, other studies have confirmed that copy number alterations dominate HGS-OvCa genomic landscape. Each of these copy number alterations reflects an amplification or a deletion of a piece of DNA, often involving many kilobases or, even, segments of, or entire, chromosomes or chromosome arms. Each of these copy number alterations entails a structural modification involving many genes (even hundreds of genes) and for these reasons, the evaluation of its biologic consequences is complex and difficult to assess. Computational analysis of the data sets generated by TCGA for various tumors, including ovarian cancers, showed that usually amplifications involve chromosome regions with a high density of oncogenes or, alternatively, of genes involved in epigenetic regulation, while deletions frequently encompass chromosome regions displaying a high density of tumor suppressor genes [14]. A proteogenomic study of HGS-OvCas showed that the correlation between copy number alterations and consequent corresponding changes at protein is high and corresponds to >70%; the correspondence between mRNA expression data and proteomic data is even higher, corresponding to 90% [15]. The proteomic analysis confirmed the abnormalities of some signaling pathways showed by the genomic analysis and indicated also the functional convergence of some of these pathways on few biologic activities, such as increased invasiveness and motility, a property clearly associated with short overall survival [15]. Finally, the proteomic analysis provided also complementary informations for the definition of some HGS-OvCa subsets, such as those associated with HRD (Homologous Recombination Deficiency) and characterized by some specific acetylation events, a finding with potential therapeutic implications [15].

The analysis of the haploinsufficiency network in HGSOC has led to the discovery that autophagy in the most significantly disrupted biologic pathway by coincident gene deletions in these tumors. Targeting autophagy and proteostasis pathways abolishes the growth of HGS-OvCa cells [16]. In line with these findings, genes involved in autophagy, such as BENC1 and LC3 are monoallelically deleted in 94% of HGSOCs; the deletion of BECN1 and LC3 in ovarian cancer cell lines not derived from HGS-OvCa induces a considerable sensitization to autophagy inhibitors [16].

Recurrent recombinant events specific to ovarian cancer seem to be not frequent. In this context, Salzman and coworkers reported in 2011 the frequent (about 15%) occurrence of *ESRRA-C11orf20* fusion in serous ovarian carcinoma patients [17]. However, recent studies have failed to confirm a so high frequency of this chromosomal rearrangement and suggested that it is a rare event [18]. However, a recent study reported a recurrent fusion gene event in HGS-OvCa. In fact, Kannan and coworkers recently reported the inter-chromosomal fusion gene *CDKN2D-WDFY2* as a cancer-specific fusion gene recurrently (20%) occurring in HGSC [19]. The CDKN2D is a cell-cycle modulator of AKT interactions with its substrates [19]. Transfection experiments suggest that the fusion protein CDKN2D-WDFY2 induces the activation of the PI3K/AKT pathway [19].

A frequent abnormality observed in ovarian cancer is related to the *Let-7* microRNA and *HMGA2*. HMGA2 is a non-histone DNA binding factor that acts as an important regulator of cell growth, differentiation and apoptosis and is regulated by the *Let-7* micro RNA. *Let-7* microRNA is downregulated in ovarian cancer and determines an overexpression of *HMGA2*: *HMGA2* is overexpressed in 65% of ovarian cancers, and, particularly, in high-grade serous carcinomas [20]. The Let-7/HMGA2 dysregulation is a key factor in ovarian cancerogenesis in distinguishing type I from type II ovarian cancer, Let-7 downregulation being associated with less differentiated ovarian cancers [21]. *HMGA2* was frequently overexpressed also in ovarian carcinosarcomas [22].

2–4% of high-grade ovarian cancer patients display abnormalities of Anaplastic Lymphoma Kinase (ALK) expression due to either gene copy number gain or to the formation of the *ALK* fusion gene *ALK:FN1*; this finding indicates the existence of a potential therapeutic target in a minority of ovarian cancer patients [23].

Few studies have explored the problem of the comparative analysis of the mutational spectrum of ovarian cancers in primary and recurrent tumors. A study performed by Kim and coworkers on 46 epithelial ovarian cancer patients showed that somatic mutations did not differ between primary and recurrent tumors: every mutation present in the recurrent samples was detected in the corresponding primary sample [24]. In another study, Castellarin and coworkers have investigated the genome mutations of primary, first and second relapse tumor cells (derived from ascites) of few ovarian cancer patients and have observed that 89% of mutations found in relapse tumors were present in matched primary tumors, thus indicating that relapsing HGS-OvCas arise from pre-existence and persistence of tumor clones, in association with the accumulation of relatively few new mutations [25]. These results indicate also that antitumor chemotherapy was unable to kill the main tumor clones present in the primary tumor, as supported by the observation that not more than 10% of mutations were lost after this treatment [25]. Relapse-related mutations observed in the few patients included in this study occurred at the level of *PREX2* and *AP1b1* genes [25].

A study involving all genome sequencing of HGS-OvCas from chemoresistant patients was recently reported; this study englobed also the analysis of tumor samples before chemotherapy treatment [26]. The advantage of whole genome sequencing compared to whole exome sequencing used in the TCGA study [6], is related to the capacity of this technique to detect structural rearrangement occurring outside the coding areas of the genome, including translocations, rearrangements, gene fusions and breakages, as well as copy number alterations. This study confirmed both in chemotherapy-naïve and chemotherapy-resistant patients the occurrence of few point mutations [26]; furthermore, this study confirmed also that inactivating germline mutations in genes associated with homologous recombination repair, or *BRCA1* methylation, occur in about 50% of primary tumors and that *CCNE1* (cyclin E1) gain/amplification occurs in 19% of patients and are in large part exclusive of BRCA1/2 pathway disruption [26]. The analysis of chemoresistant patients showed that the acquired resistance to chemotherapy was associated with inactivating gene breakages at the level of the tumor suppressors *RB1, NF1, RAD51B* and *PTEN* and resistant/refractory tumors are frequently associated with *CCNE1* amplifications [26]. Interestingly, in some tumors developing chemoresistance, reversion in *BRCA1* or *BRCA2* mutations or loss of *BRCA1* hypermethylation was observed [26]. Finally, the gene encoding the membrane transporter, multi-drug resistant protein 1 (MDR1) was upregulated, because of promoter translocation or fusion events, in about 8% of recurrent tumors [26]. The analysis of mutational signatures showed that much of patients pertains to the age and BRCA-associated [26] signatures, while a minority of patients display APOBEC or mismatch signatures. There was an association between the Age signature and age of patients at diagnosis and *CCNE1* amplification, while the BRCA mutational signature was dominant in all samples with germline or somatic inactivation of *BRCA1* or *BRCA2* and was associated with a better response to therapy [26].

A recent integrated analysis of point mutation, copy number and rearrangement features has shown that HGS-OvCas can be subdivided into different prognostically relevant mutational subgroups [27]. Two large subgroups are the most frequent: the homologous-recombination-deficient (HRD) mutation signature (53%) and the foldback inversion (FBI) structural variation signature (41%) [27]. A prominent association was found between FBI and poor response to platinum-based chemotherapy [27]. FBI-dominated tumors are mutually exclusive to HRD tumors and display few point mutational events and a high number of amplifications co-localized with foldback rearrangements typical of breakage-fusion-bridge processes [27]. Importantly, *CCNE1* amplification, as well as PTEN focal copy number deletion, are frequent in FBI tumors [27]. The HRD group comprises tumors bearing BRCA1 somatic or germline mutations, methylation of the BRCA1 promoter, *BRCA2* somatic or germline mutations, and point mutations or gene breakages in various HR-related genes; these tumors displayed *MECOM* (3q26.2), *MYC* (8q24.21) and *CCND1* (11q13.3) amplification and *RB1* focal copy number deletion [27].

### 2.2. TP53 Mutations in HGSOCs

The Cancer Atlas Genome has shown that 96% of HGS-OvCas have *TP53* somatic mutations, thus suggesting that mutation of this gene is a virtually path gnomic, defining feature of this cancer. Interestingly, the histologic analysis of the few TP53 WT HGS-OvCas showed that the large majority of these tumors have morphologic features not typical of HGS-OvCas or are not pure HGS-OvCas [28]; furthermore, the analysis of the molecular features indicate that these tumors are not HGS-OvCas [28]. Therefore, the conclusion was that 100% of HGS-OvCas are *TP53*-mutated [28].

Recent studies have in part clarified the essential role of TP53 mutation in ovarian cancer development. The large majority (about 80%) of *TP53* mutations occur at the level of the DNA binding domain (Figure 1). The large majority of *TP53* mutations occurring in HGS-OvCas are missense mutations (70.4%), followed by frameshift mutations (about 12%) and splice mutations (5.1%) (Figure 1) [29]. P53 acts as a homotetrameric transcription factor and its mutations determine three different phenotypes: loss-of-function; dominant-negative and gain-of-function. The large majority of these *TP53* mutants exhibit a loss of- the function of normal TP53. The mutated TP53 proteins tend to accumulate in tumor cells because they have lost the capacity to interact with their physiologic inhibitors MDM2 and MDMX and, thus, are not degraded by proteasome. Missense *TP53* mutants possess also an inhibitory activity on WT *TP53* because form a heterotetramer with WT TP53 [29]. Finally, some missense *TP53* mutants, those commonly called hot-spot mutants, R175H, G245S, R248W, R249S, R273C, R273H or R282W, scarcely retains the normal TP53 activity (0–20%) and often acquire new functions (gain-of-function) [29].

The *TP53* mutation is the first genetic somatic mutation occurring during HGS-OvCa development, as supported by the observation that *TP53* mutations are observed in early tumor precursor lesions [30].

Stabilization of TP53 by missense mutation, but not its loss, induces the survival of fallopian tube non-ciliated epithelial cells and cell-cell aggregation under anchorage-independent growth condition [31]. In addition to anchorage-independence, *TP53*-mutated cells acquire mesothelial intercalation capacity through a mechanism involving mesenchymal transition and matrix production [31]. Most of *TP53* missense mutations, such as R175H, R248Q and R273H, express high amounts of shorter p53 isoforms, exhibiting gain of function properties attributed to the mutant protein and required for its oncogenic activity [32]. Finally, a recent study analyzed HGS-OvCas for *TP53* mutation and expression [33]. 62% of these tumors displayed missense mutations, associated with high TP protein levels; in contrast, most of non-missense mutations were associated with low TP53 levels [33].

As stated above, TP53 missense mutations result in a single amino acid substitution in the TP53 protein and are the most common TP53 mutations in ovarian cancer. Some of these mutations result in gain-of-function pTP53 biologic activity and are associated with increased TP53 protein expression [34,35]. Two studies analyzed *TP53* mutant HGS-OvCa TCGA cases subdivided by gain- or loss-of-function evaluation. One study failed to show any significant difference in OS and PFS between these two groups of patients [34]. In contrast, the second study showed decreased PFS among patients with gain-of-function *TP53* mutation [35].

Seagle and coworkers analyzed the impact of the location of *TP53* mutation within the TP53 protein on overall survival: patients with missense mutations located in the minor groove of the DNA binding domain of TP53 have a clearly better OS than those with missense mutations at the level of the major groove of the DNA binding domain [36]. Patients with mutations at the level of the SLH stabilizer have a negative prognosis, while those with mutations at the level of the Zn binder have a clearly better OS [36].

### 2.3. HRD in HGS-OvCa

As mentioned above, about 50% of HGS-OvCas display a homologous recombination deficiency caused by BRAC 1–2 mutation/repression of expression and by abnormalities of other genes of the DNA repair machinery. These alterations are collectively described as having a “BRCAness” phenotype, because of the genomic instability associated with BRCA dysfunction. The study of these abnormalities promoted the development of a new therapeutic approach based on targeting of homologous recombination deficiency.

It is important to note that in addition to germline and somatic mutations, and to promoter hypermethylation, *BRCA1* and *BRCA2* genes frequently display loss of heterozygosity (LOH). *BRCA1* alterations, comprising germline/somatic mutations, aberrant promoter methylation and/or allelic loss are found in about 78% of HGS-OvCas; interestingly, the frequency of *BRCA1* LOH was higher in tumors with *BRCA1* germline (91%) than in tumors from non-carriers (72%) [37]. Kanchi and coworkers estimated the frequency of LOH in 100% and 76% of cases with germline *BRCA1* and *BRCA2*, respectively [38]. Since not all tumors from individuals with germline *BRCA1* or *BRCA2* mutations have locus-specific LOH, it seemed important to evaluate the possible impact of the LOH status on platinum or PARP inhibitors treatment. Thus, Maxwell and coworkers have explored this issue [39]. All the *BRCA1* germline mutation-associated tumors without LOH (corresponding to 7% of these tumors) had HRD and BRCA mutational signature scores well below the mean for tumors demonstrating locus-specific LOH [39]. The same applies to *BRCA2* germline mutation-associated tumors [39]. Importantly, both *BRCA1* and *BRCA2* HGS-OvCas without locus-specific LOH treated with adjuvant platinum-based chemotherapy displayed lower overall survival than tumors with locus-specific LOH, at rates similar to sporadic HGS-OvCas [39].

The standard of care implies primary surgical cytoreduction, followed by platinum-based chemotherapy. This treatment developed in the 1990s allowed to improve the survival of patients with advanced HGS-OvCa. The good sensitivity of HGS-OvCas to platinum is thought to be related to an underlying defect in homologous recombination-mediated DNA repair. Given this background, it seemed logical to evaluate poly-ribose polymerase (PARP) inhibitors in BRCA-mutated HGS-OvCas: in fact, PARP inhibitors, such as Olaparib, causes in BRCA-deficient cells an increase in DNA aberrations, a part of which cannot be repaired due to homologous recombination deficiency, resulting in cell death via synthetic lethality [40]. The synthetic lethality observed in these tumor cells is due to the combination of the effects of the intrinsic homologous recombination repair defect of BRCA-deficient ovarian tumor cells. The PARP inhibitor Olaparib was approved in 2014 for the treatment of patients with *BRCA* mutations and recurrent disease [41]. Importantly, in spite the selective activity of Olaparib against BRCA-deficient ovarian cancers, more patients responded to the PARP inhibitor therapy than those patients with mutations [42]. In line with these findings, in the multinational, phase 3 NOVA trial in HGSOC patients with platinum-sensitive, recurrent ovarian cancer, Niraparib, a highly-selective, potent PARP1 and PARP2 inhibitor, significantly prolonged median progression-free survival, irrespective of the presence or absence of a germline BRCA mutation and irrespective also of the presence or absence of homologous recombination repair deficiency [43].

Olaparib was used also for the maintenance therapy of HGS-OvCa patients. In fact, it was shown that Olaparib maintenance treatment elicited a significant improvement of PFS, with no negative effect on the quality of life, in patients with platinum-sensitive relapsed ovarian cancer and a *BRCA 1–2* mutation [44]. The analysis of overall survival of patients who have participated to the initial studies with Olaparib showed no benefit in the whole patient population, but a significant improvement in overall survival in patients with *BRCA* mutations (34.9 months in Olaparib arm, compared to 30.2 months in the placebo group) [45]. Thus, the conclusion from these studies is that, although Olaparib is of benefit for treatment of *BRCA*-mutant, this benefit in terms of overall survival is very limited.

Finally, recently it was evaluated the clinical activity of Rucaparib, another PARP inhibitor. In patients with *BRCA* mutant or *BRCA* wild-type and *BRCA1* loss-of-heterozigosity (LOH), platinum-sensitive HGSOCs treated with Rucaparib, progression-free survival was longer than in patients with *BRCA* wild-type LOH low carcinomas [46]. Finally, another recent study provided evidence that Rucaparib significantly improved PFS in patients with platinum-sensitive ovarian cancer who had achieved a response to platinum-based chemotherapy, thus indicating that PARP inhibitors could be used in the maintenance treatment of HGS-OvCa [47].

Patients with epithelial ovarian cancer bearing *BRCA1* or *BRCA2* mutations have a better short-term survival (5 years) than noncarriers; however, this survival time was lost over time and after 5 years, *BRCA1* carriers had a higher risk of dying than noncarriers, while a survival advantage longer persisted among BRCA2 patients [48].

BRCA2 is a very large protein and contains a RAD51 binding domain and a DNA binding domain [49]. Only *BRCA2* carriers with truncating mutations at the level of the RAD15 binding domain have 5-year prolonged PFS, while *BRCA2* carriers with mutations located in other domains do not have prolonged 5-year PFS [49].

### 2.4. Clonal Evolution of HGS-OvCa: Spatial and Temporal Heterogeneity

The peritoneal cavity does not represent a distinct physical barrier and offers to ovarian cancer cells the opportunity for cancer metastasis, resulting in early widespread disease diffusion at distal peritoneal sites. The common view is that the majority of disease spread in HGS-OvCa is related to a cell mixing derived from the invasion of an anatomical cavity by multiple cellular tumoral elements.

An additional important element of the genomic complexity of HGS-OvCas is related to the molecular diversity present at the level of tumor sites of each individual patient, reflecting the clonal evolution occurring during tumor progression. Thus, Bashashati and coworkers [50] have examined 31 spatially and temporally separated specimens derived from 6 patients with HGS-OvCa and observed widespread genomic variability (mutation, copy number alterations, and key driver gene alterations) within tumors from the same patient [50]. Importantly, molecular alterations in driver genes such as *CTNNB1, NF1, PDGFR, PIK3CA, SH3GL1* and *RBM15* were found to have a subclonal distribution, thus indicating that they have been acquired during tumor evolution. These subclonal mutations greatly contribute to the final tumor heterogeneity [50]. Hoogstraat and coworkers have confirmed the existence of tumor heterogeneity in treatment-naïve HGS-OvCas. Particularly, they investigated the mutational profile and copy number alterations observed in 15 spatially separated samples from different tumor sites derived from two HGS-OvCa patients [51]. One of these two cases displayed a great heterogeneity at the level of mutational profile, copy number alteration, gene expression profile and key cancer pathway activation between samples from the primary tumor site and those derived from peritoneal and omental metastases [51]. Interestingly, one of the two patients displayed two different *TP53* mutations in ovarian tumor and omentum/peritoneum metastases, respectively [51]. The existence of two *TP53* mutations in different tumor sites was recently reported also in another study: one *TP53* mutation was exclusive of the primary tumor mass and the other one was observed exclusively in ascite tumor cells, peritoneal tumor masses and a lymph node metastasis [52]. This peculiar mutually exclusive pattern of distribution of two different TP53 mutations in different tumor tissues is compatible with the generation in the same patients of two different HGS-OvCas, one issued from a precancerous lesion in the fallopian tube and the other one from the ovaries.

From these studies, it emerges the picture of HGS-OvCa as a tumor disease, characterized by multiple populations of genetically and phenotypically distinct subclones evolving from an ancestral clone, following patient-specific pathways of tumor branching. In this context, Schwarz and coworkers through the analysis of spatial and temporal heterogeneity of tumors derived from 14 HGS-OvCa patients reached the important conclusion that the emergence of subclonal tumor populations in these patients was associated with the development of resistant disease: particularly, the subclonal tumor populations, present in pre-treatment biopsies of HGS-OvCa can undergo expansion following chemotherapy and cause clinical relapse [53]. Other studies have confirmed the occurrence of intra-tumor heterogeneity of HGS-OvCa, involving also significant changes in copy number alterations occurring in primary versus metastatic lesions; these copy number variations preferentially involve some pathways, including JAK/STAT and cytokine signaling pathways [54].

Choi and coworkers have recently reported the multiregional analysis of intraovarian and extraovarian tumor lesions from HGS-OvCa patients and classified the observed genetic alterations into “common”, “shared” and “private”. In cancer-related genes, six common, eight shared and 24 private mutations were observed [55]. Interestingly, region-specific chromosomal amplifications and deletions involving *BRCA1, PIK3CA* and *RB1* were identified [55]. In spite the consistent intra-tumor heterogeneity, the somatic mutations, copy number alterations and DNA methylation in both cancer-related and common genes are highly conserved in tumor ascetic cells, suggesting a multiregion origin of ascetic cells [55]. In conclusion, the genetic intra-tumor heterogeneity is a driving force in ovarian cancer genomes for the emergence of new subclones with metastatic potential.

McPherson and coworkers have recently reported the results of an interesting study aiming to evaluate what proportion of spreading of HGS-OvCas in the peritoneum arises from monoclonal seeding and expansion or, alternatively, from extensive cellular mixing of multiple polyclonal populations [56]. The results of this analysis showed that most peritoneal sites are clonally pure or composed of clones from a single phylogenetic clone. Five patients exhibited monoclonal and unidirectional seeding from the ovary to intraperitoneal sites, while two patients displayed polyclonal spread and reseeding [56]. These observations support the existence of two modes of intraperitoneal spread, operating in ovarian cancer clonal dissemination [56].

The aggressiveness of HGS-OvCa is based on its capacity of rapid dissemination to the peritoneum, omentum and organs located in the peritoneal cavity. Intraperitoneal seeding is considered the primary route of HGS-OvCa dissemination. However, the presence of circulating tumor cells and abundant tumor cells in the omental vasculature has led to hypothesize alternative routes of metastasis of this tumor. Using parabiosis models, Pradeep and coworkers have provided evidence that circulating tumor cells implant and grow in the omentum preferentially and, subsequently, spread to other peritoneal surfaces [57]. Elevated levels of Human Epidermal Growth Factor Receptor 3 (ErbB3/HER3) in ovarian cancer cells and Neuregulin 1 (NRG1) in the omentum allowed for tumor cell localization and growth in the omentum [57].

### 2.5. Familial HGS-OvCa

As mentioned above, family history of ovarian cancer in first degree relatives increases the risk for epithelial ovarian cancer; studies on these families have led to the identification of moderate-to-high penetrance genes favoring ovarian cancer development: *BRCA1*, *BRCA2* and the DNA mismatch repair gene *RAD51C*. It was estimated that germline *BRCA1* and *BRCA2* mutations contribute to the development of 10–20% of EOCs. Data from the Breast Cancer Linkage Consortium suggest the risk of epithelial ovarian cancer through age 70 years is up to 44% in BRCA1 families and is up to 27% in BRCA2 families. Women with mutations in the DNA repair genes *BRIP1, RAD1C* and *RAD1D* have estimated lifetime risks of 5.8%, 5.2% and 12%, respectively [58,59]. A recent study carried out on many ovarian cancer patients showed that 3.6% displayed germline *BRCA1* mutations, 3.3% germline BRCA2 mutations and 2.9% germline *RAD15C* mutations [3]. These patients with germline mutations of these genes have HGS-OvCas, an earlier diagnosis age and an ovarian cancer family history [60]. Recently, Gabai-Kapara and coworkers have performed a population-based screening for ovarian cancer risk due to BRCA1 and BRCA2 in the Ashkenazi Jewish population of Israel; it was estimated that in this population 40% of ovarian cancers are due to *BRCA1* and *BRCA2* mutations. The results of this interesting study showed that cumulative risk of developing ovarian cancer by age 60 and 80 respectively, were 0.60 and 0.83 from *BRCA1* and 0.33 and 0.72 for *BRCA2* carriers [61]. It was estimated that approximately 20% of the familial component of epithelial ovarian cancer risk is attributable to high-to-intermediate risk genes. An unknown, larger fraction is due to more common, lower-risk, gene variation.

As above indicated, only a part of the familial aggregation of Epithelial Ovarian Cancer can be explained by high-penetrance alleles of *BRCA1* and *BRCA2*. Thus, additional susceptibility loci for Epithelial Ovarian Cancer: variant at 1p36 (nearest gene, *WNT4*), 4q26 (*SYNPO2*), 9q34.2 (*ATAD5*), 1p34.3 (*RSPO1*) and 6q22.1 (*GPX6*) [62]. According to these findings it was proposed a model of EOC susceptibility, implying large differences in absolute Epithelial Ovarian Cancer risk between individual carrying several risk-associated alleles and individually carrying only few alleles for Epithelial Ovarian Cancer susceptibility in *BRCA1* and *BRCA2* mutation carriers [62]. This hypothesis was directly confirmed by a recent study evaluating the polygenic risk scores for women carrying a pathogenic mutation in the high-risk *BRCA1* or *BRCA2* genes [63]. In fact, combining the various risk scores, a polygenic predictive risk score was formulated, indicating that the additional ovarian cancer, single nucleotide polymorphism associated with the risk of development of ovarian cancer considerably impact the risk of developing ovarian cancer in *BRCA 1–2* carriers: patients at the low percentile of polygenic risk may have a 6% risk by age 80 years, while patients at a high percentile risk have a 19% risk of developing ovarian cancer by age 80 years [64]. Six additional susceptibility loci for HGS-OvCa were recently reported, 2q28, 4q32.3, 8q21.11, 10q24.33, 18q11.2 and 22q12.1 [42]. Interestingly, in this study, the *OBFC1* was identified as a susceptibility gene for low-grade ovarian cancers [64].

The ovarian cancer susceptibility loci are maintained in ethnological populations of different origin. Thus, Chen et al. performed a genome-wide association study of Han Chinese women and showed that four loci previously reported in European populations (3q25, 17q12, 17q21, 19p13.11) influence ovarian cancer risk [65].

Women with germline *BRCA1* or *BRCA2* mutations are recommended to undergo prophylactic bilateral salpingo-oophorectomy (RRSO) to reduce their risk of developing ovarian cancer (and breast cancer), usually by age 40 or after the completion of childbearing [44]. In *BRCA1–BRCA2* carriers RRSO reduces the risk of ovarian cancer by 85–90% and the risk of breast cancer by about 50% [66]. The pathological specimens obtained in these patients have given the unique opportunity to examine these tissues for occult cancers and have shown that in patients who have developed cancers, most lesions were identified in the fallopian tube [67].

Women with germline *BRCA1* or *BRCA2* mutations are recommended to undergo salpingo-oophorectomy bilateral (prophylactic oophorectomy, RRSO) to reduce their risk of developing ovarian cancer (and breast cancer), usually by age 40 or after the completion of child bearing [66]. In *BRCA1–BRCA2* carriers RRSO reduces the risk of ovarian cancer by 85–90% and the risk of breast cancer by about 50% [66]. The pathological specimens obtained in these patients after surgical debulking have given the unique opportunity to examine these tissues for occult cancers and have shown that in patients who have developed cancers, most lesions were identified in the fallopian tube [67].

### 2.6. Gene Expression Profiling Studies of Serous Ovarian Cancer

Gene expression profiling studies revealed an unappreciated molecular diversity within high-grade serous ovarian cancer by delineating six distinct molecular subtypes: (a) C1 typically included frequent high-grade ovarian cancers, defined by reactive stroma signature, associated at pathological level with extensive desmoplasia: markers of activated myofibroblasts were typically expressed in association with gene ontology groups defining extracellular matrix production and remodeling, cell adhesion, cell signaling and angiogenesis; (b) C2, typically included common high-grade ovarian cancers, characterized by the expression of genes and signaling pathways associated with immune cells: at the histological level these tumors were characterized by a high intra-tumor and intra-stroma infiltrate of CD3^+^ lymphocytes; (c) C3 included low malignant potential ovarian cancers and invasive tumors with an associated LMP component and were characterized by low expression of proliferation markers, overexpression of MAPK pathway gene, associated with mutations in MAPK pathway members *KRAS* and *BRAF*; (d) C4 included high-grade ovarian cancers, characterized by a low expression of stromal genes and by a gene expression profile similar to C2 and by high expression of CA-125; (e) C5 was represented by a novel high-grade subtype defined by genes expressed in mesenchymal development: in this subtype overexpression of some homeobox genes, high-mobility group members, WNT/beta-catenin and cadherin signaling pathways (including N-cadherin and P-cadherin), was observed; (f) C6 subtype is mainly represented by low-grade endometrioid tumors, characterized by overexpression of transcriptional targets of the beta-catenin/LEF/TCF complex and by a consistent deregulation of the WNT/beta-catenin signaling pathway [68]. The univariate survival analysis showed that: C3 and C6 subtypes display the best survival in line with the presence of LMP and low-grade tumors in these subgroups; C1 subtype had the poorest survival among the high-grade groups; C2 and C4 tumors had better overall survival than C1 tumors; C5 mesenchymal displayed a reduced overall survival compared to subtypes C2 and C4 [68]. More recently, the C5 subtype was better characterized from a molecular point of view: in fact, in these tumors a specific activation of a pathway involving MYCN, LIN28B, Let-7 and HGMA2 was observed; this conclusion was supported by the observation that MYCN was amplified and overexpressed, the MYCN targets including the repressor LIN28B were induced, Let-7 expression was downmodulated and HMGA2 was amplified and overexpressed [69].

Other gene expression profiling studies were based on the idea to identify microarray platforms to be used to detect gene whose expression correlated with response or resistance to standard treatment of ovarian cancer. In this context, a first study carried out by Dressman and his coworkers identified some genes whose expression was correlated with response or resistance to treatment: thus, the expression of genes involved in cell proliferation/cell growth is associated with response to treatment, a finding well in line with the mechanism of action of cytotoxic chemotherapic agents such as cisplatin and paclitaxel; on the other hand, a deregulation of SRC, MYC and E2F3 pathways correlated with a poor response to standard chemotherapeutic treatment [70].

A similar approach was used by Crijns and coworkers that have tried to identify gene expression profiles associated with different survival rates. This study was based on the analysis of patients with advanced-stage serous carcinoma and has led to the identification of a high-risk and low-risk group of patients. Genes involved in some important functional pathways, such as cell cycle, Wnt, JAK-STAT and MAPK, were defined as having a major role in ovarian cancer development and prognosis [71]. In addition to these pathways, this study identified some transcription factors linked to ovarian cancer prognosis, such as p53, c-Ets-1, E2F transcription factors, C/EBP family and CREB [71]. In a subsequent study, Yoshihara et al. identified a 126-gene expression signature that allowed to separate ovarian cancer patients with advanced disease into two risk groups and to predict their survival. This analysis allowed the identification of a peculiar set of genes associated with risk. In fact, it was observed that a significant reduction in expression of immune response-related genes, particularly on the antigen presentation pathway, clearly associates with high-risk ovarian cancers [72]. These observations suggest that defects in HLA class I antigen presentation machinery would decrease recruitment of tumor-infiltrating lymphocytes at the level of the tumor, leading to an uncontrolled tumor growth due to a reduction in anti-tumor activity [72]. In line with this interpretation previous findings have shown that the presence of tumor-infiltrating lymphocytes within the tumor was associated with long survival in ovarian cancer patients.

Another study has tested the hypothesis whether ovarian cancer patients with poor vs. favorable outcomes, following platinum-based chemotherapy, have tumors that have differential expression of genes involved in repair of platinum-induced DNA damage. To this end, 151 DNA repair genes have been studied in ovarian cancer patients: a high score of expression of genes involved in platinum-specific DNA repair pathways was associated with improved overall survival, while a low score of expression of these genes was associated with worse overall survival [73].

A detailed analysis of the gene profiling studies on high-grade ovarian cancers allowed to identify and characterize some genes whose expression is directly related to patient’s survival. This is the case of a study leading to the: identification of *MAGP2* (Microfibril-Associated Glycoprotein 2) as a gene whose expression is associated with poor prognosis [74]. MAGP2 encoded for a proangiogenic protein stimulating tumor survival and promoting endothelial cell motility and survival through the α_v_β_3_ integrin pathway [74]. In histopathological specimens MAGP2 expression correlated with microvessel density, suggesting a proangiogenic effect of this protein in vivo [74].

As stated above, although most of women with HGS-OvCa have a good response to standard chemotherapy treatment, about 20 to 30% of patients relapse within 6 months. A comparative analysis of copy number gene abnormalities observed in early relapse compared to late relapse patients identified *CCNE1* amplification as the dominant chromosomal abnormality associated with treatment failure [6]. On the other hand, the analysis of paired tumor samples collected before and after chemotherapy treatment has offered the opportunity to identify some biochemical determinants that could be related to the development of platinum resistance. This analysis provided evidence that HDAC4 overexpression frequently occurs in platinum resistant cells, that favors STAT1 activation following platinum exposure. In platinum-sensitive cells STAT1 is acetylated and its activation is prevented: HDAC4 silencing increased acetyl-STAT1 levels, prevented platinum-induced STAT1-activation and restored sensitivity to platinum [75]. Other studies have suggested a potentially important role for AKT in mediating platinum resistance. In fact, it was shown that platinum exposure induces an AKT-dependent, pro-survival, DNA damage response in clinically platinum-resistant, but not platinum-sensitive cells: in platinum-resistant cells AKT relocates to the nucleus where is phosphorylated by DNA-PK and this activation inhibits platinum-mediated apoptosis [76].

More recent gene expression studies have attempted to define a possible link between ovarian cancer expression subtypes and prognosis. The Cancer Genome Atlas (TCGA) describes four overlapping subtypes and titled these as immunoreactive, differentiated, proliferative and mesenchymal, but was unable to identify any significant difference in these subtypes at the level of clinical outcome [6]. A recent meta-analysis of the TCGA data by Verhaak and coworkers has led to the development of subtype and survival gene expression signatures which, combined, provided a new prognostic classification of HGS-OvCa, called “Classification of Ovarian Cancer” CLOVAR [77]. This analysis allowed to show that the “immunoreactive” group had the best prognosis, whereas the “mesenchymal” subtype was associated with poor outcome [77]. Particularly, the mesenchymal subtype, accounting for about 23% of all cases, was associated with a median survival of 23 months and a platinum resistance rate of 63%, compared to a median survival of 46 months and platinum resistance rate of 23% [77]. These observations were confirmed in a more recent study by Konecny and coworkers supporting the prognostic relevance of molecular subtypes identified by TCGA network: in fact, survival was best for the immunoreactive-like subtype and significantly worse for the proliferative or mesenchymal-like subtypes [78]. Zhang and coworkers have integrated genomic, epigenomic nd transcriptomic features of HGS-OvCas and, using this approach, have identified seven patient groups that significantly differed in median survival time: particularly, patients pertaining to groups 2, 4 and 5 (32% of total patients) have a poor prognosis, with a median survival time of <3 years; patients in subtypes 3 and 7 (about 42% of total patients) have a better prognosis, with a median survival time >4.5 years; subtypes 1 and 6 have an intermediate prognosis [79]. Subtype 2 was characterized by upregulation of many genes involved in tumorigenesis, such as genes involved in cell adhesion, angiogenesis, TGF-beta binding and positive regulation of cell proliferation; clusters 4 and 5 are characterized by the overexpression of genes involved in proliferation circuits pertaining to the mTOR, ERBB, MAPK signaling pathways, due to gene amplifications of genes such as *KRAS*, *SRC*, *PLCG1*, *AKT* and *E2F1* [79]. A large scale meta-analysis of epithelial ovarian cancer microarray datasets has allowed Tan and coworkers to identify five, biologically distinct subgroups: Epithelial A, Epithelial B, Mesenchymal, Stem-like A and Stem-like B. Epi A, Epi B and Stem B subtypes have a better prognosis, while Mes and Stem A tumors were linked to poorer outcomes [80]. The Mes group included more advanced and metastasized ovarian cancers [80]. A more recent study has in part clarified the reasons of the negative prognosis of the Stem A subtype. This ovarian cancer subtype expresses genes regulating stemness. Particularly, it was shown that the overexpression of a receptor of the Wnt signaling pathway, the Frizzled family receptor 2 (FZD7) in the Stem A subtype: in vitro experiments support a role for FZD7 in driving the aggressiveness of Stem A subtype ovarian cancer cells [81]. Riester and coworkers have developed and validated a two-gene expression signature and, using this strategy, have identified a survival signature that allowed to stratify HGS-OvCa patients into high- and low-risk groups [82]. The signature seems to distinguish better the high- and low-risk patients than the TCGA signature [44]. The expression of few genes, including *POSTN*, *CXCL4*, *FAP*, *NUAK1*, *PTCH1*, *TGFBR2* and the phosphorylated Smad 2/3 allowed to distinguish in the large majority of cases (>90%) the high-risk from the low-risk group [82]. Another recent study has provided evidence that a 10-gene signature, including genes encoding extracellular matrix proteins involved in collagen remodeling, identifies HGS-OvCa patients with poor overall survival and with high metastatic potential [83]. Interestingly, the expression of COL11A1, one of the gene present in this signature, continuously increases during ovarian cancer disease progression [83].

Thus, most studies converge on the identification of four molecular subtypes of HGS-OvCa: C1 (mesenchymal) subtype, with extensive myofibroblast infiltration (desmoplasia), an epithelial-mesenchymal gene expression signature, associated with primary treatment failure and poor survival; C2 (immunoreactive) subtype HGSOC, with extensive intra-tumoral T lymphocyte infiltration and a more favorable prognosis; C4 (differentiated) subtype, with a gene signature resembling serous borderline tumors, associated with an intermediate prognosis; C5 (proliferative) subtype, with low expression of differentiation markers, limited inflammatory infiltration, activation of a signalling pathway involving several oncogenic and stem cell factors, associated with a poor prognosis (Table 3). The introduction of this gene expression profiling into the clinical routine is difficult, for its technical complexity and for the consistent cost. Thus, Leong et al. have recently proposed a simplified screening system based on gene signature involving only 39 genes differentially expressed among the different subgroups, able to distinguish the four main HGS-OvCa subtypes [84]. The definition of C1, C2 and C4 subgroups using this approach was clear and unambiguous, while the C5 subgroup displayed a consistent heterogeneity [84].

Recently, through the analysis of a large public cohort of data and through the analysis of 381 HGS-OvCa cases of the Mayo Clinic it was proposed a modification of the four subtypes classification into a 5 subtypes classification (Table 3) [85]. This new classification was based on the discovery of a consistent heterogeneity of the C4, differentiated subtype, now subdivided into two new types, one of which (anti-mesenchymal) displayed downregulation of genes that are typically upregulated in the mesenchymal subtype, while the other (differentiated) corresponded to the previously reported differentiated subtype (Table 3) [85]. The various molecular subtypes were associated with differences in overall survival and with surgical outcome: thus, the rate of complete surgical debulking was lower in mesenchymal subtype (16%) than in other tumor subtypes (>28%) (Table 3) [85].

This tumor classification of HGS-OvCa based on gene expression parameters stimulated the development of a new histopathological classification of these tumors, based on the identification of four tumor histiotypes, largely overlapping with the gene expression subtypes: mesenchymal transition, defined by a desmoplastic reaction; immune reactive, defined by lymphocyte infiltrating the tumor; solid and proliferative, defined by a solid growth pattern; papillo-glandular, defined by a papillary architecture [86].

Recent study attempted to identify gene signatures predictive of high-risk and low-risk HGS-OvCas [87,88]. In this context, particularly interesting were the results of a study carried out by Matondo and coworkers, reporting the identification of a prognostic 97 chemoresponse gene signature in HGS-OvCa patients [88]. This 97-gene signature was identified as an independent predictor of patient survival in association with other clinic-pathological factors in univariate and multivariate analyses [88]. This signature could predict patients who would attain complete or partial remission or no-response to first-line chemotherapy [88]. Interestingly, pathway analysis showed that the signature was regulated by *TP53* and *HIF-1α* and *HIF-1α*-regulated genes, which were particularly overexpressed in non-responder and partial remission patients than in complete remission patients [88].

The clinical relevance of the HGS-OvCa classification is strongly supported by some recent studies. Thus, a recent study based on the analysis of a very large number of ovarian cancer patients (more than 5500 patients with various types of ovarian cancer) provided clear evidence that HGSOC patients displaying a high number of intra-tumor CD8^+^ T lymphocytes have a longer overall survival [89]. Particularly, median overall survival was 2.8 years for patients with no CD8^+^ T lymphocytes and 3.0 years, 3.8 years, and 5.1 years for patients for patients with low, moderate, or high levels of CD8^+^ T lymphocytes, respectively. Importantly, in HGS-OvCa patients, CD8^+^ T lymphocytes were favorable regardless of extent of residual disease following cytoreduction, known standard treatment and germline BRCA1 pathogenic mutation [89].

The essential role of the immune mechanisms in the response of HGS-OvCa to therapy is supported also by an exceptional case of a patient treated with multiple chemotherapy regimens, who exhibited regression of some metastatic lesions, with concomitant progression of other lesions [90]. Genomic and immunologic studies it was provided evidence that the various metastases were molecularly heterogeneous, regressing and stable metastases were infiltrated by CD8^+^ and CD4^+^ T lymphocytes, while progressing metastases were characterized by immune cell exclusion [90]. Furthermore, at the level of regressing metastases, it was detected the presence of oligoclonal expansion of specific T cell subsets, reactive against tumor-specific neo-epitopes [90]. These findings support a key role of the immune response in mediating tumor progression and indicate that multiple distinct tumor immune microenvironments co-exist with a single patient and help to explain the heterogeneous fates of metastatic lesions [90].

Recently, it was analyzed the gene expression profile of primary peritoneal cancer and was compared to that observed in HGS-OvCa [91]. Peritoneal cancer is characterized by the diffuse involvement of abdominal peritoneal surfaces by carcinoma cells identical to those of HGS-OvCa, in the absence of a primary ovarian cancer; its incidence is markedly lower that HGS-OvCa and is observed not only in women with their ovaries in situ, but also in women carrying a germline BRCA mutation after prophylactic oophorectomy [91]. Interestingly, gene expression profiling classified a clearly higher proportion of primary peritoneal cancers as C1, mesenchymal subtype: 71% vs. 32% in HGS-OvCa [91].

### 2.7. Molecular Studies on Serous Ovarian Cancer Origin

Ovarian cancer has traditionally been thought to develop from ovarian surface epithelium or cortical inclusion cysts, but many recent studies suggest that a large part of HGS-OvCas originate from the epithelium of distal fallopian tube [3]. Thus, a peculiar lesion called serous tubal intra-epithelial carcinoma (STIC) is believed to be the precursor of most HGS-OvCas, as supported by their clonal relationship with established HGS-OvCas, based on both shared TP53 mutations, as well as on integrated molecular analyses, as it will be outlined below. STICs were originally identified in the fimbriated end of serially sectioned fallopian tubes, prophylactically removed from women at high-risk of developing ovarian cancer.

Next generation sequencing studies confirmed in most of patients the clonal mutational relationship between paired STIC and HGS-OvCa [92,93]. However, the comparative analysis of the spectrum of genetic abnormalities in STIC and corresponding HGSOC, performed in patients with sporadic, not familial HGS-OvCas, indicated that STICs in some patients are metastases rather than HGS-OvCa precursors [94].

Ducie and coworkers have performed an integrated genomic analysis of HGS-OvCas with or without detectable STICs at the histological analysis; these two types of HGS-OvCas are molecularly indistinguishable, thus suggesting that they have a common origin and are not molecularly different [95].

The role of STIC as a precursor of HGS-OvCa was further supported by a recent study reporting whole-exome sequence and copy number analysis of laser capture micro dissected fallopian tube lesions (p53 signatures, STICs and fallopian tube carcinomas) ovarian cancers and metastases [96]. In this study, among the various tubal lesions, it was reported the analysis of the so-called p53 signatures, identified as foci of strong TP53 immunostaining, initially described in tubal mucosa from BRCA-mutated women [96]. Most tumor-specific genetic alterations observed in ovarian cancers was observed also in STICs, including those affecting *TP53*, *BRCA1*, *BRCA2* or *PTEN* [72]. The reconstruction of the tumor evolutionary trees identified p53 signatures and STICs as precursors of HGS-OvCas and allowed to estimate in 7 years the time required for ovarian cancer generation from STICs [30].

The clonal relationship between STIC and HGS-OvCa was also supported by the analysis of cases displaying increased *CCNE1* copy number: this analysis showed that *CCNE1* copy number alterations were observed in 22% of STICs and 28% of HGS-OvCas; importantly, in paired analysis derived from the study of STIC and HGSOC in the same patients, a very good correlation was found between *CCNE1* amplification in STICs and HGS-OvCas, a finding compatible with the view that STIC is the precursor of HGS-OvCa [97].

## 3. Molecular Abnormalities of Ovarian Carcinosarcomas

Cracinosarcomas (CSs) of the female genital tract, also known as mixed malignant Mullerian tumors, are rare, highly aggressive tumors, characterized by a bilineage histology (a carcinoma and a sarcoma component). These tumors most commonly arise in the uterus, followed by the ovaries and are always characterized by the simultaneous presence of both sarcomatous and carcinomatous components. The survival for both early and late stage carcinosarcoma is inferior to serous tumors. A number of observational studies have indicated that ovarian cancinosarcomas follow a natural history distinct from that observed in other more common epithelial ovarian cancers [98]. Ovarian carcinosarcomas are aggressive tumors with an early dissemination [98]. The pathogenesis of CSs remains to be determined, but growing evidences suggest that both the histological components of these tumors originate from a common epithelial cell that undergoes sarcomatous dedifferentiation, rather than two independent progenitors. In support of this view, Ardighieri and coworkers have analyzed 16 pelvic CS patients and observed that 10 of these 16 patients (63%) displayed a concomitant serous intratubal carcinoma [99], thus providing evidence of a clonal relation between these two tumors [99]. According to these findings, it was concluded that CSs may have a metastatic nature and an extraovarian origin from STICs [99]. This conclusion is supported also by a recent molecular study reporting the mutational landscape of uterine and ovarian carcinosarcomas and demonstrating that carcinomatous and sarcomatous elements of these tumors derive from a common precursor having mutations typical of carcinomas [100]. In addition to mutations in cancer genes identified in uterine and ovarian carcinomas, such as *TP53*, *PI3KCA*, *PPP2R1A*, *KRAS*, *PTEN*, *CHD4* and *BCOR*, recurrent mutations in genes encoding histone *H2A* and *H2B*, as well as significant amplification of the segment of chromosome 6p harboring the histone gene cluster containing these genes [100]. Frequent deletions of the genes *TP53* and *MBD3* and frequent amplifications of chromosome segments containing the gens *PIK3CA*, *TERT*, and *MYC* were also observed [100]. Interestingly, stable transgenic expression of mutant *H2A* and *H2B* genes in a uterus carcinoma cell line, significantly increased expression of markers of epithelial-to-mesenchymal transition, thus suggesting a role of these gene in sarcomatous transformation [100]. Another recent study confirmed the occurrence of epithelial-to-mesenchymal transition gene signatures in a part of gynecological carcinosarcomas [101].

Another important molecular feature of gynecological carcinosarcomas consists of the frequent mutations in chromatin remodeling genes *ARID1A* and *ARID1B*, in histone methyltransferase *MLL3*, in histone deacetylase modifier *SPOP* and chromatin assembly factor *BAZ1A* [102]. *ARID1A/ARID1B* mutations are particularly frequent in ovarian carcinosarcomas, being much more frequent than in uterine carcinosarcomas [102].

In a transgenic mouse model of *KRAS* mutant and *p53* deletion within the ovarian surface epithelium, the formation of poorly differentiated ovarian carcinosarcomas was induced. These tumors were highly metastatic within the pelvic cavity, spreading to various abdominal organs [103].

## 4. Molecular Abnormalities of Low-Grade Serous Ovarian Carcinomas

**Figure 2:**
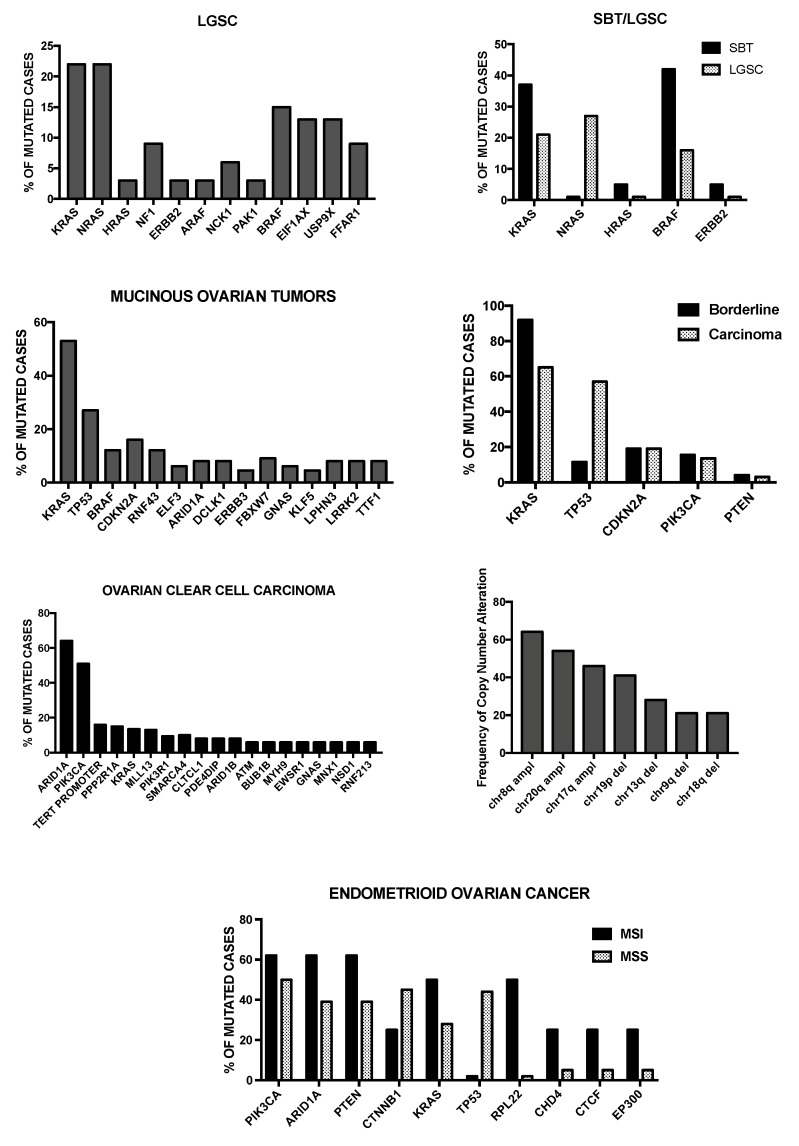
(**Top Panel, left**): Mutational spectrum observed in LGSOC patients; (**Top Panel, right**): comparative analysis of the frequency of the main mutations observed through paired comparison odf serous borderline tumors (SBT) and LGSOCs. The data reported in these two figures are from ref. [103]; (**Middle Panel, Top, left**): Recurrent mutations observed in mucinous cancers of the ovary; (**Middle Panel, Top, right**): comparative analysis of some recurrently mutated genes at the level of borderline precursor lesions and mucinous carcinomas; (**Middle Panel, Bottom, left**): Recurrent mutations observed in ovarian clear-cell carcinoma; (**Middle Panel, Bottom, right**): Recurrent copy number alterations observed in ovarian clear-cell carcinoma. The data shown in these two figures are reported in the references [117,118]; (**Bottom Panel**): Recurrent gene mutations observed in endometrioid ovarian cancers subdivided into microsatellite instable (MSI) and microsatellite stable (MSS) subgroups. The data shown in this figure are reported from ref. [27].

Low-grade serous ovarian carcinomas (LGS-OvCas) form a histological subtype of type I epithelial ovarian tumors, usually associated with an indolent clinical course and occurring at an age younger than HGSOCs. Although these tumors have a better prognosis than HGS-OvCas, patients with a higher stage of disease development have a poor overall survival due to the intrinsic chemoresistance of these tumors. Serous borderline tumors (SBTs) are considered as the non-invasive precursor lesions to LGS-OvCas; some SBTs may progress to LGS-OvCas [104]. An initial exome sequencing study of a limited number of LGS-OvCas showed the presence of *KRAS* and *BRAF*, but no other recurring mutations [5], while another study reported MAP2K1 mutation in 1/18 cases [105]. Patients not displaying RAS pathway mutations have a late stage of disease development and poor survival [106,107]. As mentioned above, LGS-OvCa typically have wild-type TP53. These tumors are thought to derive in a step-wise fashion from serous borderline tumors (SBT), present in >60% of LGS-OvCas. In contrast, HGS-OvCas with associated borderline histology are rare and may represent progression from *Ras*-mutated borderline or LGS-OvCas. In a recent study, Emmanuel and coworkers have screened a large number (>1200) of ovarian cancer patients and reported in 102 of these patients, serous carcinomas with adjacent borderline regions [108]. The identification of these patients with SBT-EOC allowed to perform an analysis of paired tumor samples derived from borderline regions: copy number alterations were virtually identical in these two regions, thus indicating a common origin [108]. Furthermore, the comparative analysis of tumors with only SBT component, with SBT-EOC and EOC only showed that: *BRAF, KRAS* and *ERBB2* mutations were more frequent in SBT only than in SBT-EOC tumors, and almost absent in EOCs; *NRAS* mutations were present only in a part (9%) of SBT-EOCs; *TP53* mutations were present in SBT-EOCs and EOCs (Figure 2) [108]. Often, peritoneal implants are associated with SBTs and until recently it was unclear whether they are derived from the primary ovarian tumor or arise independently in the peritoneum. A recent study provided clear evidence that the large majority of peritoneal implants harbor the identical *KRAS* or *BRAF* mutations identical to those present in the associated SBT, thus supporting the view that these peritoneal implants are derived from the primary ovarian tumors [109].

Hunter and coworkers have performed a genome-wide high-resolution genomic copy number analysis and mutation hotspot screening in 57 SBTs and 19 LGS-OvCas [110]. Copy number aberrations were observed in 61% of SBTs, compared to 100% of LGSOCs; oncogenic RAS/RAF/ERBB2 mutations were observed in 82% of SBTs, compared to 63% of LGSOCs; interestingly, *NRAS* mutations were detected only in LGS-OvCas [110] (Figure 2). Some copy number alterations, such as loss of 9p and homozygous deletions of *CDKN2A* locus are preferentially observed in LGS-OvCas. Exome sequencing studies identified *BRAF, KRAS, NRAS, USP9X* and *EIF1AX* as the genes most frequently mutated in these tumors [110]. RAS pathway mutations observed in LGS-OvCas are mutually exclusive; however, co-occurrence of NRAS and EIF1AX mutations was observed in a fraction of these tumors [111]. Co-expression of mutant NRAS and EIF1AX proteins was required to promote proliferation and clonogenic proliferation survival of LGS-OvCa cells [111].

## 5. Genetic Abnormalities of Mucinous, Endometrioid and Clear Cell Carcinoma

As mentioned above, mucinous tumors are the second most common form of epithelial ovarian tumors and account for about 35% of all epithelial ovarian tumors: 81% of these tumors are benign cystadenomas, 14% borderline and 5% malignant. Mucinous ovarian cancers are now classified as a type I tumor with an identifiable stepwise progression from a premalignant lesion, through non-invasive to invasive malignancy. Mucinous ovarian cancers scarcely respond to platinum treatment. A common genetic defect of ovarian mucinous tumors is represented by *KRAS* mutations, observed in 46–55% of mucinous cystadenomas, in 63% to 73% of borderline tumors and in 75–85% of mucinous carcinomas. These molecular evidences (i.e., presence of benign or borderline epithelium in the majority of mucinous ovarian cancer), have led to hypothesize a model of mucinous tumor progression. A mutation screening of RAS/RAF pathway in mucinous ovarian neoplasia showed that p16 loss and RAS/RAF pathway alterations are highly recurrent events, occurring early during mucinous tumor development: the frequency of concurrence of these events was observed in 40% of benign cystadenomas and 6% of borderline tumors [112]. The ensemble of these observations has given support to the hypothesis that benign cystadenomas and borderline tumors are precursors of mucinous ovarian cancer [112]. The key role of *KRAS* mutations in the development of mucinous ovarian tumors is supported also by recent studies. Particularly, Lee and coworkers have analyzed a set of mucinous ovarian tumors and have shown a prevalence of *KRAS* mutations of 0% in normal epithelial ovarian tissue, 57% in mucinous benign tumors, 90% in borderline mucinous neoplasms and 76% in malignant mucinous carcinomas [113]. Interestingly, in 15% of mucinous carcinomas, multiple *KRAS* mutations were detected, further supporting the key role of *KRAS* mutations in the pathway of neoplastic transformation involving mucinous adenoma-borderline tumor-carcinoma [113]. *RNF43* is a tumor suppressor gene frequently mutated in mucinous tumors of the ovary: 9% in borderline tumors and 21% in carcinomas [114]. A recent study explored the mutational landscape of mucinous ovarian carcinoma and its precursor lesion [109]. Benign and borderline mucinous tumors displayed and average of 25 (0.8 mutations/Mb) and 33 (0.9 mutations/Mb) mutations per tumor, compared to 67 mutations/tumor (1.5 mutations/Mb) observed in mucinous carcinomas [109]. The mutation spectrum was characterized by C > T transitions (64% of somatic substitutions). The genes more frequently mutated in these tumors included *KRAS, TP53*, *CDKN2A*, *BRAF*, *RNF43* (Figure 2). A minority of mucinous tumors does not display *KRAS* or *BRAF* oncogenic mutations; in these tumors, alternative mechanisms drive constitutive RAS signaling, such as *RRAS2* mutation, *HER2* amplification, *ARID1A* truncating mutation or *ELF3* mutation plus homozygous *CDKN2A* loss [115]. Another study performed a deep sequencing analysis of mucinous ovarian tumors, showing a comparable pattern of recurrently mutated driver genes, including *KRAS*, *CDKN2A*, *PIK3CA* and *PTEN*, except for *TP53* much more frequently mutated in carcinoma (57%) than in borderline (11.5%) tumors ([116], Figure 2).

Brenner tumors are rare ovarian tumors and account for approximately 1–2% of all ovarian tumors. The large majority of these tumors are benign, but 5% are borderline or malignant variants. Historically, Brenner tumors are commonly believed to derive from ovarian surface epithelia and associated with stroma; however, recently it was proposed a cellular origin for these tumors from Fallopian tubes and, particularly from benign clusters of epithelial cells that resemble transitional epithelium present in the connective tissue of fallopian tubes and commonly known as Wathard cell nests [117]. At histological level, benign Brenner tumors resemble nests or cords of transitional cells embedded in a stromal tissue; borderline cases contain some areas displaying cellular atypias; malignant cases show tissutal invasiveness and resemble transitional carcinomas of the urinary tract. Frequent is the finding of an association of a Brenner tumor with a mucinous tumor and evidence of mucinous metaplasia and cystic change is not rare. Several studies have evaluated the genomic alterations of Brenner tumors. Basically, these studies have shown that absence of recurrent mutations in benign Brenner tumors, while recurrent *KRAS*, *HRAS*, *PIK3CA* mutations and CDKN2A deletion are recurrent events in atypical proliferative Brenner tumors [118,119,120]; in benign Brenner tumors *MYC* amplification was observed in about 35% of cases [120]. Recently, Pfarr and coworkers have performed a next generation sequencing analysis of benign, borderline and malignant Brenner tumors and of ovarian carcinomas with transitional cell morphology. The analysis of Brenner tumors displayed the presence of sporadic (none recurrent) gene mutations, mostly occurring in genes involved in cell cycle control, DNA repair and epigenetic control [121]. All ovarian cancer with transitional cell morphology displayed *TP53* mutations, while all Brenner tumors were negative for these mutations [121]. No *TERT* promoter mutations were detected in Brenner tumors and in transitional ovarian cancers [121]. Interestingly, the analysis of copy number alterations showed that 75% (3/4) of the malignant Brenner tumors displayed *MDM2* modifications, in one case associated with *CCND*1 amplification [121]. These observations clearly support the conclusion that malignant Brenner tumors are genetically different from ovarian cancers with transitional-like cell morphology [121]. Interestingly, genetic studies on patients displaying combined mucinous and Brenner tumors showed that there is a shared clonal relationship of the two components, supporting the view that, in some cases, Brenner tumors may represent precursor lesions of mucinous tumors [122].

Clear cell carcinoma is a rare form of ovarian cancer, accounting for less than 10% of all ovarian cancers. This cancer subtype is associated with a reduced response rate following platinum-based chemotherapy and by a poor prognosis, particularly in advanced stage, in comparison with other ovarian cancer cell types. Ovarian clear cell carcinoma is characterized by a high degree of intratumoral cellular heterogeneity as shown by the presence in the tumors of both cells with differentiated properties and cells with consistent tumorigenic properties. Gene expression studies have provided clear evidence that clear cell ovarian cancer has a characteristic molecular signature that allows to distinguish these tumors from all other ovarian cancer types [116]. This analysis showed that there is a set of genes overexpressed only in clear cell carcinomas and not in other ovarian cancers; among these genes it is important to underline the strong overexpression of genes involved in oxidative stress response, such as Glutathione Peroxidase 3, Superoxide Dismutase 2 and Glutaredoxin, a finding that could help to explain the relative resistance of these tumors to chemotherapy [123].

Genomic analyses have provided evidence that clear cell ovarian cancers represent a heterogeneous group of tumors in terms of copy number aberrations. These studies have provided evidence about some abnormalities observed in these tumors: *PPM1D* was identified as likely amplicon driver and potential therapeutic target in clear cell carcinomas harboring 17q23.2 amplification [124]; recurrent amplification of the putative oncogene *ZNF217* [125]; frequent upregulation of ABCF2, associated with poor prognosis [126]. More recently, a high-resolution array comparative genomic hybridization analysis of ovarian cancer was performed. This analysis allowed to identify two clusters of patients: cluster 1 with a shorter median progression-free survival than cluster 2. 26 genomic regions with higher prevalence of high-level gain/amplification in cluster 1 compared with cluster 2 have been identified [127]. Several studies have identified mutations occurring in clear cell ovarian cancers. Some of these mutations were common to other ovarian cancer subtypes, while few of them are specific for these tumors. Thus, Kuo and coworkers have described some frequent gene mutations occurring in clear cell carcinoma at the level of *PIK3CA* (33%), *TP53* (15%), *KRAS* (7%), *PTEN* (5%), *CTNNB1* (3%) and *BRAF* (1%) (Figure 2) [128]. These observations have therefore indicated a high frequency of activating *PIK3CA* mutations in clear cell carcinoma [128]. In addition to the frequent *PIK3CA* mutations, deletions of *PTEN* have been observed in about 20% of ovarian clear cell carcinomas, thus supporting a role for aberrant PI3K/PTEN pathway in the development of this tumor.

More recent studies of exome sequencing performed on clear-cell ovarian cancer samples have provided definitive evidence that chromatin remodeling and PI3K are major drivers in clear cell carcinomas, with frequencies of *ARID1A* gene mutations of 60–65% and *PIK3CA* of 50–52% [129,130]. Copy number variation analysis identified frequent amplification in chr8q (64%), chr20q (54%) and chr17q (46%) loci, and deletion in chr19p (41%), chr13q (21%) and chr18q (21%) loci (Figure 2). Integration of the analyses showed that frequently mutated or amplified/deleted genes were involved in the KRAS/PI3K (82%) and MYC/Retinoblastoma (75%) pathways, as well as the critical chromatin remodeling complex switch/sucrose non-fermentable (85%) [130]. A screening of *TERT* promoter mutations in gynecological cancers provided evidence that these mutations are selectively observed in ovarian clear cell carcinomas, where they are observed with a 16% frequency [131]. Importantly, *TERT* promoter mutations were not detected in the contiguous endometriosis associated with ovarian clear cell carcinoma, and therefore do not appear to be an early event during oncogenesis of these tumors [131]. *TERT* promoter mutations tended to be mutually exclusive with loss of ARID1A protein expression and *PIK3CA* mutation [125]. *TERT* promoter mutations do not have a prognostic impact [131].

*Cyclin E1* overexpression and *CCNE1* copy-number gain occurred in about 23% and 15% of ovarian clear cell carcinomas, respectively; however, these abnormalities were not observed in other endometriosis-related tumors [132]. Cyclin E1 overexpression is positively correlated with hTERT promoter mutations, but not with the loss of *ARID1A* expression [132]. *CCNE1* overexpression is positively correlated with poor overall survival, even after adjustment for tumor stage [132].

More recent studies have led to the identification of some recurrent gene mutations that are frequently and specifically observed in clear cell carcinoma and are associated with endometrioid ovarian carcinomas, but not in other types of ovarian cancers. The presence of these mutations in both clear cell carcinomas and endometrioid carcinomas is not surprising since both these cancers are associated with endometriosis. Precancerous lesions, known as atypical endometriosis, have been identified in ovarian cancers as atypical epithelium in continuous with the malignant tumor. The genetic events associated with the transformation of endometriosis in ovarian clear cell carcinoma and endometrioid ovarian cancer are largely unknown. The gene *ARID1A*, encoding BAF250a, a component of the SWI-SNF chromatin remodeling complex, was found to be mutated in 46% of clear cell carcinomas, 30% of endometrioid ovarian cancer and never mutated in serous ovarian cancer [133]. Importantly, in some patients ARID1A mutations and loss of BAF250a protein have been observed in the tumor and the contiguous atypical endometriosis, but not in distant endometriotic lesions [69]. These findings were confirmed by a parallel study by Jones and coworkers showing ARID1A gene mutations in 57% of clear cell carcinomas [134]. These authors observed also mutations of serine/threonine phosphatase 2 gene *PPP2R1A*, occurring in 7% of patients with clear cell carcinomas [134]. The nature and pattern of the mutations suggest that *PPP2R1A* functions as an oncogene and ARID1A as a tumor suppressor gene [134]. The frequent mutations of *ARID1A* offered the opportunity to investigate the relationship existing between ovarian endometriosis and clear cell carcinoma. The relationship between these two diseases is underlined by various observations: (a) patients with ovarian endometriosis have an increased risk of clear cell carcinoma; (b) a high proportion of clear cell carcinoma patients undergoing surgery have also a simultaneous endometriosis. Importantly, loss of BAF250a protein expression was found in about 20% of benign ovarian endometriosis, in 24% atypical endometriosis and in 43% of clear cell carcinoma [135]. These observations suggest that a part of benign endometriosis have already undergone genetic alterations responsible for loss of BAF250a expression possibly giving an increased risk for malignant transformation [134].

Endometriosis, defined as the presence of endometrial tissue outside the uterine cavity, is a condition affecting up to 10% of the female condition. Endometriosis is commonly observed at the level of ovaries and of other pelvic structures. It is commonly believed that endometriosis develops either through metaplasia by retrograde or vascular dissemination during menstruation or, alternatively, from Mullerian developmental remnants. Endometriosis predisposes to the development of either clear cell carcinoma or endometrioid carcinoma. As mentioned above, mutational studies support a direct link between endometriosis and these ovarian cancers in a sequence of events involving first endometriosis, atypical endometriosis and then ovarian carcinoma. Epidemiological studies have shown a strong protective effect of bilateral tubal ligation against endometriosis-associated cancers, thus supporting retrograde menstruation as the main mechanism responsible for the development of cancer-associated endometriosis.

In direct support of these observations, a recent study reported the results of a study of exome sequencing of cancer-associated deeply infiltrating endometrioid lesions observed in 27 patients [135]. In this context, it is important to recall that endometriosis can be defined as the presence of ectopic endometrial stroma and epithelium; this condition affects about 10% of reproductive-age women and can cause pelvic pain and infertility. Three anatomical subtypes of endometriosis have been described: superficial peritoneal endometriosis, ovarian endometriosis and deep infiltrating endometriosis. The analysis of deep-infiltrating endometriotic lesions associated with cancer showed the frequent occurrence of somatic mutations (observed in 79% of cases); 26% of cases displayed driver mutations at the level of *ARID1A, PIK3CA, KRAS* or *PPP2R1A* genes (Figure 2); importantly, all the tested somatic mutations appeared to be confined at the level of the epithelial compartment of endometriotic lesions [136].

In line with these findings, other recent studies have confirmed that ARID1A mutations occur as an early event during clear cell carcinoma development and in the majority of cases coexist with *PI3KCA* mutations [137]. These observations indicate that both *ARID1A* and *PIK3CA* mutations occur early during clear cell carcinoma development and frequently coexist [131]. Tumors with loss of ARID1A expression were more likely to be chemoresistant than tumors with positive ARID1A expression and were associated with shorter progression-free survival [138]. Recent functional experiments have provided evidence that the *ARID1A* gene acts as a tumor suppressor. This conclusion is supported by the observation that restoring WT *ARID1A* expression in ovarian cancer cells harboring *ARID1A* mutations is sufficient to inhibit cell growth and tumor growth in xenotransplantation assays, while silencing of *ARID1A* expression in non-transformed ovarian epithelial cells is sufficient to stimulate cell proliferation and to induce tumorigenicity [139]. The tumor suppressor activity of *ARID1A* is mediated through activation of p21 expression, mediated via p53 [139]. These observations support the hypothesis that *ARID1A* may act as a tumor suppressor that cooperates with p53 to regulate p21 and tumor growth [139]. *ARID1A* somatic inactivating mutations are frequently observed in ovarian and uterine endometrioid and ovarian cell carcinomas. These tumors frequently display these mutations in association with *PI3KCA* or *PTEN* mutations, thus suggesting that these mutations could cooperate in tumorigenesis. This hypothesis was directly supported by the analysis of a conditional knockout mouse model in which *ARID1A* and *PTEN* were deleted individually or in combination: only double knockout mice developed ovarian endometrioid carcinoma [140]. The analysis of these mice provided evidence that coexistent *ARID1A-PIK3CA* mutations promote ovarian clear-cell tumorigenesis through induction of the production of pro-inflammatory cytokines, such as interleukein-6, whose signaling is essential in this process [141]. Other studies have shown the essential role for histone deacetylase 8 (HDAC6) in *ARID1A*-mutated ovarian cancers: inhibition of HDAC6 activity in these tumors inhibited cell growth and promoted apoptosis [142].

Another frequent abnormality observed in clear cell carcinoma is represented by overexpression of the membrane receptor C-MET, observed in 24% of these tumors [137]. The analysis of the tumors and of the associated endometriotic lesions showed that both lesions displayed *C-MET* overexpression [143]. C-MET overexpression was associated with higher tumor grade and poorly differentiated tumors [143]. Gene expression studies have shown that clear cell carcinomas have a peculiar gene expression signature allowing to distinguish this neoplasia from all ovarian cancer types [138]. The clear cell carcinoma signature contains genes typically expressed in this tumor type, such as Hepatocyte Nuclear Factor 1β (*HNF-1β*), Versican and other genes involved in oxidative stress response [144]. The clear cell carcinoma signature is largely dependent on the tumor microenvironment; furthermore, the expression of *HNF-1β* in these tumor cells was related to an epigenetic regulation of this gene through hypomethylation [143]. *HNF-1beta* overexpression plays a key role in the biology of clear cell carcinoma cells because promotes glucose uptake and aerobic glycolytic activity, known as the “Warburg effect”. The Warburg effect is important in the biology of cancer cells allowing these cells to survive under hypoxic conditions [145]. It is important to note that *ARID1A* mutations and *HNF-1beta* overexpression act at different stages of tumor development, genetic inactivation of *ARID1A* activating the carcinogenetic process, while epigenetic activation of HNF-1beta being involved in tumor phenotype and cancer progression [146].

Recent studies have in part clarified the problem of the molecular and cellular genesis of endometriosis-associated ovarian cancers. As above discussed, both clear cell ovarian cancer and endometrioid ovarian cancer harbor *ARID1A* loss-of-function mutations (occurring in 50% and 30% of cases, respectively), accompanied by *PTEN* loss and mutations in *PI3KCA*, *KRAS*, *CTNNB1*, *PPP2R1A* and *TERT* promoter, and by additional molecular defects, such as mismatch-repair deficiency, hypermutation and microsatellite instability. A recent study by Wang and coworkers provided fundamental evidences supporting a molecular diversity in the mutational processes and pathways generating these two types of ovarian carcinomas. In fact, using an integrate genomic approach, involving the analysis of whole-genome point mutation and of structural analysis (copy number alterations), these authors reached the important conclusion that clear cell ovarian carcinoma and endometrioid ovarian cancer can be stratified according to single nucleotide variation of mutational signatures. Two major subgroups of clear cell ovarian cancer were identified: C-PAOBEC and C-AGE. This stratification was based on the identification of regions of localized hypermutation at the level of genes which are members of C-APOBEC [27]. APOBEC signature association was independent of *ARID1A* and *PIK3CA* mutation status, since no differences were observed in the frequency of these mutations between the two C-APOBEC and C-AGE subgroups [27]. Clear cell ovarian cancers with a C-APOBEC mutational signature could be candidates for APOBEC targeting as a strategy to prevent ongoing clonal evolution in disease progression [27]. Endometriosis ovarian cancers can be subdivided into two subgroups (microsatellite stable (MSS) and microsatellite instable (MSI)) according to microsatellite instability: about one third of these tumors displayed microsatellite instability, with a mutational signature reflective of mismatch repair deficiency, a high proportion of frameshift indels, a high number of coding mutations and generation of neoantigens at a higher rate than other endometrioid ovarian cancers [27]. In endometrioid ovarian cancers, homozygous deletion in *PTEN* was mutually exclusive with *KRAS* mutation; furthermore, *TP53* was frequently mutated in MSS cases (40% of cases), while *RPL22* was frequently mutated in MSI [27]. The analysis of CNAs in these two subgroups provided evidence that MSS subgroup displayed recurrent focal somatic CNAs at the level of 3q26.1 encoding *MECOM* (amplification), of 8q24.21 encoding MYC (amplification) and of 10q23.31 encoding *PTEN* (deletion); CNAs were not observed in the MSI subgroup [27]. In conclusion, these observations indicate that different mutational processes are involved in the genesis of clear cell ovarian cancer and endometrioid ovarian cancer [27].

The pattern of gene expression of mucinous ovarian cancers resembles that observed in normal colon mucosa, while endometrioid and clear cell ovarian carcinomas correlated with normal endometrium [146]. These observations strongly suggest that mucinous ovarian cancer is a tumor very different from serous ovarian cancer [146]. Subsequent studies have analyzed in more detail the expression profile of mucinous ovarian adenocarcinomas and have shown a pronounced expression in these tumors of genes involved in tumor resistance and of genes involved in cytoskeletal regulation [147].

Other studies have explored gene expression profile in clear cell ovarian carcinoma. Histologically these tumors are “clear” due to their high cytoplasmic glycogen content and to the incapacity of hematoxilin and eosin staining to color this molecule. Initial gene expression studies have shown that clear cell ovarian tumors have gene expression profiles different from those observed in the other tumor histotypes. A recent study provided a detailed analysis of the whole genome expression profiling of clear cell ovarian cancer showing that major activated pathways involved in hypoxic cell growth, angiogenesis and glucose metabolism [148]. In line with this observation, clear cancer cell lines survive better than serous cell lines under hypoxia and glucose-deprived conditions and this is in part due to the activation of these pathways [148].

## 6. Molecular Abnormalities of Seromucinous Carcinomas of the Ovary

The 2014 World Health Organization Classification of Tumors of Female Reproductive Organs classified seromucinous carcinoma, a tumor exhibiting a great overlapping with other histiotypes of ovarian cancer, as a new ovarian cancer category. A recent study evaluated the genotyping and the immunohistochemical properties of these tumors. The immunophenotypic analysis showed that these tumors overlapped predominantly with endometrioid, and to a lesser extent with mucinous and low-grade serous carcinomas. Genomic and immunohistochemical alterations were detected in a number of genes, including *KRAS* (70%), *PIK3CA* (37%), *PTEN* (19%) and *ARID1A* (16%); no *CTNNB1* mutations were detected [149]. According to these observations, an endometrioid genotype was assigned to 60% of cases, a low-grade serous genotype to 30%, a mucinous genotype to 3% and 7% unclassified [150]. These observations support the conclusion that the category seromucinous carcinoma cannot be considered as a separate tumor type and that the large majority of tumors classified as seromucinous can be assigned to one of the major histotypes [150].

## 7. Molecular Abnormalities of Ovarian Granulosa Cell Tumors

Ovarian granulosa cell tumors are rare (2–5% of all ovarian cancers) tumors and represent the most frequent sex cord-stromal tumors. Two different types of these tumors have been described, juvenile and adult, based on the age of disease onset. Granulosa cells represent the cells of origin of these tumors, as supported by the observation that these tumors show histological, immunophenotypic and endocrinological similarities to normal granulosa cells. These tumors are classified as low-grade malignancies, but in some cases, may recur even many years after primary tumor resection. The molecular pathogenesis of granulosa-cell tumors is mainly related to the occurrence of the missense point mutation, 402C→G (C134 W) of the forkhead transcription factor *FOXL2*, essential granulosa-cell development, observed in 97% of cases of adult type tumors [151]. However, in juvenile-type granulosa-cell tumors *FOX2L* mutation was observed in only 10% of cases [151]. *FOX2L* mutations were observed in 21% of cases of thecomas, another type of sex cord-stromal tumors [151]. Thecomas are benign ovarian tumors composed only of theca cells. Theca cells are a group of endocrine cells in the ovary made up of connective tissue surrounding the follicule that has many diverse functions in folliculogenesis. Pure thecoma tumors are very rare, while mixed thecoma-fibromas are much more frequent; thecoma-fibromas, frequently display a variable mixed component of granulosa cells (10–50%): these tumors diplay FOXL2 mutations in about 50% of cases [152]. FOXL2 is one of the earliest markers expressed during normal ovarian development. FOXL2 promotes and maintains granulosa cell differentiation and survival, required for correct ovarian function.

Recently, Wang and coworkers have reported the wide genome sequencing of 10 cases of granulosa cell tumors and observed in 100% of these patients the pathognomic FOXL2 mutation; only few additional mutations were observed in these tumors: *TP53* mutation in 20% of cases and *PIK3CA*, *CTNNB1* and *PIK3R1* in 10% of cases [27]. In addition to these mutational events, highly recurrent chromosomal imbalances (the most frequent were trisomy 14 and monosomy 22), determining CNAs at the level of genes, such as *AKT1*, *RUNX1* and *LIMA1* [153]. The *FOXL2* mutation is maintained in recurrent adult-type granulosa cell tumors of the ovary and represents a very useful marker for the diagnosis of recurrence [154].

Although the presence of *FOXL2* mutations is almost constantly observed in granulosa cell tumors, some very are granulosa tumors are *FOXL2* WT. The clinical course of both *FOXL2* mutant and WT is similar [149]. Interestingly, some ovarian tumors are misdiagnosed, but are in fact other tumor types; the misdiagnosis of these tumors may have negative consequences for these patients because granulosa cell-type tumors are treated by surgical resection alone [149].

As mentioned above, *FOXL2* mutations are observed in only 10% of juvenile patients with granulosa ovarian cancers. Few studies have characterized the mutational spectrum of these juvenile tumors. A recent study reported in-frame duplications within the oncogene AKT1 in >60% of granulosa cell tumors [155,156]. No additional recurrent mutations were observed in these tumors [155,157]. Gene expression studies indicated a possible dedifferentiation process and the majority of the deregulated pattern of gene expression observed in these tumors could be related to a set of transcription factors perturbed by AKT1 activation [155,156].

In addition to *AKT1* mutations, an activating mutation R201C or R201H of the stimulatory g protein (GαS) was observed in about one third of juvenile granulosa cell tumors [158].

The key pathogenic role of AKT pathway in granulosa tumor development is supported by a recent experimental study showing that constitutive activation of PI3K in oocytes induces ovarian granulosa cell tumors [157].

## 8. Molecular Abnormalities in Ovarian Sertoli-Leydig Tumors

Ovarian Sertoli-Leydig tumors (OSLCTs) are a rare type of sex-cord stromal tumors, causing <0.5% of all ovarian tumors; these tumors usually occur in young women and are associated with an androgenic phenotype. The rarity of this tumor seemingly reflects its molecular pathway of tumor development, involving mutations promoting Sertoli cell generation from granulosa cells. The prognosis of these tumors is related to their differentiation, in that intermediate and poorly differentiated OSLCTs can recur after surgery debulking and require a medical postoperative treatment. These tumors have peculiar immunohistochemical and genomic properties. At histological level, these tumors contain Sertoli cells and Leydig cells, which are present in the male gonads and this explains the presence of an androgenic phenotype at clinical level. Both Sertoli and Leydig cells in OSLCTs share common molecular alterations, thus suggesting that both are derived from common progenitors during neoplastic transformation [159].

Fundamental in the study of these tumors was the discovery of recurrent (>50% of cases) mutations of the RNase IIIb with two endoribonuclease II-like domains DICER1: these somatic heterozygous mutations occur at the level of five different hotspot sites (E1705, D1709, E1788, D1810, E1813) [160,161]. The RNase III b domain of DICER1 cuts the miRNA strand, whereas the RNase IIIa domain of the enzyme cleaves the miRNA* strand. In the subsequent miRNA biogenesis, the RNA duplex is loaded into the RNA-induced silencing complex, containing the Argonaute protein. The miRNA* strand is then removed, leaving the miRNA strand, which interacts with its RNA targets, regulating their degradation or translation.

*DICER1* mutations are observed in 60% of OSLCT patients, including some cases with additional *DICER1* germline mutations [160,161]. *DICER1* mutations were observed also in 7% of patients with juvenile granulosa-cell tumors, in 13% of germ-cell tumors and in 12% of mature teratomas [160]. OSLCTs with hotspot *DICER1* mutations often have loss-of-function defects in the other allele due to germline or somatic events, thus developing a condition of *DICER1* deficient function, causing defective miRNA processing due to loss of 5p strand cleavage [162]. The mechanism through which *DICER1* mutations induce neoplastic transformation is related to the induction of an altered generation of miRNA species. Thus, it was shown that *DICER1* hotspot mutations are associated with the global reduction of 5p-derived miRNAs and the consequent deregulation of genes involved in the regulation of cell proliferation and differentiation [163].

More recent studies have explored the occurrence of *DICER1* mutations in larger series of OSLCTs, classified into well-differentiated, moderately differentiated and poorly differentiated. *DICER1* mutations were detected in 88% of patients: those pertaining to the poorly and moderately differentiated groups displayed in 100% of cases *DICER1* mutations, while no *DICER1* mutations were observed in well-differentiated OSLCTs [164].

Some patients developing OSLCTs have an autosomal dominant hereditary tumor predisposition syndrome, called DICER1 syndrome, caused by pathogenic variants to the *DICER1* gene [165]. These patients have an increased tendency to develop various types of tumors, including OSLCTs [165]. The tumors developing in these patients harbor biallelic pathogenic variants in *DICER1*, usually a loss-of-function pathogenic variant in one allele and a tumor-specific pathogenic somatic variant with mutation usually localized at the level of exons encoding the RNase IIIb domain [165].

To explain the androgenic phenotype observed in these tumors it is reasonable to assume that *DICER1* mutations determine abnormalities of miRNA-processing, disrupting the balance between the contrasting sex-determining signals in favor of a male phenotypic switch.

Interestingly, no DICER1 mutations were observed in HGS-OvCa; however, 60% of these tumors display reduced DICER1 levels, seemingly related to an epigenetic mechanism [166]. Importantly, HGS-OvCa patients with low DICER1 levels have a reduced survival, compared to those with normal/high DICER1 levels [167]. Furthermore, HGS-OvCa patients with high expression of DICER1 and DROSHA (another component of the RNA-interference machinery) have a clearly increased median overall survival [167].

## 9. Molecular Abnormalities of the Small Cell Carcinoma of the Ovary of Hypercalcemic Type (SCCOHT)

SCHHOT is an extremely rare, aggressive ovarian cancer type affecting children and young women. SCCOHT is the most common undifferentiated ovarian malignancy diagnosed in women under age 40. This is an aggressive tumor associated with a poor prognosis and scarce responsiveness to chemotherapy. SCCOHT is an aggressive tumor with only 30% survival in early stage disease. There is no standard treatment for this rare tumor. The prognosis of SCCOHTs is mostly related to the stage of disease [168]. The treatment associated with the best survival is surgical debulking and high-dose chemotherapy with autologous stem cell rescue [168]. The cell of origin remains unknown because neither the tumor histology, nor the immunophenotype are similar to those observed for any normal cell type of the ovary.

This tumor is characterized by a peculiar histology, immunohistochemistry and by a normal cytogenetic profile. Histologically, SCCOHT is composed of sheets of small, tightly packed cells with scant cytoplasm and hyperchromatic nuclei, with small nucleoli. However, about 50% of the tumors also contain a variable number of large cells with abundant eosinophilic cytoplasm, frequent rhaboid inclusions, large nuclei and prominent nucleoli. About 40–50% of tumors also contain variable numbers of large cells with abundant eosinophilic cytoplasm, often rhabdoid inclusions, large nuclei, and small nucleoli. Three recent studies showed that this tumor is characterized by a very frequent germline and somatic inactivating mutation of the SWI/SNF chromatin-remodeling gene *SMARCA4*, present in 69–100% of cases with a protein loss occurring in 82–100% of cases [168,169,170]. This abnormality was not observed in other ovarian cancer types and was, therefore, hypothesized to play a key role in the pathogenesis of this rare tumor. *SMARCA4* mutations are located at the level of various exons and include nonsense, frameshift and splice-site mutations and intragenic deletions of two exons [166,169,170].

Recently, a comprehensive genomic profiling of SCCOHT tumors was performed, showing that all cases displaying a classic morphology have a low tumor mutation burden (<6 mutations/Mb) and in 94% of cases display SMARCA4 mutations; in contrast, rare cases associated with large cell morphology are hypermutated (360 mutations/Mb) and are wild-type for *SMARCA4* [171].

At the level of genetic and epigenetic alterations SCHHOT resemble more atypical teratoid/rhabdoid tumors (ATRTs, characterized by inactivating mutations in SMARCCB1) than HGS-OvCas [171]. *SMARCA4* is the only recurrently mutated gene in SCCOHTs and the only recurrent allelic imbalance is observed exclusively on chromosome 19 [172].

The SMARCA4 gene mutations observed in SCCOHT determine a consequent loss of the SMARCA4 protein (also known as BRG1). The detection of SMARCA4 protein at immunohistochemistry level can be used as a diagnostic criterion, since SMARCA4 protein loss was very rare in other gynecological tumors (absent in HGS-OvCas, present in 4% of clear cell carcinomas, absent in endometrioid cancers and present in 8% of endometrial stromal sarcomas) [173]. According to these findings, it was suggested that *SMARCA4* loss could represent a pathogenetic molecular feature of SCCOHT. This issue was directly explored by Conlan and coworkers who have screened a large set of ovarian cancers, showing that *SMARCA4* loss was observed in 94% of SCCOHT samples, with the exception of a rare positivity (3%) in clear cell carcinomas [174]. Interestingly, at the level of gynecologic tumors, *SMARCA4* loss was also observed in poorly differentiated endometrioid adenocarcinoma of the uterus, a rhabdoid variant of this tumor [175]. A more recent study, based on an extensive screening, reported *SMARCA4*, *SMARCA2* and *SMARC1B* losses in 32%, 27% and 4%, respectively of undifferentiated carcinomas of the endometrium [176]. In about 70% of *SMARCA4*-deficient cases, simultaneous *SMARCA2* loss was observed [176]. Rhabdoid morphology was present only in a part of *SMARCA4* or *SMARCA2*-deficient endometrial tumors [176].

In SCCOHT, in addition to the loss of SMARC4 protein, is observed also of SMARCA2 (due to epigenetic silencing), the other mutually exclusive ATPase of the SWI/SNF complex [173,177]. About 90% of SCCOHTs have undetectable *SMARCA2* expression [177]. Depp sequencing studies showed that this loss of *SMARCA2* expression is not dependent upon inactivating mutational events [177]. Importantly, re-expression of *SMARCA4* or *SMARCA2* into SCCOHT cells inhibits cell growth, thus underlying the importance of dual loss of *SMARCA2* and *SMARCA4* in these tumors [173,177]. Therefore, dual loss of *SMARCA2* and *SMARCA4* is a molecular signature and defining feature of SCCOHT.

It is important to point out that the absence or not of SMARCA4 protein loss observed in SCCOHT and HGS-OvCa, respectively, may be related to the different types of *SMARCA4* mutations observed in these two tumors: in fact, in HGS-OvCas, *SMARCA4* missense mutations are reported and do not involve inactivation of the second allele; in contrast, in SCCOHTs, *SMARCA4* mutations are destructive to the protein, and are associated with biallelic inactivation due to mutation or loss of heterozygosity of the second allele [173]. The peculiar and restricted spectrum of genetic alterations observed in SCCOHT has led to hypothesize that the function on the SWI/SNF complex in transcriptional regulation, the absence of other recurrent mutations and other types of genetic alterations, and the diploid cytogenetic profile suggests that the main mechanism involved in the pathogenesis of this tumor is epigenetic, through functional inactivation of genes promoting cell differentiation and activation of genes stimulating proliferation [173]. Finally, it was suggested that SCCOHT may derive from the malignant transformation of rare resident ovarian cells.

As mentioned above, the SMARCAB1 subunit of the SWI/SNF complex is inactivated via bialleleic mutations in virtually all malignant rhabdoid tumors and atypical teratoid rhabdoid tumors. Initial studies have shown an antagonistic relationship between EZH2 (an enzymatic subunit of the polycomb-repressive complex 2, catalyzing the methylation of H3K27 and thus inducing transcription repression) and SMARCB1, thus rendering SMARC1-deficient tumors sensitive to AZH2 inhibition [178]. Interestingly, SCCOHTs, as well as rhabdoid tumors, depend for their survival on the EZH2 mehtyltransferase and, thus, are killed by potent and specific EZH2 inhibitors, such as tazemetostat [179]. Importantly, these results were observed both in SCCOHT cell lines and in xenografts from primary tumors [179]. A second recent study confirmed and extended these findings [180], showing that about 80% of SCCOHT tumor samples exhibit strong *EZH2* expression by immunohistochemistry; re-expression of *SMARCA4* in these cells reduced *EZH2* expression [180]. Furthermore, compared to other types of ovarian cancer cell lines, SCCOHT cell lines displayed a high sensitivity to the EZH2 inhibitors GSK126 and EPZ-6438 z which induced apoptosis and cell differentiation in SCCOHT cells [180]. Therefore, these studies suggest that loss of SMARCA4 determines a dependency on the catalytic activity of EXH2 in SCCOHT cells and that pharmacologic inhibition of EZH2 may represent a promising therapeutic approach for treating these tumors.

Familial SCCOHT tumors are rare, but the incidence of germline mutations in these patients is high, up to 43% of SCCOHTs. In some familial cases, the affected members carry tumors with both germline and somatic mutations. In this context, it is important to underline 11% of patients with Coffin-Siris syndrome (a congenital disorder characterized by developmental delay and intellectual and physical disabilities, caused by germline mutations in different subunits of the ATP-dependent SWI/SNF (SWItch/Sucrose Non Fermentable) chromatin remodeling complex) have *SMARCA4* germline mutations. In these patients, at variance with SCCOHT patients, *SMARCA4* mutations are non-truncating (either missense or small in-frame deletions). It is still not entirely clear why some mutations in the SWI/SNF complex predispose to cancer, while others lead to intellectual disability. Interestingly, it was postulated that the mutations in any member of the SWI/SNF complex that determine developmental defects xert either dominant-negative or gain-of-function effects, while those determining SCCOHT development are loss-of-function mutations [181]. In some of these patients, *SMARCA4* inactivating mutations were observed, in association with the development of SCCOHT [182]. Recently, Witkowski et al. reported two new familial cases of SCCOHT: affected members in both families were found to carry *SMARCA4* germline and somatic mutations, respectively, determining the loss of SMARCA4 protein in the tumors [183].

## 10. Normal Ovarian Stem Cells

The ovarian biology is based on the theory that oocyte reserve in female mammals involves a finite number of these cells established before or at birth and is basically determined by the number and quality of primordial follicles generated during the neonatal period. According to this theory, there is no need for germline stem cells in adult ovarian tissue. This theory explains why women undergo the menopause and suffer from premature ovarian failure if the primordial follicle pool is depleted by anticancer chemotherapy. This basic dogma was challenged by Johnson and coworkers, who following genotoxic treatment (busulfan) observed a re-establishment of the primordial follicle pool in mice [184]. This observation, however, has generated a great controversy and many other investigators have been unable to demonstrate the re-establishment of the follicle pool by adult germ cells in physiological and pathological conditions (reviewed in [185]). Support for the existence of ovarian cells with germ stem cell properties, called oogonial stem cells, derived from studies based on their isolation from mouse ovary; however, recent studies have shown that FACS-sorted putative oogonial stem cells isolated from mouse ovary do not have properties of germ cell [186]. Therefore, the actual prevailing evidence remains that the adult ovary is populated by a fixed number of oocytes and adult de novo oocyte production is a rare and physiologically insignificant event.

However, the ovulation process induces cyclic ruptures and regenerative repair of the ovarian surface (coelomic epithelium): this regenerative process seemingly implies the need for stem/progenitor cells. Thus, the existence of stem cells in the ovary regenerating the ovarian epithelium was supposed from long time, but not directly supported by specific experimental evidences. In this context, several studies have suggested the existence of epithelial cells, juxtaposed at the edges of ovulatory follicles and fimbrial fringes of the fallopian tube, exhibiting some properties of stem cells: few ovarian surface epithelial cells are capable of label retention, are quiescent but can be induced to cycle by estrous stimulation and are capable of in vitro colony generation [187,188]; few fallopian epithelial cells were characterized for the absence of markers of ciliated or secretory differentiated cells and by positivity for CD44 and integrin-l apha6 and for their capacity of clonal growth and self-renewal: these cells are particularly concentrated at the end of distal fimbries [189].

A more recent study identified the putative stem cell niche of the ovarian epithelium, first ruptured and subsequently regenerated during ovulation, at the level of the hilum region, represented by the junction area between ovarian surface epithelium, mesothelium and tubal epithelium [190]. Cells present at the level of the hilum display stem cell features, such as expression of the stem/progenitor cell markers CD133, LGR5, ALDH1, LEF1, and CK6B [190]. These cells display both in vitro (sphere generation) and in vivo (long-term lineage-tracing assays) stem cell properties [190]. The oncogenic transformation of these cells may generate high-grade serous adenocarcinomas in mice, as it will be discussed later [190].

The tubal-peritoneal junction connects the columnar epithelium of the fimbriated end of the fallopian tube to the flat mesothelial layer of the peritoneum. Interestingly, most of STICs are located in proximity of the tubal-peritoneal junction [191]. At the level of the tubal-peritoneal junction putative stem cells were identified, characterized by the expression of LEF1 (Lymphoid Enhancer-Binding Factor 1, a transcription factor that interacts with β-catenin in the nucleus as a component of WNT signaling), thus further supporting the existence of a stem cell niche within the tubal-peritoneal junctions [192]. All early dysplastic lesions (p53 signatures, SCOUT (secretory cell outgrowths) and STICs) display discrete patterns of LEF1 and β-catenin expression: particularly, LEF1 expression with strong nuclear location of β-catenin is indicative of SCOUTS, while LEF1 expression with membranous and cytoplasmic β-catenin is indicative of TP53 signatures and STICs [192].

## 11. Ovarian Cancer Stem Cells

HGSOC is the leading cause of morbidity and mortality from gynecologic malignancy. Despite the prolonged survival of HGSOC patients following the introduction of platinum/taxane based therapy, only 10–15% of these patients achieve long-term remission, while 70–90% of them relapse and die of their disease, raising the possibility that intrinsically chemoresistant cancer stem cells can account for cancer initiation, treatment failure and relapse.

A direct finding in support of this view comes from a recent study of Steg et al. [193]. In fact, these authors have shown that primary ovarian cancer specimens were composed of low densities of cells displaying cancer stem cell markers such as ALDH1A1, CD44 and CD133; tumors collected immediately after primary therapy were more densely composed of each of these markers. Tumors collected at first recurrence, before initiating secondary therapy, were composed of similar percentages of each marker as the primary tumors; however, in tumors collected from platinum-resistant patients CD133 expression was increased [193]. These observations support the view that in ovarian cancer, stem cell subpopulations contribute to tumor chemoresistance and, ultimately, to recurrent disease. These considerations have triggered consistent efforts aiming to isolate and characterize cancer stem cells from ovarian cancer samples.

A series of studies have attempted to identify membrane markers that could enrich for serous ovarian cancer initiating cells. The various studies carried out on serous ovarian cancer cells with heterogeneous cell populations (tumor cell lines, primary tumors, tumor xenografts and in vitro tumor-spheres) and with various methodologies claimed that CD144 with or without CD117 [194,195], Hoechst-excluding cells (the “side population”) [196], CD24, Epithelial Cell Adhesion Molecule (EpCAM) [195], CD133 [197,198,199,200] and Aldheyde Dehydrogenase-1A1 (ALDH1A1) [201] (Table 4) expression enrich for serous ovarian cancer tumor-initiating cells. Most of these studies performed on the characterization of ovarian cancer tumor-initiating cells show major limitations caused by the use of selected cells, represented either by xenografts of varying generations or by cells grown in differentiation-inhibiting media (a cell culture condition that favors tumor spheres formation).

In spite these limitations, some studies carried out starting from primary cell populations have provided some important informations. Thus, it was shown that the CD44^+^/c-kit^+^ population had an approximately 5000-fold increase in tumorigenicity, with tumors forming after the inoculation of as few as 100 cells from primary tumor, xenograft or in vitro-grown spheroid cell population [194]. In another study, it was reported that the CD133^+^ cell population exhibited an approximately 20-fold increased tumorigenicity, with tumor formation starting from 100–500 cells from murine xenografts [198].

The tumor cell populations used in these studies were very heterogeneous: cultured primary tumor cells [194,195,202,203], multiply passaged primary xenografts [96] or immortalized cell lines [196,197,201].

Importantly, a recent study described the development of a reliable in vivo assay to quantify ovarian cancer initiating cells in primary tumor cells. This assay is based on the inoculation of primary ovarian cancer cells depleted of leukocytes (CD45^+^ cells) into the mammary fat pad of NOD/SCID/IL-2Rγ^−/−^ mice [204]. In ovarian masses or omental metastases in the various ovarian cancer samples the frequency of TICs (Tumor-initiating cells) varied from 1/1400 to 1/911,000; the TIC frequency was higher in malignant ascites, ranging from 1/700 to 1/118,000 [204]. Taking advantage on the development of this TIC assay these authors have explored the existence of some membrane markers enriching for TICs in ovarian cancer. Among the various markers tested CD133 was the only enriching for TICs; however, this was not true in all ovarian cancer samples. In fact, TICs from some tumors are marked by CD133 expression; in other cases, lack of CD133 expression marks TICs; finally, in others, CD133 has no evident relevance to the TIC phenotype.

Kryczek et al. have explored the expression of various putative stem cell markers, including CD133, CD117, CD44, ABCG2, ESA (Epithelial Specific Antigen) and ALDH, in primary ovarian cancer samples and observed that: (i) these markers exhibited a highly heterogeneous expression; (ii) only CD133 and ALDH enriched for cancer-initiating cells, as assayed both in vitro by tumor sphere assay and in vivo after inoculation in NOD/SCID mice [205]. Although CD133 and ALDH can be used to identify and to enrich for ovarian cancer stem cells in fresh ovarian tumors, the expression of both markers is gradually reduced following prolonged in vitro cell passages, thus indicating that the in vitro cell culture conditions are not optimal for supporting stem cell properties, that probably need the presence of the tumor microenvironment. In this context, a recent study showed that human ovarian carcinoma-associated mesenchymal stem cells are non-tumorigenic, but clearly promoted tumor sphere growth in vitro, associated with an increase in tumor “stemness” [206].

A recent study provided additional data on CD133^+^/ALDH1^+^ ovarian cancer cells. Thus, Silva et al. showed that ALDH activity is present in a small percentage of ovarian cancer cells; small number of ALDH^+^ cells or ALDH^+^/CD133^+^ cells are capable of initiating and propagating tumors in nude mice; CD133^+^ cells were detectable in only 50% ovarian cancer specimens; these cells are able to generate in immunocompromised mice xenograft tumors similar to the original tumors [207]. These cells are chemoresistant and display angiogenic activity [207]. ALDH1 expression in ovarian cancer spheroids is induced by beta-catenin through a transcriptional mechanism involving occupancy of ALDH1 promoter by beta-catenin. In line with this observation, beta-catenin knockdown decreased ALDH1 expression levels [208]. Choi and coworkers have shown the existence of a hierarchy at the level of the CD133^+^/ALDH^+^ ovarian cancer cells, where CD133^+^/ALDH^+^ ovarian cancer stem cells give rise to a population of cancer stem cells/progenitors CD133^−^/ALDH^+^ which in turn originates CD133^−^/ALDH^−^ cells composed by tumorigenic/nontumorigenic cells [208]. The Bone Morphogenetic Protein 2 (BPM2) is expressed in ovarian cancer cells with an increasing gradient from CD133^+^/ALDH^+^ cells to CD133^−^/ALDH^−^ cells [209]. Importantly, BPM2 promotes the proliferation of CD133^+^/ALDH^+^ cells, while inhibits the growth of more differentiated ovarian cancer cells [208]. A recent study evaluated the possible clinical impact of CD133^+^/ALDH^+^ cells, showing that the double positive cells represent an independent prognostic factor for progression free intervals and overall survival [210]. The proportion of CD133^+^/ALDH^+^ cells remained similar in recurrent tumors, compared to primary tumors [210]. However, another recent study showed that ALDH1A HGS-OvCa positivity does not affect PFS or OS, but it is associated only with an increased platinum resistance/refractoriness [211]. It is important to note that recent studies do not have generated univocal results about ALDH1A expression in HGS-OvCa in that some reports showed an increased ALDH1A expression in advanced HGS-OvCa, compared to stage I/II tumors [212]. However, in contrast to these findings, Chui reported that the expression of ALDH1A was detected in both secretory and ciliated tubal epithelial cells, tubal-mesothelial junctions and ovarian surface epithelium, but was absent in serous tubal epithelial carcinoma and p53 signatures [213]. Positive staining in HGS-OvCa, when present, was limited to rare tumor cells. Analysis of TCGA data failed to identify any relationship between ALDH1A expression levels and overall survival [213]. These observations questionate the role and the significance of ALDH1A as a stem cell marker in ovarian cancer.

Katz and coworkers have used a very peculiar approach to characterize tumorigenic cells of human ovarian cancers. To this end, they have tried to define a peculiar microenvironment that could act in vivo as a supportive niche for human ovarian cancer cells; particularly, they have shown that teratomas derived from the inoculation of murine embryonic stem cells into nude mice represent a suitable microenvironment to support the growth of tumorigenic human ovarian cancer cells [214]. It is important to note that the ESC-originated cellular microenvironment in immunocompromised mice showed its consistent superiority to the standard tumor xenograft platform [215]. Using this system, it was shown that human tumor cells growing in ESC-derived microenvironment are heterogeneous, with a CD44^+^ALDH1^+^ progeny capable of sustaining tumor growth in vivo: these cells generate a more differentiated cell progeny CD44^−^ that, in turn, is able again to de-differentiate originating a self-renewing CD44^+^ cell population [216]. These observations highlight the plasticity of ovarian cancer stem cells and support the complexity to define this cell population. It is important to note that More and Alvero reported a heterogeneity of ovarian cancer tissue for CD44 expression [217]. According to CD44 positivity these authors distinguished two types of tumor cells: type I ovarian cancer cells, CD44^+^ALDH1^+^, MyD88^+^, large and non-dividing; type II ovarian cancer cells, CD44^−^, ALDH1^−^, MyD88^−^, small and rapidly dividing [217]. Type I cells exhibit several properties of cancer stem cells, including the capacity to generate tumors into immunodeficient mice, the capacity of generating CD44^−^ and of functioning as tumor vascular progenitors; type I cells are also chemoresistant [217].

Among the various cancer stem cell markers studied, ALDH seems to be one of the most reliable markers. Since clinical applicable-inhibitors of ALDH activity have not been reported, several recent studies have explored the factors responsible for ALDH expression in ovarian cancer cells. Thus, various biochemical pathways have been identified, required for ALDH expression in ovarian cancer cells. Bromodomain 4 (BDM4) was shown to be required for ALD1A expression in ovarian cancer cells; in line with this finding, Bromodomain and Extraterminal (BET) inhibitors suppress ALDH activity by abrogating BRD4-mediated ALDH1A expression [218]. BET inhibitors synergized with cisplatin in inducing the killing of ovarian cancer cells [218]. The alternative, non-canonical pathway of NFkB activation, mediated by RelB, is involved in promoting the expression of ALDH in ovarian cancer cells [219]. Other factors, such as EGFL6, a secreted protein that regulates stem cell proliferation and differentiation in various biological systems, is required for maintaining the stem cell activity of ALDH^+^ ovarian cancer cells, by regulating the asymmetric division potential and the metastatic capacity of these cells [220].

Recently, compound 673A, an ALDH inhibitor, was preliminary tested on ovarian cancer cells, showing that it induces necroptosis of ovarian cancer stem-like cells [221]. This compound was tested also in combination with cisplatin on tumor spheroids isolated from primary tumors: spheroids resistant to these two drugs have the tendency to the loss of CD133 expression and a decrease of ALDH activity [222].

Ovarian cancer stem cells express the oncoembryonic surface antigen, receptor tyrosine kinase-like orphan receptor 1 (ROR1): this antigen seems to play a role in the migration and invasion and spheroid formation in vitro and tumor engraftment in immunodeficient mice in vivo [223]. Treatment with a neutralizing antibody anti-ROR1 inhibits the capacity of ovarian cancer cells to form spheroids and to engraft immunodeficient mice and reduced the growth of tumor xenografts [223]. According to these observations it was suggested that ROR1, not expressed on adult tissues, could represent a relevant therapeutic target for ovarian cancer treatment [223]. Although these results are interesting, however, do not provide evidence whether ROR1 could represent a CSC marker for the whole tumor-initiating cell population and for all the patients. Importantly, ROR1 expression correlated with poor clinical outcome in ovarian cancers [224]. The development of specific monoclonal antibodies anti-ROR1 allowed a careful analysis of ROR1 positivity in various ovarian epithelial cancers, showing that 90% of endometrioid adenocarcinomas are ROR1^+^, 77% of HGS-OvCa papillary carcinomas are ROR1^+^, 44% of mucinous adenocarcinomas are ROR1^+^, while clear cell carcinomas are ROR1^−^ [225]. Importantly, most of positive tumors display a homogeneous positivity for ROR1 and exhibit membrane reactivity, thus supporting the therapeutic targeting of ROR1 [225].

The stromal cell-derived fractor-1 (SDF-1) or CXCL12/CXCR4 axis, critical for trafficking/homing of hematopoietic stem cells, represents an important functional axis in many solid tumors involved in the mechanism of tumor metastasis. Recent studies showed that CXCR4^+^/CD133^+^ cells, isolated from ovarian cancer cell lines, display properties of tumor-initiating cells, including phenotypic and functional properties [226]. The study of CXCR4 at the level of primary HGS-OvCa samples showed a high variability of expression, ranging from 0 to 61.2% (median value 2.4%) and was in part co-expressed with CD133 (CXCR4^+^/CD133^+^ cells corresponding from 0% to 18% of total cells) [226]. Subsequent studies have provided evidence that CXCR4 expression on ovarian cancer cells is involved in the mechanism of peritoneal and hematogenous dissemination [227,228]. Interestingly, alterations of the CXC4/SDF-1 axis are observed also in ovarian cancer precursor lesions, such as serous tubal intraepithelial neoplasms, secretory cell outgrowths and p53 signatures [229].

A recent study provided some evidence that putative ovarian cancer stem cells express mesenchymal markers. Thus, Ricci and coworkers have isolated a subpopulation of tumor cells from primary ovarian cancer samples propagating as non-adherent spheres in cell culture medium suitable for tumor stem cells [220]. The cells express several genes involved in chemoresistance and several mesenchymal markers: the expression of these mesenchymal markers is lost when the cells are grown in serum-rich medium, differentiate into the epithelial lineage and lose their invasive properties [230].

Other studies support the capacity of ovarian cancer stem cells to acquire mesenchymal markers and functional properties of mesenchymal cells. One study was carried out using a cell population CD44^+^MyD88^+^ enriched in epithelial ovarian cancer stem cells: this cell population exhibits multiple properties of ovarian cancer stem cells, including chemoresistance and the property to differentiate into multiple cell types [231]. These cells are able to generate a differentiated cell progeny, CD44^−^MyD88^−^ which have lost the self-renewal capacity, are more sensitive to chemotherapy and are differentiated [221]. CD44^+^MyD88^+^ epithelial ovarian stem cells grow as adherent cells, but when grown at high cell density are able to generate some spheroids, containing epithelial ovarian cancer cells that have undergone an epithelial-mesenchymal transition (as evidenced by the acquisition of mesenchymal markers and of TWIST-1, SLUG and VIMENTIN, mesenchymal markers associated with the process of EMT); the spheroid mesenchymal cells are able to revert to an epithelial phenotype when switched into appropriate cell culture conditions (i.e., they undergo MET) [231]. Interestingly, these authors showed that the intraperitoneal injection of CD44^+^MyD88^+^ epithelial ovarian cancer cells generated ovarian tumors, followed by carcinomatosis; mesenchymal spheroids were equally able to generate these tumors when inoculated into the peritoneal cavity; the tumors generated by both types of ovarian cancer cells have the same histological features [231]. Finally, they showed that TWIST-1 is essential to induce the EMT of CD44^+^MyD88^+^ cells: this factor is degraded into these cells, but is stabilized by hypoxia [231]. Jiang and coworkers have studied the side population (SP) and the non-side population into several ovarian cancer cell lines and tumor ascites: the SP population mostly contained ovarian cancer stem cells, while mesenchymal markers were mostly expressed among non-SP cells [232]. Although the tumorigenic potential was mostly at the level of the SP, the non-SP is required for the generation of SP cells and possesses more invasive properties [232]. Importantly, inhibition of the EMT process in these cells by SNAIL1 silencing decreased the ovarian cancer SP cell frequency [214]. These observations suggest that the SNAIL-regulated EMT is essential to epithelial ovarian cancer stem cells maintenance [232].

The capacity of ovarian cancer stem cells to express mesenchymal markers confer to these cells the capacity to adhere and to spread on mesothelial layers and, therefore, it may play an important role in peritoneal spreading of ovarian cancer. In vitro experiments have shown that ovarian cancer spheroids are able to attach and to clear a hole in a mesothelial cell monolayer through an integrin alpha5-dependent mechanism: inhibition of this integrin decreases the mesothelial clearance ability of ovarian cancer cells [233]. The capacity of ovarian cancer spheroids of clearance through mesothelium is associated with the expression of mesenchymal genes by these cells [234].

Some recent studies have investigated the potential clinical impact of EMT at the level of response to chemotherapy, progression-free survival (PFS) and overall survival (OS). Thus, Takai and coworkers have provided evidence that EMT status, as defined according to reduced E-Cadherin expression and the presence of nuclear SNAIL expression, is an independent predictor in patients with ovarian cancer, in that EMT^+^ patients have a shorter PFS and OS than EMT^−^ patients [235]. Tam and coworkers have developed a method for universal EMT scoring from cancer-specific transcriptomic EMT signatures of ovarian cancer and using this methodology have shown that EMT^+^ tumors have a shorter PFS and OS compared to epithelial ovarian cancers [236]. In a previous study, Tan and coworkers have generated a classification system of ovarian cancer: according to this system these tumors have been classified in 5 subgroups, including Epi-A, Epi-B, Mes, Stem-A and Stem-B, of which Epi-B and Stem-A signatures were independent prognosticators of good and poor survival, respectively [39]. In a subsequent study the same investigators have evaluated the expression of 10 protein products of genes identified by microarrays as differentiators between the five subgroups, involved in either stemness or EMT, 4 down-regulated and 6 up-regulated in either Mes or Stem-A ovarian cancers [233]. Thus, E-Cadherin, P-Cadherin, Zeb1, HMGA2, Rab25, CD24, NCAM (CD56), Sox11 and Vimentin expression was assessed in 100 advanced-stage ovarian cancer patients undergoing platinum-based chemotherapy [237]. NCAM emerged as a prognostic marker, its expression being associated with a shorter survival, and Zeb1 as a marker of poor chemoresponse [237].

Some studies have tried to characterize cancer stem cells present in ascites. The presence of ascites in ovarian cancer was associated to a poor prognosis. Within the ascites are present both single cells and spheroids and are present normal inflammatory cells and tumor epithelial cells. Latifi and coworkers have recently reported a procedure to enrich for cancer stem cells present in ascites and to separate these cells from mesenchymal cells: ascitic cells, after RBCs lysis, are separated on low attachment plates into an adherent cell population enriched in mesenchymal cells and a non-adherent cell population, forming spheroids and enriched in epithelial cells [238]. Tumorigenic cells have been detected among the non-adherent cell population. Interestingly, ascitic cells isolated from relapsing patients are predominantly epithelial and show a pattern of gene expression associated with cancer stem cells, compared to cells of patients isolated from primary tumors [238]. As mentioned above the transcription factor FOXM1 is frequently overexpressed in ovarian cancer and recent data suggest a possible role for its deregulated expression at the level of the cancer stem cell population. A recent study based on the analysis of 158 ovarian cancer patients indicates that overexpression of FOXM1 predicts poor prognosis, promotes the expression of VEGF-A, stimulates cell proliferation, migration and tissue invasion [239]. The molecular mechanisms responsible for FOXM1 overexpression in ovarian cancer are largely unknown. However, a recent study suggested that a relevant role could be played by TP53 mutations: in fact, TP53 mutants are unable to exert the repressive effect displayed by WT TP53 on FOXM1 expression [240]. Another recent study provided direct evidence that the FOXM1 overexpression confers highly malignant properties to ovarian cancer cells, consisting in EMT, stemness and chemoresistance [241]. Particularly, FOXM1 expression enhanced tumorsphere formation and increased the expression of various stem cell markers [241].

As mentioned above, one of the putative membrane marker of ovarian cancer stem cells is CD117, the receptor for the cytokine Stem Cell Factor. C-kit activating mutations have not been found in ovarian cancer, but c-kit is overexpressed in some cases and its overexpression is correlated with poorer patient outcome. Chan and coworkers have isolated cancer stem cell lines from two ovarian cancer cell lines and have demonstrated that these cells express both c-kit and its ligand SCF; blockade of c-kit expression in these cells exerted an inhibitory effect on cell proliferation and on the expression of other stem cell-associated markers [242]. C-kit inhibitors cooperated with platinum compounds to inhibit these ovarian cancer cells [242]. The analysis of the mechanisms through which c-kit sustains the tumorigenic potential of ovarian cancer stem cells indicates a stimulatory effect of this receptor on the activation of Wnt/beta-catenin pathway [243]. CD117^+^/CD44^+^ cells were characterized for some biologic properties, possible suitable for therapeutic targeting. Thus, CD117^+^/CD44^+^ ovarian cancer cells were shown to have a higher basal autophagy than the CD117^−^ counterpart; inhibition of this pathway by cloroquine treatment inhibits the growth and the survival of ovarian cancer spheroids and their tumorigenic activity in vivo [244]. CD117^+^/CD44^+^ cells isolated from the ascitic fluid of ovarian cancer patients are very sensitive to the anti-cancer stem cell antibiotic salinomycin: this drug added alone induced an inhibition of stemness markers of CD117^+^/CD44^+^ cells, such as SOX2 and OCT 3-4 and added together with paclitaxel induced apoptosis of these cells [244]. About 25% of HGS-OvCa patients display overexpression of CD117; a meta-analysis on more than 1200 patients reported in literature clearly showed that CD117 overexpression was associated with reduced overall survival [245].

Zhang and coworkers have isolated a very peculiar subpopulation of ovarian cancer cells endowed with high tumorigenic potential. This cell population was selected according to its big size and DNA content (polyploidy giant cancer cells). These cells were isolated from primary tumor tissues and ovarian cancer cell lines, after treatment with cobalt chloride, an agent inducing hypoxia [2]. The isolated giant polyploidy cancer cells were shown to be highly tumorigenic, to express several cancer stem cell markers, to be resistant to both standard chemotherapeutic agents and hypoxia, and to be able to generate standard not giant tumor cells [246]. According to these findings, it was hypothesized that hypoxia stress may represent an important mechanism through which the tumor induces the formation of giant polyploidy cancer cells that in turn contribute to the generation of cancer stem-like cells [246].

Tumor microenvironment plays a major role in the control of tumor growth, including cancer stem cells. The tumor microenvironment in which ovarian cancer develops is characterized by the presence of a wide spectrum of inflammatory cytokines and chemokines, playing a key role in the control of tumor development. These inflammatory cytokines may affect also CSCs and the tissutal niches in which these cells grow. In this context, a recent study identified IL-17 as an inflammatory cytokine present at the level of tumor microenvironment and capable of affecting the self-renewal of ovarian cancer stem cells [247]. IL-17 is a pro-inflammatory cytokine that is produced by activated CD4^+^, CD8^+^ T cells and by macrophages; IL-17-positive cells are observed at the level of ovarian cancers [247]. IL-17 was shown to stimulate in vitro the growth of ovarian cancer tumorspheres initiated by CD133^+^ cells through stimulation of the IL-17 receptor present on the surface of these cells [247]. This stimulatory effect of IL-17 on self-renewal of ovarian cancer stem cells seems to involve NF-kB and p38 MAPK: in line with this observation, these effects were blocked by NF-kB and p38 MAPK inhibitors [247].

One of the most important causes of treatment failure in epithelial ovarian cancer treatment consists of the development of residual and recurrent tumor cells that are resistant to cisplatin and paclitaxel treatment. One of the main reasons responsible for recurrence is related to the incapacity of platinum-based chemotherapy regimens to remove cancer stem cells: in fact, these drugs, while they are extremely efficient in removing the bulk of ovarian cancer cells, are, however, unable to eliminate the population of cancer stem cells. Thus, Latifi and coworkers have shown that cisplatin induces an epithelial to mesenchymal transition in ovarian cancer cells derived from advanced stage patients: tumor cells surviving to cisplatin treatment in vitro changed their morphology from a typical epithelial to a typical mesenchymal pattern, reduced E-cadherin and increased N-cadherin and vimentin expression; in parallel, it was observed an increased expression of stem cell-associated membrane markers such as CD133, CD44, CD117, EpCAM and of the stem cell factors Nanog and Oct-4 [248]. Recurrent ovarian tumors are enriched with cancer stem cell-like cells and stem cell mediators, such as CD44, CD133, NOTCH, Wnt, TGFbeta, ALDH1, thus supporting the hypothesis that cancer stem cells contribute to the disease recurrence [192].

Few studies have explored the sensitivity to chemotherapic agents of enriched populations of ovarian cancer stem cells. In this context, a recent study showed a very intriguing finding. In fact, Meirelles and coworkers isolated from ovarian cancer cell lines a fraction of stem/progenitor cells (isolated according to CD44, CD24 and EpCAM positivity and E-cadherin negativity) and showed that, while unseparated original cell lines are inhibited by these drugs, these cells were stimulated by doxorubicin or cisplatin, [249]. In contrast, these immature cells were inhibited by Mullerian inhibiting substance, a member of the TGF superfamily [239]. Using a high throughput approach many chemical compounds have been screened for their property to inhibit ovarian cancer stem cells [250]. This approach allowed the identification of a new agent, niclosamide, commonly used against numerous parasites, exhibiting the capacity to inhibit the growth of ovarian tumor-initiating cells [240]. Niclosamide represses metabolic enzymes responsible for bioenergetics, biosynthesis and redox regulation, seemingly leading to mitochondrial “intrinsic” apoptosis pathway, loss of tumor stemness, and growth inhibition [250]. It is of interest to note that niclosamide was found to be active against leukemic stem cells, due to its inhibitory properties on NF-kB activation. The inhibitory effect of niclosamide on ovarian cancer stem cells could be also related to the capacity of this compound to inhibit Wnt signaling, a pathway essential for the survival of ovarian-initiating cells.

Other recent studies have explored the possible targeting of NOTCH as a therapeutic strategy to inhibit ovarian cancer stem cells. In ovarian cancer NOTCH3 is believed to play a key role: this receptor was found to be overexpressed in about 20% of serous ovarian cancers that are associated to a poor prognosis. It was shown that NOTCH3 plays a key role in the control of ovarian cancer stem cells: in fact, NOTCH overexpression in ovarian cancer cells determines an expansion of cancer stem cells, while NOTCH signaling inhibitors (gamma-secretase inhibitors) decrease the number of cancer stem cells; interestingly, the combination of gamma secretase inhibitors (NOTCH inhibitors) and platinum exerts a complete inhibitory effect on cancer stem cells [251]. This observation indicates that the combination of NOTCH inhibitors with platinum may represent a promising strategy for ovarian cancer treatment [251]. A recent study in part clarified the molecular mechanisms responsible for NOTCH3 overexpression in ovarian cancer. Thus, Jung and coworkers have observed the frequent homozygous and heterozygous deletions in the WWP2 locus, encoding the E3 ubiquitin-protein ligase WWP2: the monouquitination of NOTCH3 by WWP2 targets an endosomal/lysosomal degradation fate for NOTCH3 [252]. Interestingly, ectopic expression of WWP2 decreases tumor development in a mouse xenograft model and suppresses NOTCH3-mediated effects, including increase in cancer stem cell-like cell population and platinum-resistance [252].

One of the main features of ovarian cancer stem cells, like other cancer stem cell populations, consists of the increased expression of some transcription factors essential for induction of the stemness phenotype. Among these transcription factors a relevant role is played by NANOG. Recent studies indicate that this transcription factor plays a main role in the progression of ovarian cancers. NANOG expression in primary ovarian cancers was found to be correlated with disease grade, reduced chemosensitivity and poor survival [253]. Functional experiments showed that NANOG expression is required for ovarian cancer migration and invasion, through regulation of E-Cadherin and FOXJ1 [253]. Interestingly, a recent study showed that metformin inhibits the formation of tumor spheroids by ovarian cancer cells through a mechanism involving an increased FOXO3 nuclear localization and a consequent inhibition of the expression of several stemness markers, including Nanog, Oct-4 and c-MYC [254]. On the other hand, recent studies suggest an important role of the transcription factor SOX2 in maintaining the cancer stem cell state of ovarian cancer cells: in fact, in serous ovarian cancer cells SOX2 expression increases the expression of cancer stem cell markers, the potential to form tumor spheres in vitro and the tumorigenic potential in vivo; furthermore, SOX2-expressing ovarian cancer cells display enhanced resistance to apoptosis induced by chemotherapy or by cell death inducing agents, such as TRAIL [255]. SOX2 expression in primary ovarian cancer samples was heterogeneous and positive cases displayed not more than 10% positive cells. Thus, inhibition of SOX2 in ovarian tumor spheres not only reduced the expression of stemness markers, but decreased also the resistance to cisplatin treatment and the in vivo tumorigenicity [256]. Importantly, SOX2 expression was closely associated with chemo-resistance and poor prognosis [257]. SOX2 expression is increased in HGS-OvCa and, also, in precursor lesions of *BRCA1/BRCA2* carriers, a phenomenon seemingly related to frequent mutations occurring at the level of 40kb distal repressor region of *SOX2* gene [258]. SOX2 expression in ovarian cancer cells is induced by a regulatory pathway involving hypoxia-mediated NOTCH1 activation [259].

Recent studies have shown that RAD6, an ubiquitin-conjugating enzyme originally identified as a DNA repair protein, plays a key role in the induction of SOX2 expression in ovarian cancer cells and in the maintenance of a cancer stem cell phenotype [260,261]. RAD6A and RAD6B regulate mutagenic DNA damage tolerance and Fanconi anemia DNA repair pathways in response to various genomic insults (chemo and radiation therapy); furthermore, RAD6 regulates gene transcription in ubiquitination-mediated in association with RNF20/40 ubiquitin ligase by histone 2B ubiquitination-mediated chromatin modifications. RAD6 is overexpressed in HGS-OvCa and its levels strongly correlate with ovarian cancer progression; in ovarian cancer cells RAD6 stabilizes beta-catenin promoting stem cell-characteristics, including expression of stem cells markers such as SOX2 and ALDH1A and anchorage-independent growth [260,261]. Downregulation of RAD6 or its inhibition using a small molecule inhibitor attenuated DNA repair signaling, expression of cancer stem cell markers and increased the sensitivity of chemoresistant cells to carboplatin [261,262].

Hypoxia plays an important role in the modulation of the biological properties of ovarian cancer cells. Ovarian cancer cells are resistant to hypoxia which promotes in these cells angiogenetic mechanisms and tumor evasion. Thus, recent studies have shown that hypoxia induces in ovarian cancer cells the expression of chemotactic factors that promote immunological tolerance and angiogenesis: thus, tumor hypoxia promotes the recruitment of T regulatory cells through induction of expression of the chemokine ligand 28 (CCL28) which, in turn, promotes tumor tolerance and angiogenesis [263]. In addition to the important effects on the biology of ovarian cancer cells, the hypoxic microenvironment stimulates stem-like properties of these tumor cells. Thus, growth of ovarian cancer cell lines under hypoxic conditions (i.e., 1% O_2_) resulted in a clear stimulation of the expression of several ovarian cancer stem cell markers [264].

Other studies have evaluated the possible clinical implications at the level of prognostic criteria and response to therapy, related to the level of expression of stem cell markers in ovarian cancer specimens. The levels of CD133 expression in advanced serous ovarian cancers do not were associated with chemoresistance and shorter overall survival; in contrast, the expression of another stem cell-associated marker, nestin, correlates with cisplatin-resistance and poor survival [265]. In contrast to these findings, another study carried out in 400 patients provided different evidences. In fact, in this study CD133 expression was observed in 31% of ovarian cancer samples; CD133 expression was associated with high-grade serous carcinoma, late-stage disease, ascites level and absence of response to therapy [266] Furthermore, CD133 expression was associated with shorter overall survival time [266].

In contrast to CD133, the level of expression of CD44^+^/CD24^−^ cells in ovarian cancers seems to have a prognostic value. In fact, patients with >25% of CD44^+^/CD24^−^ cells have more tendency to recur and shorter median progression-free survival than those with <25% CD44^+^/CD24^−^ cells [267]. Other studies have explored the frequency of ovarian cancer stem cells, identified as CD44^+^ALDH1^+^, in a large number of primary tumors: according to the level of CD44 expression the patients were subdivided into two subgroups, high expression (>20% CD44^+^ cells) and low expression (<20% CD44^+^ cells). High CD44 expression was preferentially observed among patients with FIGO stage I disease, thus indicating that in patients with primary disease OCSCs are preferentially observed in early stage tumors and their number decreases as the disease progresses [267]. Among patients with stage I disease, those exhibiting low CD44 expression have a better response to chemotherapy compared to patients with high CD44 expression [268]. Finally, patients with high CD44 expression had significantly shorter progression-free survival than patients with low CD44 expression level [252]. In another study, it was analyzed the possible prognostic impact of the level of ALDH1 expression as assessed by immunohistochemical analysis. This analysis showed that patients with higher ALDH1 expression had poor overall survival, compared to those with lower ALDH1 levels [269]. These observations were confirmed by Liebscher and coworkers, showing that ALDH1^+^ high-grade serous ovarian cancers have a negative prognosis: particularly, the concomitant expression of ALDH1 and EGFR identified a subpopulation of ovarian cancer patients displaying a highly aggressive, poor prognosis disease [270].

As stated above, 20 years ago the standard first line treatment of ovarian cancer consisted of the combined administration of cisplatin and paclitaxel. These two drugs act through different mechanisms leading to apoptosis of ovarian cancer cells: cisplatin is a DNA strand cross-linking drug that generates DNA damage and consequent activation of cyclin inhibitors such as p21, with the cell cycle arrest in G1 or G2 phase; paclitaxel is a mitotic inhibitor, promoting the formation and stabilization of microtubules, with consequent cell cycle block at the level of metaphase or anaphase. Various mechanisms of resistance to cisplatin have been observed, including increased glutathione and metallothionein levels, increased drug efflux, decreased drug uptake, increased DNA repair and tolerance to the formation of platinum-induced DNA adducts; furthermore, there is a relationship between the p53 mutation status and cisplatin resistance. In contrast to cisplatin, the paclitaxel sensitivity is p53 status-independent. First, the existence of an association between chemoresistance and the occurrence of epithelial mesenchymal transition (EMT) and CSC-like phenotype in ovarian cancer was demonstrated [248,271]. In this context, particularly interesting was a recent study showing that paclitaxel treatment can induce at the level of epithelial ovarian cancer stem cells molecular changes favoring the acquisition of mesenchymal properties, while maintaining their stamina properties [272]. These changes induce the generation of a population of cancer stem cells that are more chemoresistant [272].

Second, ovarian cancer stem cells are in part resistant to chemotherapy agents. This conclusion derives from several recent observations. Thus, Rizzo and coworkers have found that ovarian cancer stem cells, identified on the basis of the side population assay, are increased in their number in ascites from patients who have relapsed following chemotherapy compared to chemonaive patients, consistent with the hypothesis that this cell population in part survive and is selected by platinum-based chemotherapy [273]. Side population cells highly express the drug efflux transporter ABCB1 and the histone methyltransferase EZH2, both contributing to drug-resistance of these cells [273]. These findings were corroborated by studies carried out on ovarian cancer cell lines and showing that cells surviving to platinum selection are enriched in cancer stem cell markers and endowed with a high tumorigenic potential [274]. Gene expression studies have shown that the stem cell population signature was enriched in patients with early recurrence (up to 1 year), compared with those with a later (1–2 years) recurrence [275]. On the other hand, experimental studies have shown that a short-term single exposure of standard chemotherapy treatment (cisplatin, paclitaxel or both agents) induces in surviving ovarian cancer cells a CSC-like phenotype, which is independent of the type of chemotherapy [276]. Importantly, chemotherapy-surviving cells were more capable than untreated cells to form tumors into immunodeficient mice and maintained this property over time [276].

Very few studies have explored tumor stem cell populations into clear cell ovarian cancers. In this context, Katz and coworkers have been able to grow primary clear cell ovarian cancer primary cells into immunodeficient mice using human embryonic stem cells to generate a suitable tissue microenvironment into these mice [210]. In a second study, the functional properties of six clonally expanded cell populations derived from a single ovarian clear cell carcinoma patient have been characterized. Interestingly, each of the six clonally expanded subpopulations display a different level of morphologic differentiation and tumorigenic potential; the growth of these cells into a human embryonic stem cell-derived microenvironment favors the growth of stem-like CD44^+^/ALDH^+^ cells [216]. These cells sustain tumor growth and exhibit the capacity of tumoral cell differentiation, generating CD44^−^ cells: in turn, these cells are able to restore cancer stem cell properties, reacquiring CD44 expression and self-renewing properties [216]. These observations suggest that ovarian clear cell carcinoma-derived stem cells possess a consistent degree of plasticity and this property may significantly contribute to both tumor heterogeneity and to chemoresistance, two typical features of this tumor. A recent study suggested that ALDH1^high^ cells may represent cancer stem cells in clear carcinomas. In fact, ALDH1^high^ cells isolated from primary cancer stem cells displayed a markedly higher tumorigenic potential than ALDH1^low^ cells [277]. About 15% of cancer stem cells were shown to be associated with a high expression of ALDH1^high^ cells and these tumors have a negative prognosis [277]. Other studies have shown that ALDH1 expression increases in the advanced stages of clear cell ovarian carcinoma [278]. Interestingly, ALDH1-high cells isolated from clear cell carcinoma cell lines displayed a high level of anti-oxidant enzymes, such as NRF2 and the elevated expression of ROS scavenger systems in these cells contribute to their chemoresistance [278]. The study of clear cell ovarian cancer spheroids allowed to show that these cells are sensitive to the additive effects of targeting of glutamine metabolism and of focal adhesion kinase (FAK) to inhibit mTOR pathway and the growth of these cells [279].

## 12. MiRNAs and Ovarian Cancer Stem Cells

Studies carried out during the last years have indicated an important role for microRNAs in ovarian cancer tumorigenesis. Several studies have reported that several miRNAs are aberrantly expressed in ovarian cancer through genomic and epigenetic mechanisms [280]. In spite of the heterogeneity observed at the level of the results due to differences in types of tumor specimens, heterogeneity of the tumors, RNA isolation protocols and detection platforms, 39 miRNAs consistently showed a differential expression in ovarian cancer compared to normal ovarian tissue [281]. Some of these miRs were found to be markedly altered in ovarian cancer and have been linked with disease progression/survival and with ovarian cancer stem cells.

In this context, particularly relevant seems to be the role of miR-200 and Let-7 families in ovarian cancer. The miR-200 family consists of five members (miR-200a, miR-200b, miR-200c, miR-141 and miR-429). These miRNAs form two clusters: cluster 1, composed by miR-200b, miR-200a and miR-429 maps to chromosome 1 (1p33.36), while the cluster 2, composed by miR-200c and miR-141, maps to chromosome 12 (12p33.31). The expression levels of the miR-200 family are deregulated in ovarian cancer patients. In ovarian cancer tissues, miR-200s are usually upregulated in tumor tissues compared with the normal ovarian tissue, with variable levels of expression in function of tumor stage and histology [282,283]. Given the main function of this miRNA family in suppressing epithelial-to-mesenchymal transition, determining overexpression of E-Cadherin, epithelial cell identity and cancer metastasis inhibition [284,285], it is not surprising that it was suggested that the main consequences of miR-200 family overexpression consist of enhancing the epithelium phenotype of these tumor cells [286]. Particularly, it was shown that miR-200a targets p38 and, through this mechanism, controls oxidative stress response: ovarian cancer patients with low miR-200a expression had poor prognosis compared with patients with high miR-200a expression [287]; miR-200c was found to be deregulated among stage I epithelial ovarian cancer patients who relapsed, compared with non-relapsers and was associated with patient survival [288].

The miR-200 family plays a critical role in the suppression of epithelial-to-mesenchymal transition (EMT) and tumor cell migration, invasion, and metastasis by directly targeting ZEB1 and ZEB2 transcription factors and the level of miR-200s are important regulators of EMT in ovarian cancer cells [284,285,286]. miR-200s inhibit angiogenesis through direct and indirect mechanisms by targeting interleukin-8 and CXCL1 secreted by the tumor endothelial and cancer cells; in experimental models, miR-200 delivery into ovarian cancer cells inhibit angiogenesis [289]. Previous studies have shown that a downregulation of miR-200 expression is required for maintenance of various types of cancer stem cells. In line with these findings miR-200a levels were found to be downregulated in CD133^+^ ovarian cancer cells compared to the levels observed in CD133^−^ cells: overexpression of this miR in CD133^+^ cells inhibited cell migration and invasiveness through a mechanism at least in part mediated through E-Cadherin repressor ZEB2 targeting with consequent increase in E-Cadherin expression level [290]. In conclusion, the studies of miR-200 family indicate a role for these miRNAs in EMT in ovarian cancer [284]. However, their exact role in tumor initiation, progression, metastasis and chemoresistance is unclear and, at the moment, it is difficult to predict whether a therapeutic increase of miR-200s levels or a decrease of their levels in ovarian cancer could could exert a beneficial or detrimental effect [284].

Another miR whose expression is frequently deregulated in ovarian cancer in ovarian cancer is the Let-7 family, which consists of 10 mature miRNAs (Let7a–g/I, miR-98 and miR-202) organized in several clusters. Four Let-7 family members are downregulated in ovarian cancer. Low Let-7b levels are associated with poor prognosis in serous ovarian cancer; similarly, expression levels of high LIN 28 and LIN 28 B, which act as inhibitors of Let-7 miRNA processing correlate with reduced survival in ovarian cancer patients. The mechanism through which Let-7 favors ovarian cancer tumor progression is related to the capacity of this miRNA to target various oncogenes such as HMGA-2, MYC and K-Ras. Various lines of evidence suggest that Let-7 deregulation play a role in ovarian cancer stem cell maintenance. In this context, initial studies have explored the expression of LIN 28 in ovarian cancer stem cells. This type of study was stimulated by the observation that some proteins, including LIN 28 and Oct4 were expressed in embryonic stem cells. The study of these two stem cell markers in ovarian cancers showed a predominant expression in advanced tumors and at the level of a sub-population of stem-like cells [291]. Additional studies confirmed the idea that Lin 28 may play a role in biology of ovarian cancer stem cells. Thus, Yang et al. provided evidence that: (i) Lin 28 expression in ovarian cancer specimens correlates with ALDH1 expression; (ii) knockdown experiments provided evidence that Lin 28 plays a functional role in the maintenance of ALDH1^+^ tumor cells; (iii) Lin 28 in ovarian cancer cells modulates the biogenesis of miRNA Let-7 in tumor cell; (iv) Lin 28 maintains the ALDH1^+^ cell population by modulating miRNA Let-7 maturation: patients with low Let-7 expression have high percentages of ALDH1 positive cells, while the opposite phenomenon is observed among patients with high Let-7 expression; Let-7 overexpression in ovarian carcinoma cell lines reduces the frequency of ALDH1^+^ cells; (v) Let-7 targets Lin 28 in ovarian cancer cells. According to these findings it was proposed that in double negative regulatory loop involving both Let-7 and Lin 28 maintain cancer stem cells in ovarian cancer [292].

In addition to these two families of microRNAs, other miRs are also aberrantly expressed in ovarian cancer and for some of them their deregulated expression was related to chemoresistance. Among these miRNAs a relevant role is played by miR-214, whose expression is upregulated in ovarian cancer specimens, compared to normal ovarian epithelial cells. Particularly, it was shown that miR-214 upregulation induces cell survival and cisplatin resistance through targeting the 3′ untranslated region of the PTEN, which leads to downregulation of PTEN protein and activation of the AKT pathway [293]. MiR-199 is another miR whose expression is increased in epithelial ovarian cancer tissue, compared to normal ovarian epithelial tissue. It was shown that in ovarian cancer cells miR-199a targets IKKbeta, a factor whose activation is required for NF-kB activation [293]. The study of the expression of these two miRs at the level of cell populations enriched in ovarian cancer stem cells has in part clarified their role in the biology of these cells. Thus, Chen and coworkers identified and purified two populations of epithelial ovarian cancer cells: type I and type II. Type I cells are CD44^+^ and exhibit some properties of cancer stem cells, such as the tumorigenicity in immunocompromised animals, the capacity of forming spheroids; the high expression of stem cell markers such as Oct-4 and SSEA-4; constitutive NF-kB activity; capacity of production and release of inflammatory cytokines; chemoresistance. In contrast, type II CD44^−^ cells are differentiated, chemosensitive tumor cells. Type I cells are able to differentiate into type II cells [294]. In type I cells miR-199a and miR-214 expression is very low, due to the very low expression in these cells of the transcription factor TWIST1; this leads to high expression in these cells of both PTEN and IkkB. In consequence of this pattern of expression of PTEN and IkkB, ovarian cancer stem cells have constitutive NFkB activity which determines a pro-inflammatory and anti-apoptotic environment and an inactive AKT pathway, leading to a slow proliferation. In contrast, in mature type II ovarian cancer cells the high levels of TWIST 1 drives a high expression of miR-199a and miR-214 which downregulate IKKB and PTEN, respectively and, in consequence of it, they do not have constitutive NF-kB activity and have high AKT activity. These cells proliferate faster and are chemosensitive [295].

Other miRNAs are involved in the mechanisms of chemoresistance of ovarian cancer cells. MiR-18a controls another pathway relevant for chemoresistance of ovarian acncer cells. Regulation of the hnRNPA1 RNA-binding protein by either miR-15a-5p or miR-25-3p leads to increased ovarian cancer growth by inhibiting the generation of miR-18a-3p, an inhibitor of KRAS [296]. Alteration of this regulatory circuit causes poor overall survival outcome in ovarian cancer patients. The inhibition of miR-25-3p and miR-15a-5p decreased the proliferation and invasiveness and increased apoptosis when combined with docetaxel [296]. Finally, the evaluation of the miR-18a-3p/KRAS ratio may represent a useful outcome prognostic factor [296]. MiR-136 was significantly downmodulated in in tumor specimens from ovarian cancer patients with chemoresistant disease: particularly, miR-136 expression was significantly reduced in primary platinum-resistant ovarian cancer patients [297]. In platinum-resistant ovarian cancer cell lines, miR-136 overexpression promoted tumor cell apoptosis in response to cisplatin [277]. Other studies showed that miR-136 overexpression inhibits cancer stem cell activity and enhances the anti-tumor effect of paclitaxel against chemoresistant ovarian cancer cells by targeting NOTCH3 [298].

Bmi-1 activation plays a relevant role in the biology of ovarian cancers, as well as of other numerous solid tumors. MiR-15a and miR-16 are underexpressed in HGS-OvCa; these miRNAs target the Bmi-1 3′ untranslated region and significantly correlate with Bmi-1 protein levels in primary ovarian cancer tumor specimens [299]. Bmi-1 protein levels are significantly downregulated in response to miR-15a or miR-16 expression and lead to significant reduction of ovarian cancer cell proliferation and clonal growth [299]. Nanoliposomal delivery of the miR-15a and miR-16 combination, in a pre-clinical model of chemoresistant ovarian cancer, showed a marked reduction in tumor burden compared to cisplatin alone and suggest potential therapeutic applications for these two miRs [300].

The modulation of TWIST1 expression plays an important role in the biology of OCSCs. Purified populations of OCSCs CD44^+^/MyD88^+^ cells are able in vitro and in vivo to differentiate into CD44^+^/MyD88^−^ mature epithelial ovarian cancer cells [301]. The stem population possesses wide differentiation properties as shown by several observations. In fact, depending on the environment, these cells are able to differentiate also into endothelial cells and therefore to act as tumor vascular progenitors [301]. CD44^+^/MyD88^+^ cells are able both in vitro and in vivo to undergo the epithelial to mesenchymal transition and in doing it the generate mesenchymal spheroid-forming cells with metastatic potential and with the capacity of generating tumors in vivo both in peritoneum and ovarian tissue [293]. OCSCs possess low TWIST1 levels due to its proteosomal degradation; low levels of TWIST1 in these cells are important for the maintenance of their stemness. During differentiation of OCSCs to mesenchymal spheroid-forming cells, TWIST1 expression is greatly induced and functional studies indicate that TWIST1 expression is required for this epithelial to mesenchymal transition, but its overexpression in OCSCs cannot induce EMT [231]. TWIST1 expression is stabilized by E12; the expression of both TWIST1 and E12 is induced by hypoxia/HIF-1, thus favoring mesenchymal differentiation of ovarian cancer stem cells [231]. EOC stem cells have an active mechanism to prevent excessive accumulation of TWIST1 protein even in the presence of high levels of TWIST1 mRNA: this regulatory system involves the ubiquitin-proteasome system. When TWIST1 levels increase at the level of ovarian cancer stem cells, there are some important functional consequences for these cells: in fact, a part of these cancer stem cells differentiate into the so-called “migratory ovarian cancer stem cells,” that acquire mesenchymal properties and the capacity to migrate and to originate metastases.

Other studies have shown that Transforming Growth-beta abundantly expressed in ovarian cancer microenvironment maintains a stem cell phenotype of some ovarian cancer cells and stimulates Transglutaminase 2 expression and, through this mechanism, enhances the metastatic potential of ovarian cancer cells by inducing epithelial to ovarian cancer transformation [302].

A subsequent study confirmed the low expression of miR-199a in ovarian cancer-initiating cells and has also shown that this microRNA targets CD44. The low expression of miR-199a into ovarian cancer stem cells helps to understand why CD44 expression is elevate in these cells [303]. Overexpression of miR-199a into these cells greatly inhibited their tumorigenicity in xenotransplantation assays. Another miR involved in the control of stemness of ovarian cancer cells is miR-214; elevated miR-214 levels have been associated with chemoresistance and metastasis. A recent study provided evidence that miR-214 affects the ovarian cancer stem cell properties by targeting p53/NANOG axis: in fact, enforcing expression of miR-214 increases, while miR-214 knockdown decreases, the size and self-renewal of ovarian cancer stem cells [304]. P53 is repressed by miR-214, that through this mechanism controls NANOG levels [304]. Therefore, the overexpression of miR-214 represses p53 expression and though this mechanism stimulates the stemness properties of ovarian cancer stem cells. In contrast, p53 expression inhibits the ovarian cancer stem cell properties.

An integrated genomic analysis showed that a miRNA-regulatory network allowed to define a mesenchymal ovarian cancer subtype, associated with poor overall survival. In this mesenchymal subtype, eight miRNAs, including miR-506, miR-141 and miR-200a were predicted to regulate 89% of the targets in this network [305]. Functional data showed a key role of miR-506, whose expression determines an increased E-Cadherin expression, an inhibition of cell migration and invasion, and prevention of TGFβ-induced epithelial-mesenchymal transition by targeting SNAI2, a transcriptional repressor of E-Cadherin [305]. In primary ovarian cancer samples, increased miR-506 expression was associated with reduced SNAI2 expression, elevated E-Cadherin and beneficial prognosis [305].

Exosomes have emerged as important mediators and regulators of various biological functions related to cancer biology, including tumor suppression, tumor progression, invasion, cell-to-cell communication and immune-escape, through the release and intercellular transmission of key mediators such as proteins, mRNAs and miRNAs. In this context, the miR-6126 and miR-940, both acting as tumor suppressors, are preferentially expressed in exosomes released by ovarian cancer cells, compared to normal ovarian epithelial cells [306,307].

## 13. Mouse Models of Human Epithelial Ovarian Cancer

Preclinical mouse models of ovarian cancer, including genetically engineered mice and xenograft have been developed, with the specific aim of improving our understanding of the cellular and molecular mechanisms underlying the development of ovarian cancer and of providing an experimental in vivo platform to evaluate new drugs. It is also important to note that the various genetically engineered mouse models of HGSC have contributed to the debate about the cellular origin of these tumors either from the ovarian surface epithelium or from the fallopian tube epithelium.

The development of an animal model of ovarian cancer suitable to recapitulate the early lesions and disease progression seen in patients remained for long time elusive. Thus, many studies have used conditional expression of oncogenes such as *KRAS* or conditional deletion of some key tumor suppressors, such as *TP53, Rb, PTEN* and *BRCA1/2*: in all these models, the OSE cell population was targeted by the oncogenic process. Usually, tumors developed into these animals failed to recapitulate the histology and markers, and the tumor progression typical of the human disease. A more recent study utilized an activating *PIK3CA* mutation (the mutation alone induced premalignant hyperplasia of the OSE, but not tumors) coupled with *PTEN* loss in the mouse ovary to drive the formation of ovarian carcinoma from the OSE: the double-mice mutant developed ovarian serous adenocarcinomas, granulosa cell tumors and luteoma tumors [308]. Kima and coworkers provided evidence that, when DICER, an essential gene for microRNA synthesis, and PTEN, a key negative regulator of the PI3K pathway, are conditionally disabled with Amhr2-Cre in mice, high-grade serous adenocarcinoma arise from the fallopian tube [309]. These tumors exhibit histological and molecular similarities to human serous tumors [309]. Although these animals developed tumors in the fallopian tubes, they failed to develop the early tumoral lesions, typical of human disease pathogenesis. More recently, Perets and coworkers have reported the development of a mouse model recapitulating the early phases of human disease [310]. This mouse model is based on the targeting of *BRCA, TP53* and *PTEN* genes: it is important to note that, according to the TCGA data, *BRCA1, TP53* and *PTEN* alterations are prevalent in 25% of cases. These tumors were shown to originate in fallopian tubal secretory epithelial cells and recapitulate the key genetic events and precursor lesions characteristic of human invasive ovarian cancer [310]. Another mouse model of ovarian cancer derived from fallopian tube is the mogp-Tag transgenic mouse, which expresses the SV40 large T-Antigen under the control of the mouse mullerian-specific Ovgp-1 promoter. The mice spontaneously develop neoplastic lesions in the fallopian tube epithelium and endometrium. Importantly, histological analysis of the fallopian tubes of these mice identified a variety of neoplastic lesions reminiscent of human serous tubal intraepithelial carcinoma (STIC), a precursor of ovarian HGS-OvCa [311]. Therefore, this mouse model displays a progression from normal tubal epithelium to invasive HGS-OvCa, and then represents a potentially important experimental model to study human ovarian HGS-OvCa pathogenesis [311].

The expression of mutated *BRCA1, TP53, RB1*, and *NF1* genes in oviductal epithelium, the murine equivalent of human fallopian tube epithelium results in serous tubal intraepithelial carcinomas (STICs) that progress to HGSC or carcinosarcoma [311]. The cancer phenotype is highly penetrant and more rapid in mice carrying engineered alleles of all four tumor suppressor genes. *BRCA1, TP53* and *PTEN* inactivation in the oviduct also results in STICs and HGS-OvCas, and is associated with diffuse epithelial hyperplasia and mucinous metaplasia, which are not observed in mice with intact PTEN [309]. Oviductal tumours arise earlier in these mice than in those with *BRCA1, TP53, RB1* and *NF1* inactivation. Tumor initiation and/or progression in mice lacking conditional PTEN alleles probably require the acquisition of additional defects. These models closely recapitulate the heterogeneity and histological, genetic and biological features of human HGS-OvCa [311].

Other mouse models of ovarian cancer were based on xenotrasplant assays. In these assays, human ovarian tumor cells are transplanted either under the skin (subcutaneously), into the abdominal cavity (intraperitoneally) or into the organ of origin (ovary) of an immunocompromised host (NOR or SCID mice). For the orthoptic model of human tumor ovarian cells there is the unique opportunity to inject these cells into the bursa encapsulating the ovaries and fallopian tubes in mice. Initial studies have been based on the injection of tumor cells derived from human ovarian cancer cell lines and basically have shown that in many cases these transplants were able to reproduce the spread to ascites, liver and spleen observed in human disease (reviewed in [312]). An alternative to the traditional cell line-derived xenograft models implies the isolation and injection of tumor fragments derived from single patients into immunodeficient mice. The xenotransplantation of fresh human tumors more accurately reflects the primary patient tumor and may help to predict response to treatment. Reports concerning the systematic developments of ovarian cancer xenografts from primary tumors have been published only recently. In this context, particularly relevant was a study from Weroha and coworkers reporting the successful engraftment (74% engraftment rate) of small fragments (0.3–0.5 cm^3^) of primary ovarian cancers into the peritoneum of SCID mice [299]. The tumografts exhibited genomic abnormalities and gene expression features highly comparable to those observed in the corresponding primary tumors [313]. Another recent study reported the frequent (83% engraftment rate) engraftment of primary tumor fragments derived from HGSC patients through subcutaneous or intra-ovarian bursal sites injection into NOD/SCID IL2Rgamma (null) mice [300]. These tumor xenografts displayed an in vivo platinum sensitivity highly consistent with patient outcome [314]. Dobbins and coworkers have comparatively evaluated four different routes of injection of primary ovarian tumor fragments into SCID mice: subcutaneous, mammary fat pad, intra-peritoneal and sub-renal capsule and observed that the subcutaneous way was accompanied by the most successful rate of human engraftment (about 85%) [315]. The xenotumors were composed by heterogeneous cell populations recapitulating the cellular heterogeneity of the tumors from which they were derived and were composed by tumor-initiating cells and by more differentiated tumor cells. These tumorgrafts recapitulate a response to drugs similar to the clinical response to chemotherapy [315]. Treatment with anticancer drugs of these tumorgrafts enriched the frequency of cells possessing tumor-initiating properties [182]. Ricci and coworkers have developed a panel of patient-derived EOC-xenografts recapitulating the cellular and molecular heterogeneity of various ovarian cancers. 25% of the EOC-xenografts were successfully established, either subcutaneously or intraperitoneally, into nude mice. The xenografts were histologically similar to primary patient’s tumor [316]. After orthoptic transplantation into the bursa of the mouse ovary, these tumors exhibited a tissue dissemination highly comparable to that observed for the human disease, with first spreading into the peritoneal cavity and then ascite production [316]. From a molecular point of view the tumorgrafts showed a mutational profile highly comparable to the patient tumor and usually exhibited a higher frequency of copy number alterations than the corresponding patient tumors, reflecting a high genomic instability [316]. Recent studies have reported the development of panels of comprehensively characterized panels of patient-derived-xenografts representative of HGS-OvCa and suitable for the evaluation of new drugs [317,318]. Interestingly, a recent study reported the development of patient-derived-xenograft platform to study BRCA-deficient cancers [319]_._ This orthoptic PDX model emulates the natural progression of HGS-OvCa, with the initial development of primary ovary tumors, followed by metastasis to abdominal viscera. Importantly, the response of these patient-derived-xenografts correlated to that observed in each patient in the clinical setting [320]. The typical molecular features of homologous recombination-deficient tumors were maintained in these patient-derived-xenografts [320].

In addition to xenografts, three-dimensional organotypic culture systems have been developed for the culture of ovarian cancer cells. To this end, Kenny and coworkers have developed a three-dimensional organotypic culture of ovarian cancer (involving the growth of ovarian cancer cells in a multilayer culture, containing fibroblasts, mesothelial cells and extracellular matrix) that can be used to predict the response in vivo to drugs, in terms of effects on cell adhesion, invasion and growth [321].

Not all the ovarian cancer biopsies are able to grow in recipient mice after xenotransplantation. This finding was systematically observed in all studies and offered the opportunity to evaluate a possible link between tumor growth in xenotransplantation assay and biological properties of ovarian cancers. Thus, two recent studies based on xenotransplantation of patient’s derived fresh ovarian tumor tissues xenografted either subcutaneously or in the subrenal capsule showed that patients whose tumors became engrafted have reduced progression survival and overall survival, compared to patients whose tumors failed to engraft [322,323]. These observations suggest that xenograft models can be useful as a preclinical tool to predict prognosis.

In conclusion, these studies have shown that the development of more accurate animal models was in part achieved and this is of fundamental importance to provide in vivo testing platforms for evaluating new drugs and to get advance in our understanding of the disease origin, pathogenesis, progression and treatment resistance. Patient-derived-xenografts maintain the characteristics of the patients’ original tumor, including histology, mutational status, gene expression, and clinical behavior, remaining virtually stable through numerous passages in mice. Therefore, they represent a fundamental evolution of the cell line xenograft model, much more suitable for evaluating tumor heterogeneity and for translation purposes. Particularly, the development of suitable tumor xenograft animal models established from individual primary tumors enables the preclinical evaluation of new molecularly targeted therapeutic agents as they become available and to evaluate the sensitivity of various ovarian cancer subtypes, characterized according to their molecular abnormalities. The progress of the tumor xenograft methodologies has allowed the development of the so-called “avatar” medicine, i.e., personalized tumorgrafts, which can be used as therapy testing surrogates for individual patients, prior or during tumor treatment [324]. The study of tumor xenograft model recently allowed the identification of new potential therapeutic strategies for HGS-OvCas [325,326].

## 14. Circulating Cell-Free DNA and Circulating Tumor Cells: The “Liquid Biopsies” in Ovarian Cancer Patients

The development of screening studies on genomic and gene expression abnormalities occurring in ovarian cancer have certainly given an important contribution to our understanding of the molecular pathogenesis of ovarian cancer, have led to the identification of potential therapeutic targets and opened the way to the development of a personalized ovarian cancer genomics. A recent study showed a very interesting methodology that could considerably simplify the identification of individual ovarian cancer mutations using a noninvasive methodology. In fact, this method was based on the observation that plasma of cancer patients contains cell-free tumor DNA that could isolated and studied for the characterization of tumor mutations [327]. This method, that should be applied also to other tumors, could facilitate the analysis of circulating DNA as a noninvasive “liquid biopsy” for identification and monitoring of cancer mutations.

Subsequent studies have shown that elevated levels of circulating DNA were detected in the plasma of ovarian cancer patients compared to normal healthy subjects or to patients with benign ovarian tumors. Concerning the diagnostic impact of circulating DNA, a recent meta-analysis englobing 9 recent studies on this topic showed that this methodology had 70% of sensitivity and 90% of specificity, thus indicating an acceptable specificity, but a limited sensitivity [328]. Importantly, circulating DNA analysis is a sensitive tool for the evaluation of copy number alteration and chromosome instability, two important hallmarks of HGS-OvCa [329]. The evaluation of circulating DNA is also a tool for the monitoring of the response to therapy [330]. Finally, the analysis of circulating DNA may represent a precious tool to evaluate the molecular mechanisms underlying resistance to chemotherapy treatment: thus, circulating DNA analysis showed the occurrence of truncating mutation in RB1 in HGS-OvCas undergoing standard therapy with cisplatin [331].

In the blood of patients with metastatic solid tumors, including ovarian cancers, circulating tumor cells have been detected. However, these circulating tumor cells are not detected in all patients and their number is very low (1 tumor cell per 1 × 10^6^ nucleated blood cells or less) [332]. The analysis of these cells requires their enrichment using different methods based on various parameters [332]. Recent studies on this topic have suggested that the detection of circulating tumor cells in ovarian cancer patients is associated with advanced disease and with a poor prognosis, but these conclusions are inconsistent in the various studies and largely depend on the methodology used to isolate circulating tumor cells [332]. The detection of circulating tumor cells may represent a tool to evaluate the modifications occurring in tumor cells induced by platinum-based chemotherapy [333] and to predict the resistance and outcome to platinum-based chemotherapy [334].

## 15. Treatment Strategies for Ovarian Cancers

The high mortality associated with ovarian cancer is due largely to the inability to detect the disease early and the lack of effective therapeutics for women with advanced and recurrent disease.

The standard ovarian cancer screening strategies are based on transvaginal ultrasound of pelvis and CA-125 estimation. However, various randomized controlled clinical trials evaluating the impact of these screening tests have failed to show any significant reduction in the mortality among screened women, compared to populations of unscreened women. In this context, particularly interesting are the results of a recent screening trial (UK Collaborative Trial of Ovarian Cancer Screening, UKCTOCS) [335]. In this study, more than 200,000 women were randomly allocated to no screening procedures, to annual transvaginal ultrasound screening (USS) or to annual multimodal screening with CA-125 interpreted using the risk of ovarian cancer algorithm (MMS) in a 2:1:1 ratio. At a median follow-up of 11 years, the mortality for ovarian cancer of the control group was 0.34%, 0.30% in the USS group and 0.29% in the MMS group [335]. An analysis with the exclusion of prevalent cases showed a mortality reduction of about 205 in the MMS group, compared to the unscreened group [335]. These findings support the hope that deaths from ovarian cancer can be reduced through early detection. More generally, this study supports the idea that the improvement in screening procedures, in terms of detecting early disease, and the identification of early disease biomarkers represents a fundamental strategy in future developments aiming to improve the management of this cancer. Whether MMS could be recommended on economic grounds would depend on the confirmation and size of the mortality benefit at the end of the ongoing follow-up of the UKCTOCS trial [336].

The epidemiological studies carried out on large populations of ovarian cancer patients were also of fundamental importance to define some factors related to disease outcome. Thus, the analysis of factors associated with long term survival was of fundamental importance. In this context, some studies have provided clear evidence that for women with invasive ovarian cancer, the presence of *BRCA1* or B*RCA2* mutation was associated with an initial better prognosis, but does not lead to a long-term survival benefit [337]. The absence of residual disease following primary debulking surgery was the most important predictor of death in both BRCA-mutation carriers or not carriers [338]. These epidemiological studies showed also that parity or the cumulative number of ovulatory cycles increased survival, while smoking and Body Mass Index decreased overall survival following the diagnosis of ovarian cancer [339].

The second strategy, as mentioned above, would consist of improving the medical treatment for ovarian cancer patients with advanced or recurrent disease. The development of this strategy needs a better understanding of the cellular and molecular basis of ovarian cancer development. The dramatic progresses made in the last two decades in the understanding of the molecular abnormalities observed in HGS-OvCas have shown that this neoplasia is marked by surprisingly few recurrent mutations, with *TP53* gene being mutated in virtually all cases, but by a very high genetic complexity underlined by very numerous copy number alterations. Furthermore, inter-tumoral and intra-tumoral heterogeneity in HGS-OvCa increases the complexity and heterogeneity of these tumors, thus making unlikely the possibility of finding a single treatment beneficial for all patients. In spite these consistent limitations, the characterization of the molecular abnormalities of HGS-OvCas offered some opportunities for the development of new therapeutic strategies. Thus, about 50% of HGS-OvCas are characterized by mutations at the level of genes involved in the homologous recombination pathway of DNA repair, especially *BRCA1* and *BRCA2*. Preclinical data have provided a clear rationale for the potential clinical utility of PARP inhibitors in the management of HGS-OvCa patients who have known mutations (germline or somatic) in *BRCA*. Clinical experience, both in the phase II and phase III trial settings, has confirmed substantial biological and clinical activity for this class of antineoplastics in disease management. Furthermore, there is evidence for the utility of PARP inhibitors in ovarian cancers in the absence of BRCA mutations, presumably resulting from other deficiencies in the molecular machinery of DNA homologous recombination repair. Thus, three different PARP inhibitors have been introduced into the clinic for the treatment of HGS-OvCa, Olaparib, Rucaparib and Nizaparib. Although these three drugs seem to have different toxicity profiles, to date there have been no direct trials comparing among these drugs. Future, ongoing clinical trials in HGS-OvCa patients will evaluate new treatment strategies based on the combination of PARP inhibitors with standard antineoplastic agents (platinum and taxane) and with other antineoplastic agents, such as checkpoint inhibitors or antiangiogenic agents.

In this context, particularly promising is the targeted drug combination involving Dediranib, a pan VEGFR 1–3 inhibitor and the PARP inhibitor Olaparib, showing a high response rate and progression-free survival in women with HGS-OvCa [340,341]. Basically, this study showed a progression-free survival superior for the combination of Cediranib and Olaparib compared with Olaparib alone in patients with recurrent platinum-sensitive HGS-OvCa in a randomized open-label study, with a recently accrued follow-up: in the primary analysis a median PFS of 17.7 months was observed for the drug combination and 9.0 months for single-drug treatment; in the updated analysis, the PFS was 16.5 months for drug combination and 8.2 months for single-drug treatment [342]. Interestingly and intriguingly, for HGS-OvCa patients with a known BRCA germline mutation, the PFS was similar in the two treatment arms (16.5 months vs. 16.4 months, for double vs. single-drug treatment, respectively); however, for HGS-OvCa patients without a known germline mutation, PFS was clearly superior in the Cediranib/Olaparib arm, compared to Olaparib alone [342]. In this study, it was observed also a trend toward improved survival in patients randomized to Olaparib/Cediranib (44.3 months) vs. Olaparib monotherapy (33.2 months) [342]. It was hypothesized that the mechanism driving synergy could be related to the induction of hypoxia induced by the VEGF inhibitor, with downregulation of genes of homologous recombination: patients who are germline BRCA mutation carriers, are HR-deficient with vulnerability to PARP inhibitors alone; in contrast, BRCA-WT patients do not have an intrinsic HR deficiency and treatment with the VEGFR inhibitor induces HR-deficiency in their tumors with consequent sensitization to the PARP inhibitor [341,342].

As mentioned above, clinical studies of association of PARP inhibitors with a pan-VEGFR inhibitor support the importance of VEGF targeting in ovarian cancer therapy. Recent experimental studies support the therapeutic targeting of VEGF and its receptors. Thus, Jang and coworkers have shown that VEGFA stimulates stem-like cells in ovarian cancer via VEGFR2-dependent Src activation, to upregulate Bmi1, promoting tumor sphere formation and ALDH1 activity [343]. Another study provided evidence that VEGF family members are overexpressed in therapy-resistant ovarian cancer cells; particularly, VEGFR2 overexpression correlated with platinum resistance and VEGFR2 inhibitors rescued a platinum sensitivity of these resistant cells [344]. Moreover, a pan-VEGF-inhibitor synergistically enhanced anti-tumor effects of EGFR-directed therapies including erlotinib [344].

Unfortunately, as for other antineoplastic agents the development of primary and secondary resistance mechanisms limits the extent of clinical benefits deriving from the use of PARP inhibitors. Direct and indirect mutational events can restore an efficient DNA repair, via acquired reversion mutations in *BRCA1* and *BRCA2*, reversion of germline *BRCA1/BRCA2*, loss of *BRCA1* promoter methylation and multiple independent reversion events and can limit the efficacy of PARP inhibitors [345]. Strategies to bypass this resistance may imply alternate dosing of PARP inhibitors or combined administration with platinum derivatives [346].

The clinical activity of PARP inhibitors against ovarian cancers with a dysfunctional homologous recombination pathway, such as those with *BRCA1/2* mutations, has led to the approval of PARP inhibitors, such as Olaparib, for the treatment of *BRCA1/2*-mutated advanced epithelial ovarian cancers [347]. Given these findings, therapeutic strategies are under investigation to try to sensitize BRCA wild-type ovarian cancers to PAPRP inhibitors. In this context, two recent studies have shown the synergy of PARP inhibition with BET bromodomain inhibition in BRCA-proficient ovarian cancer cells, due to induction of mitotic catastrophe [348].; this drug combination induced an increase in the accumulation of DNA damage, checkpoint defects, progression to mitosis and, finally, mitotic catastrophe [348,349]. In xenograft models, the drug combination resulted in an efficient inhibition of the growth of BRCA-proficient ovarian cancers [348,349]. These observations support the view that a combination of BET inhibitor and PARP inhibitor represents a potential therapeutic strategy for BRCA-proficient cancers.

As above repeatedly mentioned, about 20% of HGS-OvCas exhibit cyclin E1 gene amplification and these tumors are usually chemoresistant and associated with a poor prognosis. Various studies carried out in the last years suggest that HGS-OvCas with cyclin E1 overexpression may represent a tumor subset, potentially amenable to therapies with CDK inhibitors, AKT inhibitors or proteasome inhibitors [350].

As observed by all investigators who have characterized HGS-OvCas, TP53 is largely the most frequently mutated gene in these tumors, being mutated in virtually all cases. Given this finding, mutated TP53 would represent an ideal therapeutic target in HGS-OvCa. It is of interest to note that some common cancers, such as HGS-OvCa, triple-negative breast cancer, squamous cell lung cancer and small-cell lung cancer, are all characterized by high frequency (at least 80%) of *TP53* mutations and are all among the most difficult-to-treat. This is not surprising since TP53 is traditionally considered as undruggable. However, recent efforts have been made to develop drugs active against mutant TP53 protein and able to convert it to a molecular form exhibiting WT TP53 activity. Particularly, two compounds have been developed: APR-246, a methylated derivative and structural analogue of PRIMA-1 (p53 Reactivator and Induction of Massive Apoptosis-1) and a third generation thiosemicarbazone developed by Critical Outcome Technologies Inc. (Boston, MA, USA) (COTI-2) [351]. Interestingly, APR-246 covalently modifies the core domain of TP53 mutants through the alkylation of thiol groups, restoring both the wild-type conformation and function to mutant TP53, restoring its normal function, with consequent cell-cycle arrest and induction of apoptosis. COTI-2 targets the misfolded mutant forms of TP53 protein, inducing their conformational modification, with consequent restoration of their normal activity; furthermore, COTI-2 possesses an inhibitory effect on AKT2 activation. APR-246 is under investigation in the context of a phase II in relapsed HGS-OvCa, administered together with Carboplatin and Liposomial Doxyrubicine. Importantly, the phase Ib study carried out with APR-246 administered in association with Carboplatin and Liposomial Deoxyrubicine, showed preliminary promising results, with a progression-free survival of 316 days. These observations, presented at the 2016 Annual Meeting of the European Society for Medical Oncology, support further clinical development for this drug in advanced HGS-OvCa patients.

Another area of potential implication in the development of new therapeutic strategies is related to anti-tumor immunotherapy, particularly with immune check inhibitors. However, although in ovarian cancer there is clear evidence that the presence in the tumor of an immune microenvironment influences disease outcomes, the initial results of various clinical trials based on the use of immune check inhibitors in ovarian cancer patients have shown limited tumor responses [352,353]. In future studies, the integration of genomic profiling with immune profiling would help to identify ovarian cancer patients who can benefit from treatment with various immunotherapy agents. Finally, immunotherapeutic agents may be evaluated in association with other drugs (i.e., in association with PARP inhibitors in HRD HGS-OvCas).

The identification of new therapeutic targets is another fundamental area of research in the context of basic studies on ovarian cancer with translational aims. The identification of these targets requires the study of ovarian cancer primary specimens or tumor xenografts using high-throughput technologies. In this context, a recent phosphoproteomic study of primary ovarian cancer cells revealed druggable kinase signatures [354]. Particularly, this study provided evidence that CDK7 could play a relevant role in ovarian cancer development: CDK7 phosphorylation activates its target, RNA polymerase II (POLR2A), a property specifically observed in ovarian cancer cells, but not in their normal counterpart [354]. These findings identify CDK7 as a potential therapeutic target, though the use of specific chemical inhibitors, such as THZ1 [354].

As outlined in the section on ovarian cancer stem cells, the studies on these cells have offered only very limited tools for translational studies. Particularly, the studies on the identification of cancer stem cell membrane markers do not provide suitable therapeutic targets, due to the great variability in their expression and to the plasticity of cancer stem cells. Therefore, most of the studies were focused on blocking signaling pathways or genetic programs that fuel stemness. In this context, epithelial to mesenchymal transition is an important pathway required for metastasis and the related signaling pathways, such as WNT and TGFβ; similarly, the Hedgehoh and NOTCH pathways are additional important targets for their role in cancer stem cell self-renewal [355]. The identification of specific metabolic abnormalities may offer the opportunity for the selective functional pharmacological targeting of these cells. Thus, studies carried out in cancer stem cells of other solid tumors have shown a peculiar metabolic role of glycolysis, fatty acid synthesis and lipogenic pathways. In this context, particularly interesting was a recent study reporting the characterization of lipid unsaturation in ovarian cancer stem cells by chemical imaging of single living cells through hyperspectral-simulated Raman scattering microscopy [356]. This single-cell imaging study and mass spectrometry analysis showed a clearly increased level of lipid unsaturation in ovarian cancer stem cells, compared to non-cancer stem cells [356]. Importantly, inhibitors of lipid desaturases, exerted a suppressive effect of cancer stem cell properties, including tumor sphere formation and tumor-promoting activity in vivo [356]. NF-kB activation plays an essential role in the inhibitory activity exerted by lipid desaturase inhibitors on cancer stem cells [356]. Interestingly, clinically suitable desaturase inhibitors are available since these drugs have been developed as experimental drugs for diabetes treatment.

## 16. Conclusions

Epithelial ovarian cancer is a highly heterogeneous disease and the development of molecular studies has provided the basis for a more rationale and unified classification of these tumor. Next-generation/high throughput sequencing and gene expression studies now provide a molecular characterization of histological subtypes, with the identification in some patients of suitable therapeutic targets.

HGS-OvCa accounts for most of the morbidity and mortality related to ovarian carcinoma and is one of the leading causes of cancer death in women. The studies carried out in the last two decades have allowed dramatic progresses in our understanding of the cellular and molecular basis of HGS-OvCa. Thus, the origin of these tumors has been clarified showing the existence of precursor lesions in the fallopian tube, facilitating the development of prevention strategies for both patients with inherited susceptibility and with sporadic disease. However, the early diagnosis of HGS-OvCa remains largely unsatisfactory and an improvement in the imaging detection capacity and the identification of new early biochemical biomarkers of the disease is absolutely required to increase the percentage of patients who could be diagnosed at earlier stages of tumor development.

These studies have shown that HGS-OvCa often starts in the Fallopian tube as STIC; particularly, the fimbria is the anatomical site of origin of many HGS-OvCas. The precursor lesion STIC and the p53-signature lesion (a histologically normal, but TP53-mutant Fallopian tube lesion which is the presumed precursor of STIC) are the origin of most cases of HGS-OvCa. Human Fallopian tube cells transformed in vitro generate tumors recapitulating all the histological, immunophenotypic and gene expression features of HGS-OvCas [229]. Not all the HGS-OvCas originate in the tube, but a part of them derive from endosalpingiosis, a process derived from the ectopic presence of tubal cells in the ovary forming microscopic and macroscopic tubal-type cortical inclusion cysts (CICs). These CICs may represent the non-tubal origin of some HGS-OvCas: the epithelium lining CICs may be tubal or surface ovarian epithelium-type or mixed; CICs with tubal epithelium may derive by detachment and implantation of fimbrial epithelium, while CICs with ovarian surface epithelium may derive from invagination of this epithelium.

Ovarian cancer development may be sporadic or may have a familial inheritable component, in the context of hereditary breast and ovarian cancer syndrome. Mutations in BRCA1 and BRCA2 are responsible for about 10–15% of all HGS-OvCa. The lifetime ovarian cancer risk for a woman with *BRCA1* mutation is estimated to be 35% to 70%, the ovarian cancer risk lifetime for women with *BRCA2* mutation is between 10 and 30%; the ovarian cancer lifetime risk for the women in the general population is less than 2%. The high-penetrance genes account for 36% of ovarian cancer familial relative risk. The rest is due to more common, lower-risk genetic variation. Particularly, about 30 susceptibility alleles have been identified in European populations, associated with weak to moderate risk and associated with all ovarian cancer subtypes or with specific subtypes [64].

The development of molecular studies has led to an elucidation of the molecular events important in HGS-OvCa development. The molecular analyses have shown that these tumors are characterized by a high degree of genome/chromosome instability and by the ubiquitous mutations in the tumor suppressor/regulator TP53. In addition, high rates of somatic and germline defects in BRACA1/2 and other homologous recombination genes have led to the identification of a subset of HGS-OvCa patients who can benefit from treatments based on PARP inhibitors. Thus, HGS-OvCa patients with homologous recombination defects may have some benefits in terms of progression-free survival and a limited benefit in terms of overall survival from the treatment with PARP inhibitors. Future studies will define the optimal PARP inhibitors to be used and the most active combination therapies with other agents, including immunotherapeutic agents. The combination of other therapeutic agents (such as antiangiogenic agents) with PARP inhibitors may offer a strategy to increase the sensitivity of BRAC-proficient ovarian cancers to PARP inhibitors and to improve their therapeutic response.

CCNE1 amplification is observed in about 20% of HGS-OvCa and allows the identification of a tumor subgroup characterized by poor prognosis and limited response to standard treatments. Since no clinically suitable Cyclin E1 inhibitors are available, the obvious target for CCNE1-amplified HGS-OvCas is represented by CDK2, the kinase partner of Cyclin E. Pre-clinical observations show the drug sensitivity of *CCNE1*-amplified HGS-OvCa to drug combinations containing a CDK2 inhibitor, such as dinaciclib. Importantly, since Cyclin E1 gene is co-amplified with the AKT2 gene in HGS-OvCa, it is not surprising that CDK2 and AKT2 inhibitors resulted in a strong, synergistic antitumor activity against *CCNE1*-amplified HGS-OvCas and hold promise for possible clinical developments.

The developments in molecular studies have also helped to define the molecular basis of type I ovarian cancers, including LGS-OvCa characterized by frequent *BRAF, KRS, PTEN* and *PIK3CA* gene alterations, Ovarian Clear Cell Carcinoma characterized by frequent *ARID1A* and *PIK3CA* gene alterations, Endometrioid Ovarian Cancers characterized by frequent *ARID1A, PTEN, CTNNB1* and *PI3KCA* gene alterations and Mucinous Ovarian Carcinomas characterized by frequent *KRAS, TP53* and *CDKN2A* gene alterations.

The elucidation of the molecular abnormalities of epithelial ovarian cancers helped to define the site of development of precursor lesions and allowed to understand the mechanisms underlying the process of natural tumor clonal evolution and of drug-induced clonal selection, eventually associated with chemoresistance. Furthermore, the molecular studies have been of fundamental importance to define not only the inter-tumor heterogeneity, but also the intra-tumor heterogeneity, a great challenge to the development of curative treatments for advanced disease.

The development of next-generation/high throughput sequencing technology offered the opportunity to characterize some genes responsible for drug resistance and this approach could be applied at single patient level. Although the perspectives offered by such an approach are theoretically very wide, its application is strongly limited by the low number of “druggable” genetic alterations and the occurrence of intra-tumor heterogeneity and clonal diversity, requiring a multi-target therapeutic approach.

Although the considerable development of this important background of molecular and cellular informations on HGS-OvCa do not yet have been translated into consistent therapeutic improvements, we are confident that this progress will offer in relatively not very long times the way for significant improvements, at least in some subsets of epithelial ovarian cancer patients.

Studies carried out in the last years do not attempted only to identify new therapeutic approaches for epithelial ovarian cancers, but aimed also to a better standardization of the current therapy and to the definition of the response to this therapy. Surgery remains the cornerstone of treatment for HGS-OvCa, preceded or followed by chemotherapy. In patients who undergo surgery before they receive adjuvant chemotherapy (ACT), the strongest prognostic factor is complete disease resection. For patients receiving neoadjuvant chemotherapy (NACT) treatment before surgical resection, the histopathological evaluation (based on sixth- or three-degree system) of the residual tumor before surgical intervention represents a reliable system to predict PFS and OS [64]. This evaluation system is important to identify categories of patients at moderate/high-risk of relapse and short survival [357].

Ovarian cancers contain a subpopulation of stem-like cells that has the ability to grow in vitro in an anchorage-independent manner and are able in vivo to generate the tumoral process. Several putative ovarian cancer stem cell markers, such as CD24, CD44, CD133, SOX2, SSEA have been proposed, but the definition of their phenotypical features remains highly variable in the various studies, probably due to the consistent phenotypic and functional plasticity of these cells. The existence of highly-tumorigenic and chemotherapy-resistant cancer stem cell-specific biomarkers open the way to the targeting of these cells to minimize the drug resistance and the tumor relapse. However, now, the only impact consequent to the identification of cancer stem cell-related biomarkers is linked to the identification of some prognostic markers.

## Figures and Tables

**Table 1:** Main features of the various types of ovarian tumors.

Tumor	Type	Cells of Origin	Precursor Lesion	More Frequent Mutations	Familial Risk
Endometrioid Cancer	I	Endometrial epithelial cells	Endometrioid borderline tumor	ARID1A, PIK3CA, TERT	Lynch syndrome
Ovarian Clear Cell Carcinoma	I	Endometrial epithelial cells	Endometrioid borderline tumor	ARID1A, PIK3CA, PTEN, CTNNB1, KRAS, TP53, RPL22	Lynch syndrome
Mucinous Carcinoma	I	Unknown	Cystadenoma, Mucine borderline tumors, Brenner Tumors	KRAS, TP53, CDKN2A, BRAF, RNF43	Unknown
Brenner Tumor	I	Transitional-like cells of fallopian tube	Benign Brenner tumor	Sporadic point mutations MYC amplification	Unknown
Low-Grade Serous Carcinoma	I	Fallopian tube progenitor cell or secretory epithelial cell	Serous borderline tumors	KRAS, NRAS, BRAF, EIF1AX, USP9X, FFAR1, NF1, HRAS	Unknown
Seromucinous Carcinoma	I	The same as for endometrioid, Low-grade serous and mucinous carcinomas	The same as for endometrioid, Low-grade serous and mucinous carcinomas	KRAS, PIK3CA, PTEN, ARID1A	Unknown
High-Grade Serous Ovarian Cancer	II	Fallopian tube progenitor cell or secretory epithelial cell	STIC, SCOUT, TP53 signature	TP53, BRCA1, BRCA2, CNAs of CCNE1 amplification, PTEN deletion, RB1 and NF1 loss	BRCA1, BRCA2, BRIP1, PALB2, RAD51C and RAD541D
Ovarian Carcinosarcoma or Malignant MullerianMixed Tumors	II	Unknown	Unknown	TP53, PI3KCA, PPP2R1A, KRAS, PTEN, CHD4, BCOR, histone H2A and H2B	Unknown
Ovarian Granulosa Cell Tumors	Adult Juvenile	Sex-cord stromal Cells (granulosa cells)	Unknown	FOX2L in 87% of adult-type patients	Unknown
Sertoli-Leydig Cell Tumor	NA	Granulosa cells or other Stromal cells	None	DICER1	DICER1 syndrome
Small Cell Carcinoma of The Ovary, hypercalcemic Type	NA	Rare resident ovarian Cells	None	SMARCA4	RTPS2 (SMARCA4)
Fibroma	NA	Ovarian stromal cells	None		None

**Table 2:** Tumor-node-metastasis (TNM) and International Federation of Gynecology and Obstetrics (FIGO) classifications. The 5-year survival for the various stages is reported.

Primary Tumor	FIGO	Tumor Extension	5-Year Survival Rate
T0		No evidence of primary tumor	N.A.
T1	I	Tumor limited to the ovaries (one or both)	85–94%
T1a	IA	Tumor limited to one ovary; capsule intact, no tumor on ovarian surface; no malignant cells in ascites or peritoneal washings.	94%
T1b	IB	Tumor limited to both ovaries; capsule intact, no tumor on ovarian surface; no malignant cells in ascites or peritoneal washings.	92%
T1c	IC	Tumor limited to one or both ovaries with any of the following: capsule ruptures, tumor on ovarian surface, malignant cells in ascites or peritoneal washings.	85%
T2	II	Tumor involves one or both ovaries with pelvic extension.	69–78%
T2a	IIA	Extension and/or implants in the uterus and/other tube(s); no malignant cells in ascites or peritoneal washings.	78%
T2b	IIB	Extension and/or implants in other pelvic tissues; no malignant cells in ascites or peritoneal washings.	73%
T2c	IIC	Pelvic extension and/or implants (T2a or T2b) with malignant cells in ascites or peritoneal washings.	69%
T3	III	Tumor involves one or both ovaries with microscopically confirmed peritoneal metastasis outside the pelvis.	17–59%
T3a	IIIA	Microscopic peritoneal metastasis beyond the pelvis (no macroscopic tumor).	59%
T3b	IIIB	Macroscopic peritoneal metastasis beyond the pelvis 2 cm or less in greatest dimension.	39%
T3c	IIIC	Macroscopic peritoneal metastasis beyond the pelvis >2 cm in greatest dimension and/or regional lymph node metastasis.	17%
T4	IV	Malignant cells in pleural effusion, parenchymal metastases and or metastases to extra-abdominal organs (including inguinal lymph nodes and lymph nodes outside of the abdominal cavity).	12%

**Table 3:** Gene expression classification of HGSOCs.

Subtype	Main Features	Genes Expressed	Main Pathways	Clinical Outcome
C1/Mesenchymal (28%)	Extensive myofibroblast infiltration (desmoplasia). Mesenchymal gene expression signature	↑ COLL11A1, CXCL14, POSTN, SNAIL2, VCAN, ZEB1	Focal adhesion ECM receptor interaction JAK-STAT signaling TGF-β signaling VEGF signaling Fibroblast signature EMT/Stem cell	Negative prognosis
C2/Immunoreactive (21.5%)	Extensive intratumoral T lymphocyte infiltration	↑ CXCL10, CXCL11, PSMB8, PSMB9, TAP1	T-cell receptor signaling Toll-like receptor Antigen presentation machinery	Better prognosis
C5/Proliferative (20.5%)	Low expression of differentiation markers, limited inflammatory infiltration, activation of signaling pathways involving oncogenic and stem cell factors Lower BRCA1-2 mutation rate	↑ HMGA2, SALL2, SOX11, TCF7L1	Cell cycle NOTCH signaling	Negative prognosis
C4/Differentiated (17.5%)	Gene signature resembling serous borderline tumors	↑ COLEC11, DEFB1, ITGB4, MGLL, MLPH, STAR	Ribosome Metabolism Cytochrome p450	Intermediate prognosis
C4/Anti-mesenchymal (12.5%)	Downregulation of the genes typically upregulated in the mesenchymal subtype Lower BRCA1-2 mutation rate	↓ COLL11A1, DCN, FAP, POSTN, VCAN, ZEB1	Oxidative phosphorylation Peroxisome Butanoate metabolism	Better Prognosis

**Table 4:** Main properties of markers of ovarian cancer stem cells.

Marker	Chromosome Location	Biochemical Properties	Function in Ovarian CSC Biology	Number of Tumorigenic Cells
CD133	4p15.32	Transmembrane glycoprotein It induces PI3K/AKT pathway	Tumor sphere formation, Tumor-initiating capacity, Self-renewal. Chemoresistance. High CD133 expression is a negative prognostic factor.	10^2^ CD133^+^
CD44	11p13	Transmembrane glycoprotein Hyaluronic acid Receptor It stimulates EGFR-Ras-ERK	Tumor sphere formation, Tumor-initiating capacity, Self-renewal. High CD44 expression is associated with TNM stage and survival. CD44 promotes epithelial-mesenchymal transition.	10^2^ CD44^+^
CD24	6q21	Transmembrane glycoprotein It activates STAT3	Tumor sphere formation, Tumor-initiating capacity, Chemoresistance. It is involved in ovarian cancer metastasis. High CD24 is a negative prognostic factor.	5 × 10^3^ CD24^+^
CD117	4q12	Transmembrane glycoprotein Kit-Ligand Receptor	Tumor sphere formation, Tumor-initiating capacity (particularly CD117^+^/CD44^+^), Self-renewal. Chemoresistance through Wnt/β-catenin activation. High CD177 expression is a negative prognostic factor.	10^3^ CD117^+^ 10^2^ CD44^+^/CD117^+^
CXCR4	2q22.1	Transmembrane glycoprotein SDF-1α Receptor	Tumor sphere formation, Tumor-initiating capacity. CXCR4^+^/CD133^+^ cells are chemoresistant. The number of CXCR4^+^/CD133^+^ cells is highly variable in primary tumors.	10^3^ CXCR4^+^/CD133^+^
CD151	11p15.5	Membrane Protein Tetraspanin	Tumor sphere formation, Tumor-initiating capacity. It is involved in epithelial-mesenchymal transition and induces chemoresistance. High CD151 expression is associated with poor prognosis.	N.D.
EpCAM	2p21	Transmembrane Glycoprotein Epithelial Cell Adhesion	CD44^+^/CD24^+^/EpCAM^+^ cells promote tumor sphere formation, have tumor-initiating capacity, are chemoresistant. High EpCAM expression is associated with poor prognosis.	10^2^ cells
ALDH1	9q21.13	Enzymatic activity Aldehyde Dehydrogenase	Tumor sphere formation, Tumor-initiating capacity. Total ALDH1 expression correlates with a favorable prognosis. CD133^+^/ALDH1^+^ cells have a negative prognostic impact.	N.D.
SOX2	3q26.33	Transcription Factor It maintains pluripotency and Self-renewal in embryonic Stem cells.	It promotes migration and invasion of ovarian cancer cells. It increases the expression of CSC markers, tumor sphere formation, tumor-initiating capacity. Its expression in HGS-OvCa is associated with negative prognosis.	N.A.
OCT4	6p21.33	Transcription Factor Octamer-Binding Transcription Factor 4	It is required for self-renewal capacity of CSCs. It induces epithelial-mesenchymal transition. It is involved in chemoresistance.	N.A.
NANOG	12p13.31	Transcription Factor Nanog Homeobox	It is required for CSC tumorigenic activity. It is involved in both epithelial-mesenchymal transition and in chemoresistance through STAT3 activation. High NANOG is a negative prognostic factor.	N.A.
